# Differential molecular response in mice and human thymocytes exposed to a combined-dose radiation regime

**DOI:** 10.1038/s41598-022-07166-8

**Published:** 2022-02-24

**Authors:** Pilar López-Nieva, Iria González-Vasconcellos, Laura González-Sánchez, María A. Cobos-Fernández, Sara Ruiz-García, Raúl Sánchez Pérez, Ángel Aroca, José Fernández-Piqueras, Javier Santos

**Affiliations:** 1 Genome Dynamics and Function Program, Genome Decoding Unit, Severo Ochoa Molecular Biology Center, Madrid, Spain; 2 Department of Biology, Madrid Autonomous University, 28049 Madrid, Spain; 3 Institute of Health Research, Jiménez Díaz Foundation, 28040 Madrid, Spain; 4 Consorcio de Investigación Biomédica de Enfermedades Raras (CIBERER), Madrid, Spain; 5 Department of Congenital Cardiac Surgery, Hospital Universitario La Paz, 28046 Madrid, Spain

**Keywords:** Cancer, Cell biology, Oncology

## Abstract

In the quest for more effective radiation treatment options that can improve both cell killing and healthy tissue recovery, combined radiation therapies are lately in the spotlight. The molecular response to a combined radiation regime where exposure to an initial low dose (priming dose) of ionizing radiation is administered prior to a subsequent higher radiation dose (challenging dose) after a given latency period have not been thoroughly explored. In this study we report on the differential response to either a combined radiation regime or a single challenging dose both in mouse in vivo and in human ex vivo thymocytes. A differential cell cycle response including an increase in the subG1 fraction on cells exposed to the combined regime was found. Together with this, a differential protein expression profiling in several pathways including cell cycle control (ATM, TP53, p21^CDKN1A^), damage response (*γ*H2AX) and cell death pathways such as apoptosis (Cleaved Caspase-3, PARP1, PKCδ and H3T45ph) and ferroptosis (xCT/GPX4) was demonstrated. This study also shows the epigenetic regulation following a combined regime that alters the expression of chromatin modifiers such as DNMTs (DNMT1, DNMT2, DNMT3A, DNMT3B, DNMT3L) and glycosylases (MBD4 and TDG). Furthermore, a study of the underlying cellular status six hours after the priming dose alone showed evidence of retained modifications on the molecular and epigenetic pathways suggesting that the priming dose infers a “radiation awareness phenotype” to the thymocytes, a sensitization key to the differential response seen after the second hit with the challenging dose. These data suggest that combined-dose radiation regimes could be more efficient at making cells respond to radiation and it would be interesting to further investigate how can these schemes be of use to potential new radiation therapies.

## Introduction

More than 50% of cancer patients require radiotherapy as part of their cancer treatments and it is frequently used to treat the most common types, such as breast, cervical, colorectal, and lung cancer as stated by the World Health Organization (WHO) and the International Atomic Energy Agency (IAEA). It is sometimes combined with chemotherapy or immunotherapy to boost its results. Advances in radiation therapy in the past decade have led to significant improvements in tailoring the radiation dose distribution, however, radiation exposure still causes cytotoxic effects to healthy tissue^1,2^. If fact, during the last years, many new trials have been set to find practice-changing radiation therapies^3–5^. Therefore, there is a necessity to improve the effectiveness of radiation treatments in order to make them more effective at killing tumour cells yet less harmful to the surrounding healthy tissue. A small conditioning (or priming) dose of ionizing radiation (less than 100 mGy) reduces the genotoxic effects induced by a higher dose (challenging dose) administered after a given latency interval^6^, demonstrating the potential of this combined treatment at preventing healthy tissue damage. Together with this, previous studies showed that T-cell lymphoma induction in mice was reduced by changing the single dose regime (1.75 Gy) to a combined regime (0.075 + 1.75 Gy)^7^ demonstrating that combined radiation schemes protect normal cells from neoplastic transformation. Genome-scale assessment of the combined response in human lymphoblastic cells demonstrated the upregulation of genes involved in DNA repair and stress response while down-regulated genes were associated with cell cycle control and apoptosis in adapting cell lines^8^. Using in vivo mouse models, us and others found that a combined regime of radiation, where the priming low dose was administered as an acute dose^9^ or chronic exposure^10^, could exert a differential effect on apoptosis in cells of the lymphoid line by regulating expression of genes involved in cell death control. This suggests that more information is needed on which cell death mechanisms are induced after a combined radiation treatment and how can they be key to the betterment of radiation therapies. Understanding the activation of the damage and cell cycle control pathways to either a single dose or a combined regime of radiation, would also be pivotal at getting an overall picture of the response to a combined scheme and whether or not this regime could be more useful at cell killing and maintaining healthy tissue homeostasis than fractionating the total treatment dose into challenging doses alone.

Previous studies on low doses have demonstrated its effectiveness at enhancing cancer therapeutics^11^, therefore it seemed the way forward to study the outcome of a combined regime regarding its potential for new therapy regimes.

Some authors have hinted that epigenetics could play a major role in radiation response^12^. These epigenetic modifications result in enduring cellular transformations without altering the underlying DNA nucleotide sequence. Mutations of epigenetic regulatory genes are common in thymic carcinomas^13^ and it is known that high doses of ionizing radiation result in epigenetic modifications in adult mice^14^. In fact, targeted disruption of *Dnmt1* and *Dnmt3a* in cultured cells eliminates the transmission of genomic instability^15^. A high frequency of induction and persistence of IR-induced genomic instability, as well as a non-Mendelian mode of inheritance of transgenerational effects also suggests an epigenetic based mechanism key at transferring molecular information after exposure^16^. Understanding these epigenetic mechanisms within the combined regime will help understanding the overall response of thymocytes and the mechanism of transmission of information between the two doses in a combined radiation therapy.

## Material and methods

### Mice and irradiation schemes

Female C57BL/6 J mice of comparable age (4–5 weeks) and weight (21–24 g) were purchased from Charles River Laboratories. They were kept one week in the local animal house for acclimatization. Animal experiments were carried out according to the European Commission Guidelines (Directive 86/609/CEE) on the use of laboratory animals.

Mice were separated into four experimental conditions to receive whole-body irradiation (Supplementary Fig. 1A): non-irradiated control mice; combined irradiated mice which were exposed to a priming irradiation dose (0.075 Gy of X-rays), generated with a Philips MCN 101 X-ray generator with an interval of 6 h prior to a subsequent challenge irradiation (1.75 Gy of *γ*-rays); mice irradiated with an acute high dose of *γ*-rays 1.75 Gy same as the challenging dose of the combined regime, that were generated by a ^137^Cs gamma IBL-437C irradiator (CIS bio international) and the study of the priming dose effects was performed on mice exposed to an acute single low dose of 0.075 Gy of X-rays. These mice were exposed to 0.075 Gy were kept alive for a latency period of 6 h to study. Each experimental group consisted of three mice. Mice were separated into four experimental conditions (Supplementary Fig. 1A). A 6 h interval between the priming and challenging doses was found essential for the establishment of an effective response to the combined treatment^7,9,17^. A time course of 30 min (acute response), 4 and 6 h were chosen to analyse the initial response to the combined regime when compared to the single dose treatment.

These procedures have the approval of the Community of Madrid (PROEX 22/15), the Ethic Committee of the Spanish National Research Council (CSIC; 308/2015) and the Ethics Committee of Animal Experimentation (CEEA) of the Centre of Molecular Biology Severo Ochoa (Madrid, Spain) (CEEA-CBMSO-23/191).

### Isolation of normal mouse thymocytes

Thymocytes were obtained by removing the thymus from the corresponding irradiated and non-irradiated C57BL/6 mice. Thymuses were washed in PBS and thymocytes were isolated via straining through a Nylon Mesh Cell Strainer of 40 µM (BD Biosciences, San Jose, CA).

### Isolation of normal human thymocytes

Human postnatal thymocytes were isolated from thymuses removed during congenital heart defects corrective surgery of 3 pediatric patients aged between 1 month and 3-year-old. Thymuses were mechanically disaggregated using a cell dissociation sieve-tissue grinder and isolated via ficoll density gradient (Supplementary Fig. 1B). The participants provided written informed consent and study was carried out in accordance with the Declaration of Helsinki.

### Adequacy of the age-specific comparison between mice and humans

Detailed information on the adequacy of the use of an age-specific animal model (four-week-old mice) and its comparison to human pediatric samples (aged between 1 month and 3 years) is provided in the supporting information attached to the paper.

### Cell cycle analysis

Thymocytes were harvested and fixed in ice-cold 70% ethanol. On staining, cells were suspended in PBS containing 50 mg/ml RNase A (Sigma) for 15 min at room temperature. Last, we added propidium iodide (Sigma) staining solution at a final concentration of 50 µg/ml. cell cycle profiles were generated on a FACS-Calibur and Coulter Epics XL-MCL flow cytometer. The experiment was repeated 3 times for each cell type and condition.

### Western blotting

Isolated thymocytes were homogenized in radioimmunoprecipitation assay (RIPA) lysis buffer, supplemented with PhosStop phosphatase inhibitor and Complete EDTA free protease inhibitors (Roche) to isolate the soluble protein fraction. Protein concentrations were determined with the BCA™ protein assay kit (Pierce). Western blotting experiments were conducted at room temperature using 5 µg of cell lysate extracts, fractionated by 4–15% precast Long-life TGX (Tris–Glycine eXtended) Gels (Bio-Rad) and transferred to a polyvinylidene difluoride (PVDF) membrane using the Trans-Blot Turbo Transfer System according to the manufacturer’s protocols (Bio-Rad). Membranes were cut around the predicted molecular weight according to the manufacturer´s information (see supplementary Table 1) for each targeted protein following the schemes showed in supporting information. All biological replicates were always run in the same membrane for correct comparison and quantification proposes. After incubation with blocking buffer (5% nonfat milk or 5% BSA in TBST for 60 min), the membrane was washed once with TBST and incubated with primary antibodies 4 °C overnight at constant moving. Specific details on the primary antibodies used, conditions for blocking and primary antibody dilutions used are contained in Supplementary Table 1. Membranes were washed and incubated with a 1:1000 dilution of horseradish peroxidase-conjugated anti-mouse or anti-rabbit antibodies for 1 h. Blots were washed and developed with the WesternBright (TM) ECL system (Advansta) according to the manufacturer’s protocols. Images were immediately captured on ImageQuant LAS 4000 mini (GE Healthcare) under the same exposure for each complete membrane.

### Blot imaging and densitometric analysis

Software-computer tool (Image Studio Lite Software version 4, LI-COR Biosciences) was chosen to perform pixel quantification of the images. Normalization method is used to correct for differences in protein abundance that are not relevant to the biological question being addressed. A summary of all western blot analysis is depicted on Supplementary Table 4 using the relative expression of the gene to its β-actin for each blot. All complete figures including the β-actin for each blot are shown on the annexed figures within the supporting information. Images were cut and straightened using Adobe Photoshop software CS (Berkeley, CA V21.2.12). Brightness and contrast processing were performed when needed for the whole membrane including all bands presented (controls and exposed samples to the different radiation regimes) using the “auto exposure” feature of Adobe Photoshop software CS (Berkeley, CA V21.2.12). No other processing of the images was performed. The different exposed images are shown in supplementary information for clarification.

### DNA methylation studies

Global methylation: A total of six human samples (n = 3 control samples; n = 3 irradiated with 0,075 Gy) had DNA extracted to quantify DNA methylation levels. Global DNA methylation status was performed using the Methylflash Methylated DNA Quantification Kit (Epigentek, Farmingdale, NY, USA). DNA is bound to high DNA affinity strip wells. Methylated DNA is detected using capture and detection antibodies to 5-methyl cytosine (5-mC) and then colorimetrical quantified by reading absorbance at 450 nm using iMark™ Microplate Absorbance Reader (Bio-Rad Laboratories). The amount of methylated DNA is proportional to the OD intensity measured. The absolute amount of methylated DNA was quantified using a standard curve, plotting the OD values *vs*. 5 serial dilution of control methylated DNA (0.5–10 ng). Specific Promoter methylation: Specific promoter methylation studies were performed on the same batch of samples using the OneStep qMethyl™ Kit (Zymo Research) according to manufacturer’s protocols and with the primers listed on the Supplementary Table 2.

### Statistical analysis

Statistical analyses were performed using GraphPad Prism version 9.3.1 for Mac OS X, (GraphPad Software, San Diego, California USA, www.graphpad.com). Measurement data were expressed as mean ± SD, and were analyzed using the independent samples t-test. One-way ANOVA followed by Bonferroni's post hoc test were used for multiple pairwise comparisons, with an inspection level of α = 0.05. Significance levels such as *p ≤ 0.05; **p ≤ 0.01; ***p ≤ 0.001 and ****p ≤ 0.0001 were considered to indicate a statistically significant difference.

## Results

### Early cell cycle arrest and faster cell death induction in in vivo mice thymocytes exposed to the combined regime

**Figure 1:**
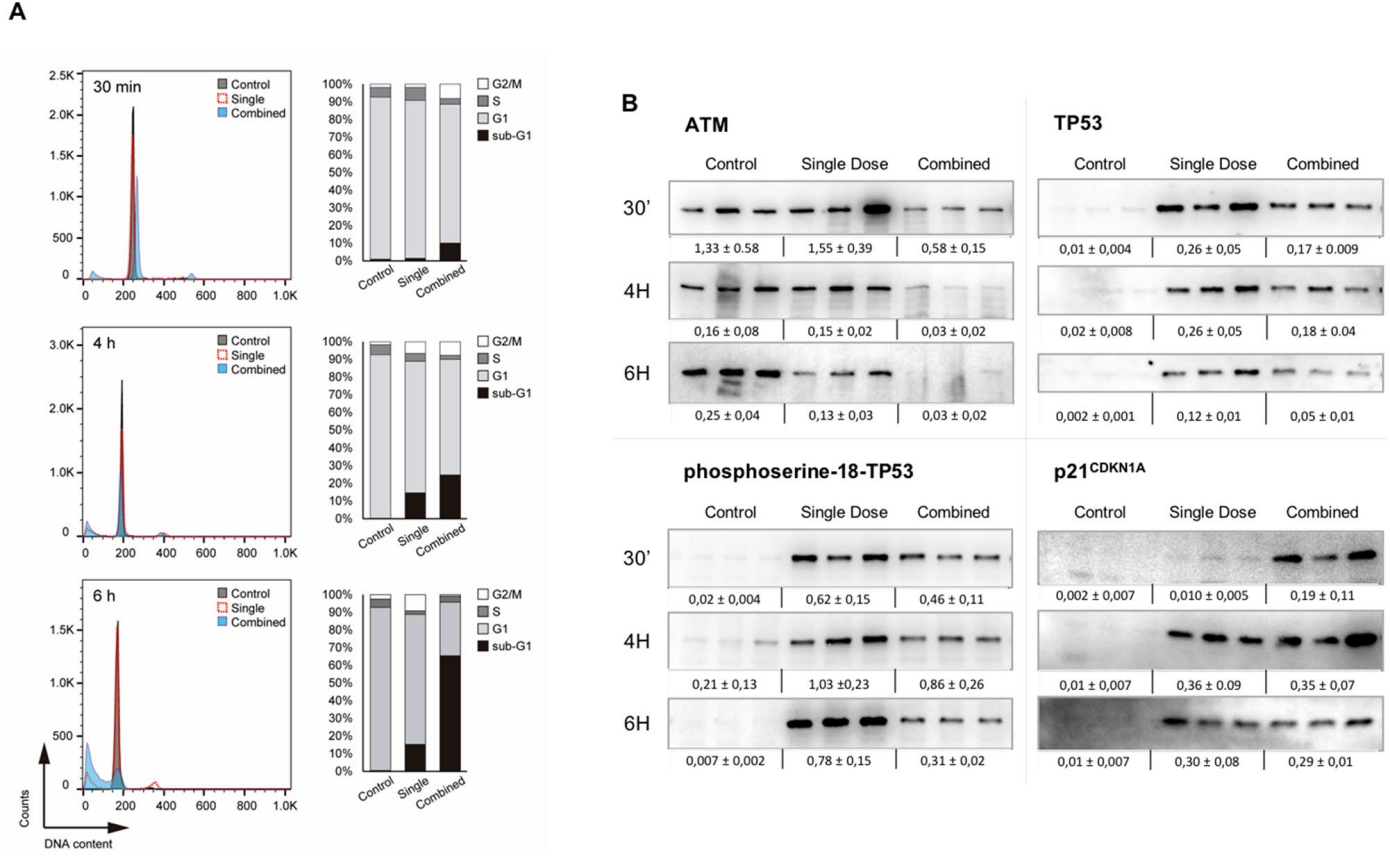
In vivo proliferation study of mouse thymocytes after a single or combined radiation regime. (**A**) Cell cycle analysis using flow cytometry. Propidium iodide (PI) staining of thymocytes for each radiation regime. Representative plot depicted for each time point (left panels) and the mean of 3 biological replicates (right panels). Control cells, single dose (1.75 Gy) and combined regime (0.075 Gy + latency + 1.75 Gy) at the different times assayed after the end of each regime, 30 min (upper panels), 4 h (center panels) and 6 h (lower panels). (**B**) Western blot analysis of several proteins of the ATM-TP53-phosphoserine-18-TP53 axis. Radiation dose-response and time-dependent protein expression of ataxia telangiectasia mutated protein (ATM) (350 kDa); tumor protein p53 (TP53) (53 kDa); phosphoserine-18-TP53 and p21^CDKN1A^ (21 kDa)^.^ Blots show 3 biological replicates for each radiation regime. β-Actin was probed as a loading control for each membrane as shown in annexed Fig. 1. Original blots are presented in supplementary information.

We investigated the effect of administering a combined radiation regime in vivo where a priming radiation dose of 0.075 Gy (X-rays) was delivered prior to challenging the cohort with a higher radiation dose of 1.75 Gy of *γ*-rays (challenging dose) with a 6-h interval between both doses. The outcome was compared to that of a control unirradiated cohort and a single dose cohort (1.75 Gy of *γ*-rays) (Supplementary Fig. 1A). Cell cycle distribution in mouse thymocytes was analyzed at three different times 30 min, 4 h and 6 h after exposure. Thirty minutes after radiation exposure, thymocytes that underwent the combined regime showed a significant accumulation in sub-G1 phase that increased from 1.02 to 11.19%, concomitant with a reduction in the G1 percentage from 91.74 to 77.6% and an S phase reduction from 5.51 to 3.17%, and an increment of cells in the G2/M phase from 1.77 to 7.96% when compared to control thymocytes. At this time point those irradiated with a single challenging dose showed no significant differences with the control cohort (Fig. 1A upper panel and Supplementary Table 3). Four hours after irradiation, thymocytes challenged with the combined regime continued to accumulate in sub-G1 phase (32.93%) together with a further reduction in G1 (58.17%). Those of a single dose alone started to accumulate in sub-G1 increasing from 1.48 to 17.17%. In fact, their distribution at this time point looks similar to that seen in thymocytes which underwent the combined regime 30 min after exposure (Fig. 1A central panel and Supplementary Table 3). This differential cell cycle response between the two regimes was found more pronounced 6 h after exposure where the sub-G1 phase encompasses over 65% of the cells analyzed in thymocytes exposed to the combined regime (Fig. 1A lower panel and Supplementary Table 3) whilst the distribution of thymocytes only irradiated with a single dose did not change from that seen at the 4-h time point (17.87%).

### A differential response of the ATM-TP53-phosphoserine-18-TP53 axis leads to a faster accumulation of p21^CDKN1A^ in thymocytes exposed to the combined regime

We investigated the impact of the ATM/TP53-phosphoserine-18-TP53/p21^CDKN1A^ axis in cell cycle arrest after the combined treatment. We measured the protein amounts in thymocytes exposed to either radiation regimen. Thirty minutes after exposure ATM expression in thymocytes exposed to the combined regime shows a modest reduction that increases with time. In fact, the expression of this protein was almost abolished 6 h after the combined regime exclusively (***p ≤ 0.001). Six hours after exposure to the single dose alone we found a decrease in ATM amounts but far from those levels seen in the combined regime (*p ≤ 0.05) (Fig. 1B). TP53 and its phosphorylated form in the serine 18 where both induced by radiation in the combined and single regime, however less induction and a faster decay was observed in the combined regime when compared to the single dose alone (Fig. 1B and Supplementary Table 4). The expression profiling of p21^CDKN1A^ was different in thymocytes exposed to a combined regime when compared to the single dose. Thirty minutes post-irradiation the thymocytes which underwent a combined regime showed p21^CDKN1A^ induction (*p ≤ 0.05) whilst the single dose did not accumulate comparable amounts of this protein until 4 h after exposure (Fig. 1B and Supplementary Table 4).

### Damage detection and cell death pathway activation occur faster in thymocytes exposed to a combined regime

**Figure 2:**
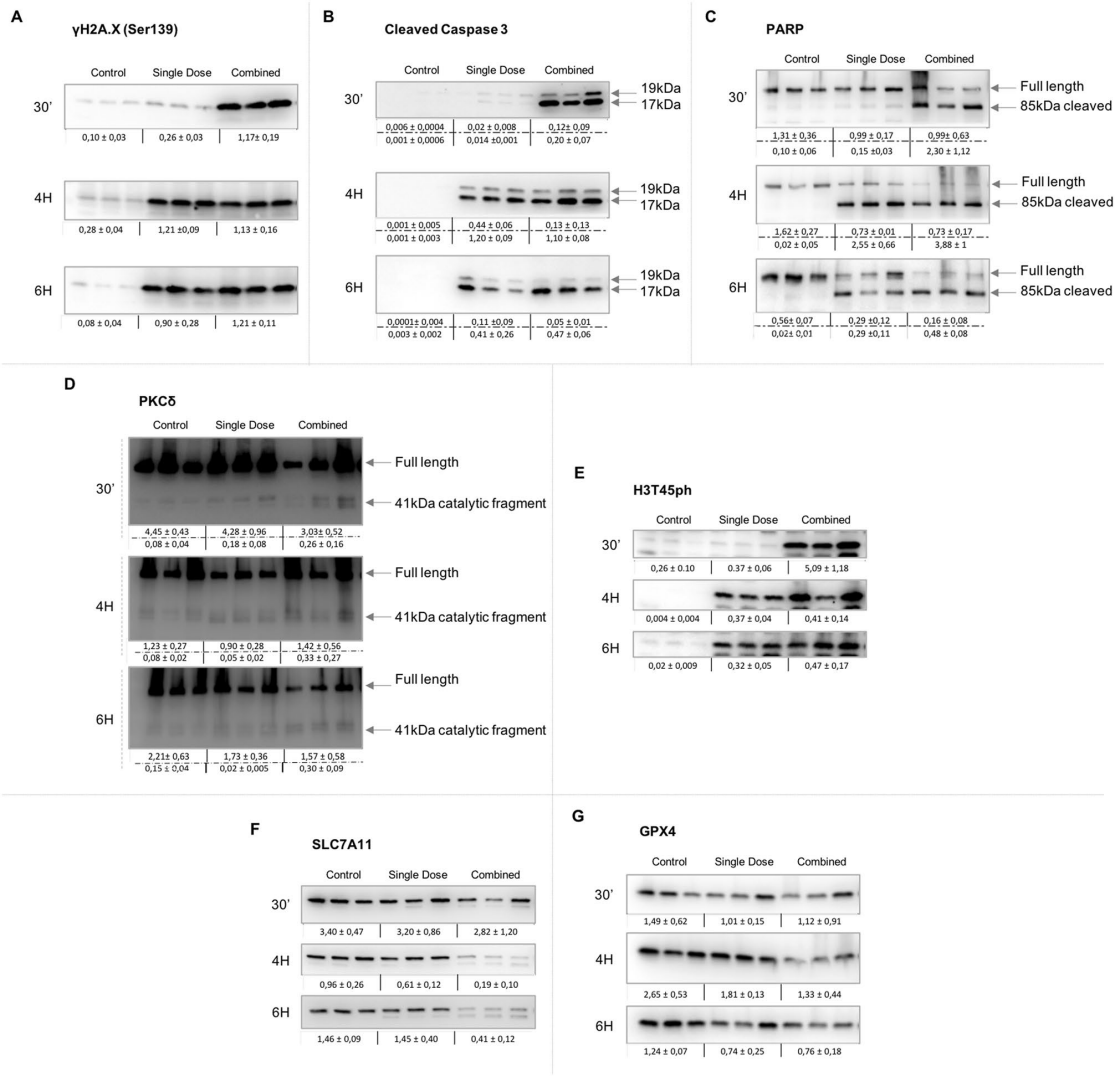
Study of the DNA damage response and cell death pathways in thymocytes in vivo after a single or combined radiation scheme. Time and dose dependent accumulation of DNA damage and apoptotic pathways markers as determined 30 min, 4 and 6 h after irradiation in control and irradiated thymocytes (with single and combine schemes) by immunoblotting. Damage response marker: (**A**) γH2AX-Ser139 (15 kDa). Apoptosis response markers: (**B**) Caspase 3 activated subunits (19 and 17 kDa). (**C**) Poly (ADP-ribose) polymerase-1 (PARP1) full length (116 kDa) and its cleavaged form (89 kDa fragment). (**D**) PKCδ full length (78 kDa) and its catalytic fragment (41 kDa). (**E**) Phosphorylation of H3T45 (H3T45ph) (15 kDa). Ferroptosis response markers: (**F**) xCT (55 kDa) and (**G**) GPX4 (17 kDa). β-Actin was probed as a loading control for each membrane in annexed Fig. 2. Original blots are presented in supplementary information.

Our analysis revealed an exclusive overexpression of *γ*H2AX in radio-adapted thymocytes 30 min after irradiation (****p ≤ 0.0001), and a uniform accumulation of this protein 4 and 6 h after any of the radiation treatments (Fig. 2A and Supplementary Table 4). Hinted by the accumulation of cells in sub-G1 seen in Fig. 1A, we evaluated the potential role of caspase-3 activation at inducing cell death associated with the combined regime. Strong expression of Cleaved Caspase-3, both p17 and p19 subunits, were found only 30 min post-irradiation with such increase sustained over time in thymocytes exposed to the combined regime. Accumulation of p19 and p17 subunits of cleaved caspase-3 in single dose exposed thymocytes was first detectable 4 h after radiation exposure (**p ≤ 0.01 and ****p ≤ 0.0001 respectively) and maintained its constant expression 6 h post-irradiation (Fig. 2B and Supplementary Table 4).

We sought to study how the activation of caspase-3 seen in thymocytes exposed to the combined dose might affect the proteolysis of PARP1 and PKCδ as well as PKCδ-mediated histone 3 phosphorylation at threonine 45 (H3T45). Protein profiling of the 85 kDa PARP1 fragment (Fig. 2C), the 41 kDa catalytic fragment of PKCδ (Fig. 2D) and H3T45 who is phosphorylated by PKCδ (Fig. 2E) were similar to those found for Cleaved Caspase-3 in thymocytes exposed to a combined dose of radiation (Supplementary Table 4). These results show an earlier activation of the caspase-3-mediated apoptotic signaling pathway in the combined regime when compared to those exposed to a single dose of radiation.

### Exclusive activation of ferroptosis in thymocytes exposed to the combined radiation scheme

Our study did not reveal significant differences in the expression of cleaved caspase-3 and its downstream targets between radiation treatments at 4 h and 6 h post-irradiation (Supplementary Table 4). However, thymocytes exposed to the combined regime showed an increased percentage of cells in the sub-G1 fraction at those times assayed (Fig. 1A center and lower panels) suggesting that other cell death signaling pathways could also play a role in thymocytes exposed to the adaptive regimen of IR. We studied whether ferroptosis could be an additional cell death mechanism being activated by the combined dose. To demonstrate this hypothesis, we measured the protein amounts of ferroptosis inhibitors xCT and GPX4 in thymocytes exposed to a combined and non-combined regime of radiation. Our data demonstrates a slight reduction of xCT protein in thymocytes exposed to a combined regime 30 min after exposure that becomes most pronounced 4–6 h after exposure exclusively in the combined scheme cohort **p ≤ 0.01 (Fig. 2F) correlating with those times of increasing sub-G1 fraction (Fig. 1A). This correlates with a modest reduced expression of GPX4, *p ≤ 0.05, 4 h after exposure (Fig. 2G) suggesting that the combined regime induces thymocyte ferroptosis through the reduction of the xCT pathway.

### The epigenetic profile of thymocytes exposed to the combined regime showed changes on chromatin remodellers key to the damage response activation

**Figure 3:**
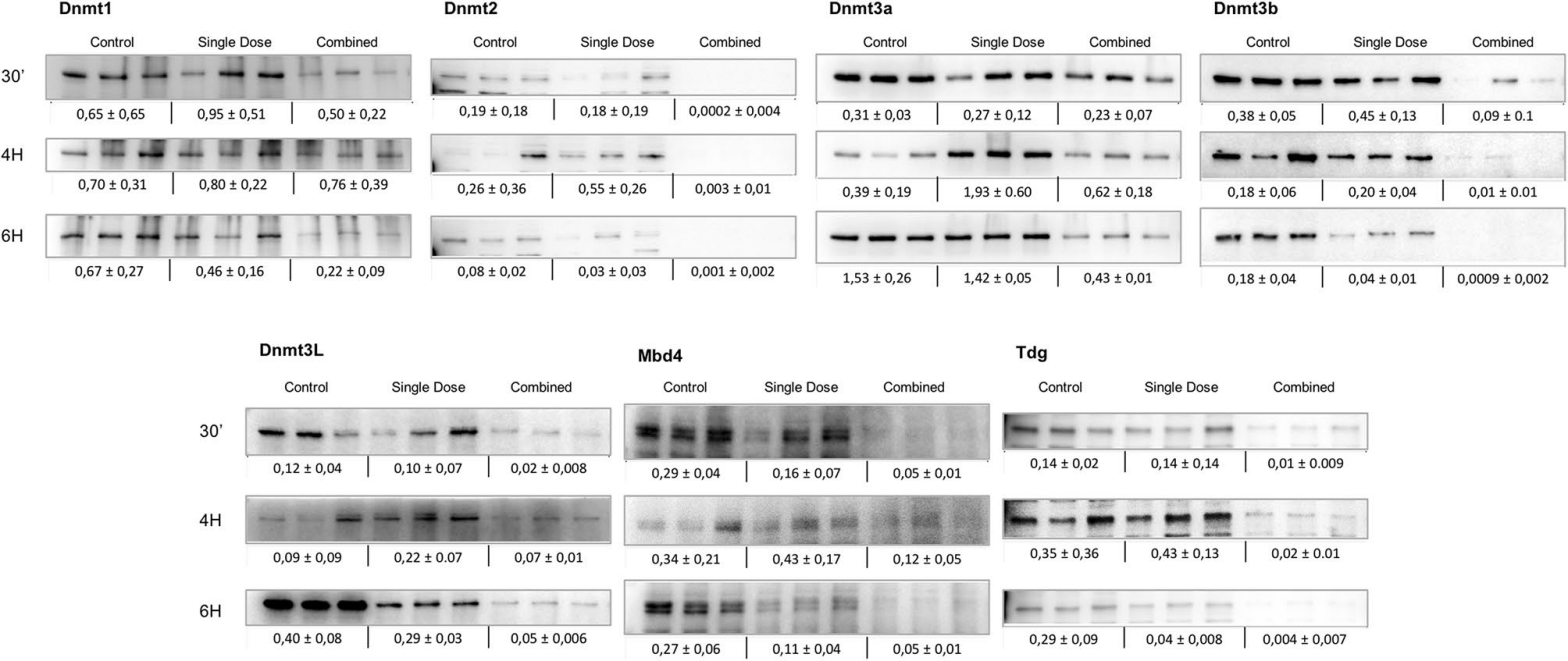
Study of the response of chromatin remodelers to a combined radiation regime versus control or single irradiated mouse thymocytes in vivo. Western blots of 3 biological replicates showing time-dependent changes (30 min, 4 and 6 h) in the expression of several chromatin remodelers such as DNA methylases: DNMT1 (200 kDa), DNMT2 (55 kDa), DNMT3A (130 kDa), DNMT3B (96 kDa) and DNMT3L (49 kDa) and glycosylases such as MBD4 (Methyl Binding Domain) (60–65 kDa) and TDG (Thymine DNA glycosylase) (55–65 kDa) in control thymocytes, thymocytes exposed to a single dose (1.75 Gy) or thymocytes exposed to a radiation combined regime (0.075 Gy + latency + 1.75 Gy). β-Actin was probed as a loading control for each membrane in annexed Fig. 3 Original blots are presented in supplementary information.

The study of a panel of DNMTs and glycosylases known to play a crucial role at chromatin remodelling was performed. 30 min after exposure, thymocytes exposed to the combined regime showed a reduction of the DNA methyltransferases DNMT1, DNMT2, DNMT3B, DNMT3L and the glycosylases MBD4 and TDG (Fig. 3) which was sustained in all the times assayed when compared to both, the control animals and those irradiated with a single dose alone, which only managed to reduce the expression of some of these chromatin modifiers/remodellers (DNMT2, DNMT3B, DNMT3L, MBD4 and TDG) 6 h after exposure. DNMT3A showed an expression reduction only 6 h after exposure (***p ≤ 0.001) to the combined regime, whilst not varying much over time in thymocytes exposed to the single dose alone.

### The study of the underlying cellular status six hours after the priming dose alone showed evidence of retained modifications on several molecular pathways

**Figure 4:**
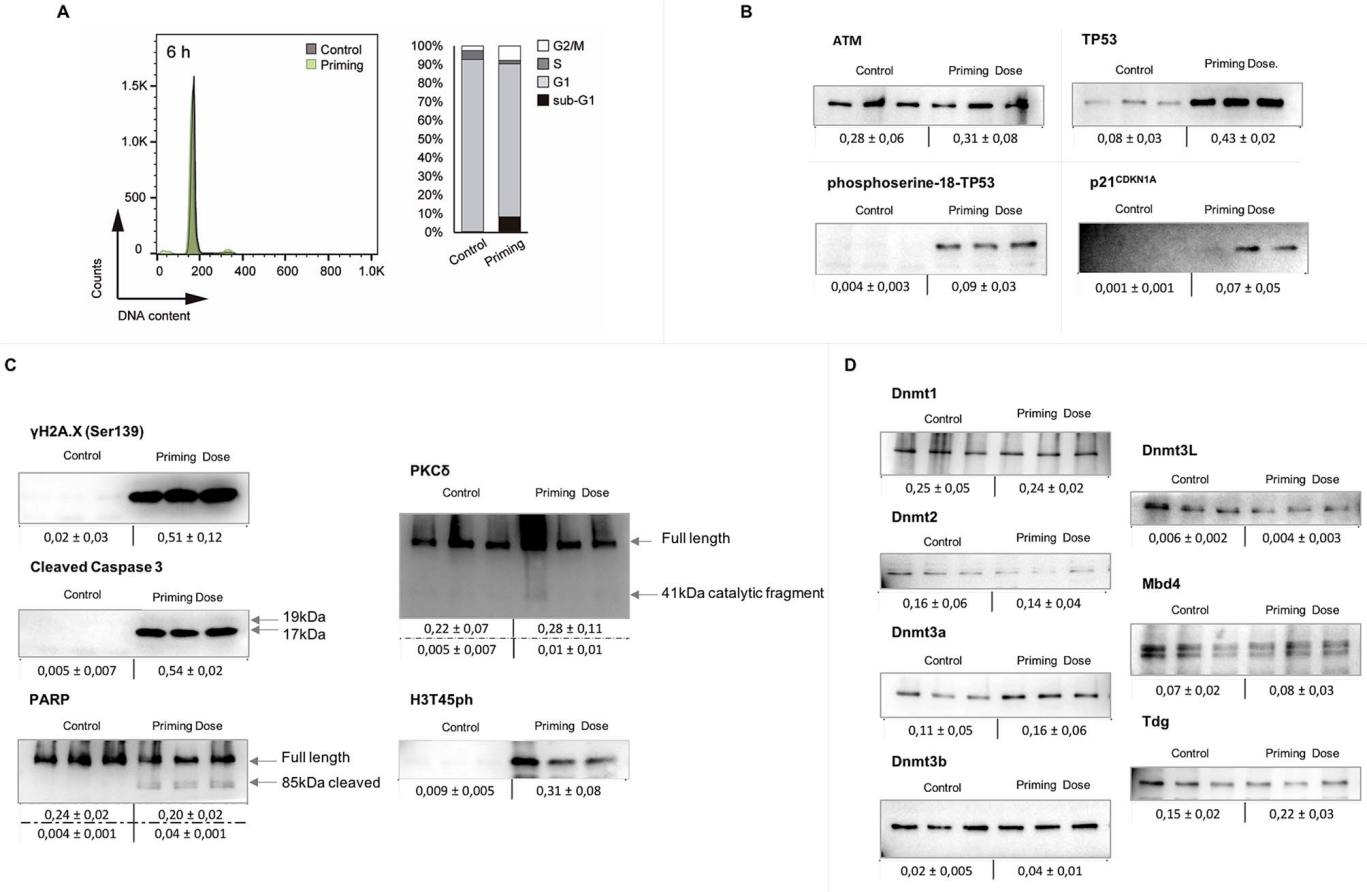
Study of the remnant radiation response 6 h after exposure to the priming dose (0.0075 Gy) in mice in vivo. (**A**) Cell cycle analysis using flow cytometry. Propidium iodide (PI) staining of thymocytes for control cells and thymocytes exposed to the priming dose (0.0075 Gy) 6 h after exposure. Representative plot (left panel) and the mean of 3 biological replicates (right panel). (**B**) Western blot analysis of several proteins of the ATM-TP53-phosphoserine-18-TP53 axis. Expression changes of ataxia telangiectasia mutated protein (ATM); tumor protein p53 (TP53); phosphoserine-18-TP53 and p21^CDKN1A^. Blots show 3 biological replicates for each radiation regime. (**C**) Markers of the damage response and cell death pathways were determined by immunoblotting with antibodies specific against γH2AX-Ser139 (15 kDa), Caspase 3 activated subunits (19 and 17 kDa), Poly (ADP-ribose) polymerase-1 (PARP1) full length (116 kDa) and its cleavaged form (89 kDa fragment), PKCδ full length (78 kDa) and its catalytic fragment (41 kDa) and Phosphorylation of H3T45 (H3T45ph) (15 kDa). (**D**) Chromatin remodellers study 6 h after the priming dose. Comparative analysis of the endogenous expression of DNMTs DNMT1 (200 kDa), DNMT2 (55 kDa), DNMT3A (130 kDa), DNMT3B (96 kDa) and DNMT3L (49 kDa) and the glycosylases MBD4 (Methyl Binding Domain) (60–65 kDa) and TDG (Thymine DNA glycosylase) (55–65 kDa) in mouse thymocytes 6 h after the priming dose. β-Actin was probed as a loading control for each membrane in annexed Fig. 4. Original blots are presented in supplementary information.

We aimed to understand the underlying cellular status that could give rise to a differential response to the challenging dose in a combined regime when compared to the single dose alone. For this purpose, we irradiated mice with 0.075 Gy and compared them to their unirradiated counterparts 6 h after radiation exposure. The assays were performed on the same pathways found differentially regulated after the combined regime study. Thymocytes extracted from irradiated mice with the priming dose alone showed differences in the cell cycle distribution after the latency period of 6 h (Fig. 4A). The sub-G1 phase increased from 0.7 to 8.5%, and the S phase decreased from 4.72 to 1.86% and also G2/M phase changed from 2.51 in controls to 7.70% 6 h after the priming dose (Supplementary Table 5).

Regarding the ATM/TP53/phosphoserine-18-TP53 axis, whilst ATM shows no significant differences at the given time point, TP53 remains upregulated 6 h after the priming dose alone (**** *p* ≤ 0.0001), the same as the p18Ser-TP53 (**p ≤ 0.01) and p21^CDKN1A^ (*p ≤ 0.05) (Fig. 4B). Strikingly, the principal effector of signalling DNA damage *γ*H2AX remains upregulated in those cells treated with the priming dose alone (***p ≤ 0.001) (Fig. 4C). Moreover, the cell death pathway activation via Caspase-3 cleavage to generate the fully mature p17 form of the enzyme (****p ≤ 0.0001), subsequent PARP1 proteolysis (****p ≤ 0.0001) and PKCδ-dependent H3T45 phosphorylation (***p ≤ 0.001) seem to be activated in those cells as well (Fig. 4C). It is important to note that, at 6 h after the priming dose alone we observed no alterations in xCT/GPX4 protein levels (Supplementary Fig. 2).

To further understand on the role of epigenetic mechanisms on the response to the combined regime of radiation, we studied the status of several epigenetic markers after the priming dose and the latency interval. Firstly, we measured the global DNA methylation levels of the animals irradiated with the priming dose versus the unirradiated controls to find a global DNA hypomethylation after the latency period of 6 h as expected (Supplementary Fig. 3A). A targeted DNA methylation study of the promoters of those genes assayed in this work showed a specific hypomethylation of all promoter regions except for *Parp1* and *Pkc*δ in thymocytes exposed to the combined radiation scheme (Supplementary Fig. 3B). Regarding chromatin remodellers however, the priming dose alone followed by the latency period of 6 h showed no persistent changes on any of the remodellers later found altered in the combined regime.

### Human foetal thymocytes show a similar response to the combined regime as that previously seen in mice

**Figure 5:**
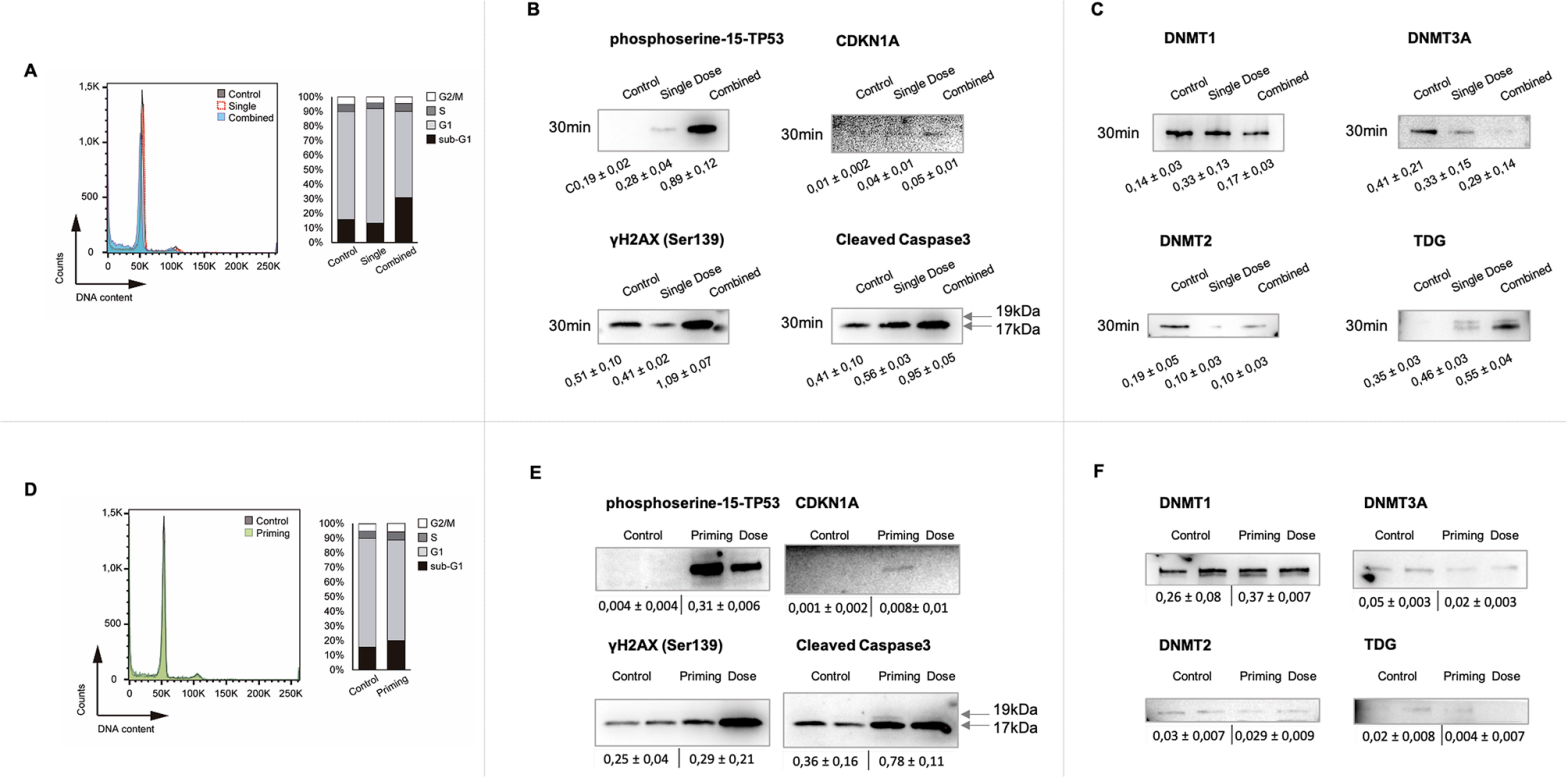
Ex vivo response to a combined regime in human thymocytes. (**A**) Cell cycle analysis using flow cytometry of human thymocytes exposed to radiation ex-vivo. Propidium iodide (PI) staining of thymocytes for each radiation regime; control cells, single dose (1.75 Gy) and combined regime (0.075 Gy + latency + 1.75 Gy) 30 min after exposure. Representative plot depicted in the left panel. The mean of 3 biological replicates is shown in the right panel. (**B**). Western blot analysis of several proteins of the ATM-TP53-phosphoserine-18-TP53 axis, damage response and cell death pathways in human thymocytes. Expression changes of phosphoserine-15-TP53, p21^CDKN1A^, γH2A.X-Ser139 (15 kDa) and caspase 3 activated subunits (19 and 17 kDa). Blots show 1 representative biological replicate. (**C**) Chromatin remodellers study in human thymocytes. Protein profiling of DNMT1 (200 kDa), DNMT2 (55 kDa), DNMT3A (130 kDa) and TDG (55–65 kDa) after the radiation treatment with either a single dose (1.75 Gy) or the combined regime (0.075 Gy + latency + 1.75 Gy) 30 min after exposure. Representative blots are presented. (**E**) Priming dose study in human thymocytes. Protein status of phosphoserine-15-TP53, p21^CDKN1A^, γH2AX-Ser139 (15 kDa) and caspase 3 activated subunits (19 and 17 kDa) 6 h after the priming dose (0.0075 Gy). (**F**). Chromatin remodellers study 6 h after the priming dose in human thymocytes. Protein expression study of DNMT1, DNMT2, DNMT3A and TDG. Data shows 2 biological replicates for the control cells and 2 biological replicates for cells irradiated with the priming dose after a latency of 6 h. β-Actin was probed as a loading control for each membrane in annexed Fig. 5. Original blots are presented in supplementary information.

Cell cycle analysis of ex vivo human thymocytes 30 min after exposure to either a single dose or combined regime reveals an exclusive accumulation in sub-G1 of human thymocytes exposed to the combined radiation scheme (Fig. 5A and Supplementary Table 6) matching the results previously seen in mouse in vivo experiments.

The study of the ATM/TP53/phosphoserine-15-TP53/p21^CDKN1A^ axis showed an increase of the phosphoserine-15-TP53 and CDKN1A proteins in thymocytes exposed to the combined regime when compared to both control cells (***p ≤ 0.001 and ****p ≤ 0.0001) and single dose (***p ≤ 0.001 and **p ≤ 0.01) regimes 30 min after exposure (Fig. 5B). The proteins ATM, p53 and the phosphorylated form of H3T45 were not found dysregulated in these cells at this time point (Supplementary Fig. 4A). Regarding damage signaling, these cells show an expression of *γ*H2AX already in control cells (probably due to handling and the nature of the explants) but the protein levels were found increased exclusively in those thymocytes exposed to the combined regime as early as 30 min after exposure (****p ≤ 0.0001). The same behavior was found for caspase-3 (*p ≤ 0.05) and PARP1 (*p ≤ 0.05 full length and ***p ≤ 0.001 cleaved form), when compared to control cells. Please note the activation of these proteins in control cells potentially due to the handling of these ex-vivo samples and the thawing procedures^18^ (Fig. 4C). In the case of xCT, no difference was observed 30 min after exposure (Supplementary Fig. 4B).

The epigenetic study performed on mouse epigenetic modifiers and chromatin remodellers was mimicked in the 3 ex vivo human foetal thymocytes to find out that DNMT2 and DNMT3A was reduced in both the combined and single dose cohorts (****p ≤ 0.0001) (Fig. 4C). In the case of the glycosylases included in the study we found TDG upregulated, ****p ≤ 0.0001, 30 min after exposure to the combined regime opposite to the response seen in mice. In the case of the single dose, we found an increase (***p ≤ 0.001) in the amounts of this glycosylase (Fig. 5C).

The study of the cellular status 6 h after the priming dose in humans revealed that the sub-G1 fraction was increased 6 h after the exposure to the 0.075 Gy priming dose (Fig. 5D). Regarding the protein study after the priming dose, we found phosphoserine-18-TP53 upregulated (****p ≤ 0.0001) (Fig. 5E) in 2 biological replicates. p21^CDKN1A^ and *γ*H2AX show non-statistical differences, however cleaved Caspase-3 p17 subunit shows significant expression differences (**p ≤ 0.01) (Fig. 5E). Regarding chromatin remodellers, human thymocytes exposed to the priming dose alone showed no significant changes on the chromatin remodellers, except for a significant reduction of DNMT3A expression (****p ≤ 0.0001) and TDG (*p ≤ 0.05) (Fig. 5F).

## Discussion

Radiation therapy is key to treating certain human diseases, and it is of great importance to find possible alternatives more effective at tumour killing and healthy tissue recovery. The combined regime where a low priming dose is given prior to a subsequent challenging dose reduces the risk of late adverse effects of ionizing radiation exposures^19–23^. Such primed treatments have been lately in the spotlight towards triggering anti-tumour responses^24–26^. Understanding the effects of a priming radiation dose is also essential to understand abscopal radiation responses^27^. However, little is known about the molecular mechanisms behind the effect of a priming dose in a combined regime at the cellular level. Here we provide evidence that the response to a combined radiation dose is associated with a faster, more efficient cell cycle arrest and a rapid activation of cell damage response as well as a differential induction of cell death pathways in in vivo irradiated mouse thymocytes. This response seems to be reiterated by human thymocytes assayed ex vivo. Mouse and human thymocytes irradiated with a combined regime blocked the cell cycle and accumulated in sub-G1 as early as 30 min after exposure to the combined regime exclusively to further increased this accumulation over time.

Moreover, we found retained cell cycle alterations (including sub-G1 accumulation) 6 h after the priming dose alone, a baseline inferred by the priming dose to later be enhanced by the full combined regime. Other authors have shown that G2-phase arrest and an upregulation of cell cycle control pathways are detectable at low doses^28^, appointing the so called low dose hypersensitivity^29^ that seems key at explaining the response to the combined regime seen in this study*.* The priming dose sensitizes cells to radiation, inferring an “awareness phenotype” to the thymocytes that can more efficiently respond to the subsequent challenging dose in the combined regime.

Combined radiation scheme exposed thymocytes reduced ATM expression in a time dependent manner. Previous data reported that the decrease in ATM levels caused failure to obtain a p53 / p21^CDKN1A^ response and did not impose a stop at the G1 checkpoint after DNA damage^30–32^. In this study, such reduction did not avoid TP53 accumulation nor its phosphorylated form, suggesting a differential activation of this pathway by the combined regime. One must bear in mind however that the ATM induction could have happened earlier than 30 min after exposure in the combined cohort. In fact, only in the combined exposed thymocytes cohort, early transcriptional induction of p21^CDKN1A^ was found (30 min after exposure). The accumulation of p21^CDKN1A^ was not visible in thymocytes exposed to a single dose regime until 4 h after exposure demonstrating a faster response of the ATM/TP53/ p21^CDKN1A^ axis in mice hit with the combined-dose regime. When assessing the cellular background 6 h after the priming dose alone, we discovered no changes in ATM expression at that time point (which again does not discard an earlier activation of the protein) but rather increase in cellular amounts of its downstream effectors, TP53, phosphorylated-18-TP53 and p21^CDKN1A^. This suggests that the priming dose activates this pathway and the cells retain enough increased protein levels during the latency period as to having them available to faster and more efficient activation of the pathway after the challenging dose.

The damage recognition pathway represented by gamma *γ*H2AX^33^ was induced by the priming dose. This made the γH2AX response to the challenging dose within a combined scheme faster both in mice and human thymocytes. In addition, *γ*H2AX is not required for Caspase-3 activation but both cooperate to determine the final cellular fate^34^. Our analysis for Cleaved Caspase-3 demonstrates the presence of both p19 and p17 Caspase-3 subunits as early as 30 min after combined regime of irradiation in mouse thymocytes. It is known that Cleaved Caspase-3 propagates an apoptotic signal inducing proteolysis of several enzymes. Among them, the DNA repair enzyme including poly (ADP-ribose) polymerase 1 (PARP1)^35^ and the protein kinase C delta (PKCδ)^36^. PARP1 is specifically proteolysed by the p17 Caspase-3 subunit to a 24 kDa DNA-binding domain (DBD) and to a 89 kDa catalytic fragment during the execution of the apoptotic program^37,38^. PKCδ is a cytoplasmic p19 caspase-3 substrate resulting in the production of a 41 kDa constitutively proapoptotic active catalytic fragment (CFδ)^39–41^. In this study we found activation of caspase 3 leading to the processing of PARP1 in response to the combined regime as early as 30 min after exposure when compared to the single dose alone in mouse thymocytes. The study of the cellular status prior to the challenging dose revealed an exclusive accumulation of the p17 subunit of caspase3 and its downstream effector PARP1 induced by the priming dose. A comparable pathway activation is only found in single dose thymocytes 4 h after exposure demonstrating the effectiveness of a combined regime at inducing cell killing. These data demonstrate that the PARP1 pathway activation by the priming dose alone is crucial for the differential apoptotic induction found in the combined regime. It might, in fact, be a decisive event during the progression to cellular demise as other authors reported in different experimental models^37,38^. In human thymocytes however, caspase activation was specific to the subunit p17 both after the priming dose and the combined regime concomitant with a modest increase in PARP1 backing up the mouse data on the importance of the cleavage of this specific subunit of caspase 3 in the radiation response to the combined radiation regime.

In the case of PKCδ, its cleavage (mediated by the p19 subunit of Caspase-3) seems to be triggered by the challenging dose independently of whether it is a single or a combined dose cohort in mouse thymocytes. However, it is proteolysed from the full length to the catalytic fragment faster in the combined regime. Hurd et al*.* demonstrated that phosphorylation of H3T45 (H3T45ph), the downstream effector of PKCδ increases dramatically in apoptotic cells, around the time of DNA nicking and/or fragmentation^42^. The positive association of these two molecules is clear in our studies and the accumulation of H3T45ph is exclusive of the combined dose cohort 30 min after exposure. The altered chromatin structure favors DNA processing in late apoptosis, such as DNA nicking and/or fragmentation, explaining the abundance of the mark at this time when we subject animals to a combined regimen of irradiation. Strikingly, in the study of the effects of the priming dose alone, we find this pathway already activated as Cleaved Caspase-3, cleaved PARP1, PKCδ and phosphorylated H3T45 appear highly expressed in these cells 6 h after the priming dose alone. Following after the TP53 axis response, this pathway seems to be activated contributing at sensitization by the priming dose, allowing thymocytes to faster respond to the subsequent challenging dose in mice. In human thymocytes however, p19 and the PKCδ pathway does not seem to contribute to the response in the times assayed.

There was a differential sub-G1 accumulation 4 and 6 h after the treatment between the combined and the single dose cohorts not explained by the canonical apoptotic pathway. Therefore, we wondered if other cell death mechanisms could be differentially induced in the combined regime. Ferroptosis, an iron-related cell death induced by excessive lipid peroxidation^43^, is known to be activated in IR-induced cell death response^44^. Furthermore, IR leads to glutathione depletion through the inhibition of xCT (also known as glutamate transporter SLC7A11) and, consequently, inactivates glutathione peroxidase 4 (GPX4), whose function is to remove lipid peroxides^45^. In this study we show the activation of this pathway by depletion of xCT exclusively in the combined regime. However, this pathway seems to need both radiation doses, as the expression of the inhibitor does not change with the priming dose alone and this leads to a later activation of this pathway that would match the cell cycle distribution data. We provide the first evidence that the combined radiation regime promotes ferroptosis. Regarding this pathway, it is known that using xCT-inhibitors increased the efficacy of other therapies^46,47^ or that administration of genotoxic agents to increase DNA damage has been harnessed clinically to improve radiotherapy efficacy and therefore improve the outcomes of patients with cancer. With these data one could suggest the increased efficacy of a combined regime at tumour sensitization regarding ferroptosis induction as well.

Global DNA hypomethylation is a potential biomarker for cancer risk associated with genomic instability, which is an important factor in radiation-induced cancer^48–50^. Most studies showed a global decrease in DNA methylation within a day of radiation exposure^51,52^ therefore it is not surprising that the combined exposure resulted in a significant decrease in global DNA methylation, data that was specifically seen in mice in almost all the promoters of those genes seen altered in this study. These epigenetic changes however were accompanied by decreased levels of DNMT1, DNMT2, DNMT3A, DNMT3B and DNMT3L. This reduction occurs gradually after the full radiation combined regime in mice suggesting the control of different pathways after exposure. Single dose irradiated thymocytes show less efficiency at reducing the expression of these proteins and even in some of them no changes were found. Is it known, for example, that the reduction of DNMT3B sensitized prostate cancer cells to radiation^53^, therefore supporting the theory of sensitized cells by the combined scheme also at an epigenetic level. As previously stated in the introduction, targeted disruption of *Dnmt1* and *Dnmt3a* in cultured cells eliminates the transmission of genomic instability^15^ so one could argue about the preventing potential of the combined regime at buffering the transmission of instability^54^. Furthermore, inhibition of DNMTs has demonstrated reduction in tumour formation, supporting the anti-tumour potential of this combined radiation regime.

Furthermore, the reduction of the glycosylase MBD4 has been functionally linked to apoptosis and plays a role in the effective response to a variety of chemotherapeutic and other cytotoxic drugs^55,56^ and as such, the reduction reported here might be key to the induction of cell death pathways. It was previously reported that TDG knockdown strongly suppresses the tumour-forming capabilities of melanoma suggesting that one or more of the TDG activities are critical for tumour induction, maintenance, and progression^57^ and as shown here, it is also key to the way thymocytes respond to the combined regime which supports the theory of the anti-tumour effectiveness of the combined scheme. All these results were corroborated in human, where the combined radiation regime induced changes in the expression of epigenetic markers although differential regulation levels might be present in human cells.

All these results revealed here could be key responses behind cellular mechanisms such as radio-adaptation by low radiation doses leading to tumour control, as shown by others^25,26^. Therefore, these findings could be of great value at shaping future radiation treatments by enhancing radiation power with small radiation doses rather than using other aggressive alternatives such as the chemo or immunotherapy, as used nowadays, with the same idea of increased sensitization of tumour cells and healthy tissue recovery, in this case led by the hormesis^11^ known to be induced by the low dose (priming dose) triggering a differential, more effective response in the combined regime.

## Supplementary Information

Supplementary Information.
