# Supplementary material for: Differential molecular response in mice and human thymocytes exposed to a combined-dose radiation regime
Source: Sci Rep. 2022 Feb 24;12:3144. doi: 10.1038/s41598-022-07166-8 (PMC8873405; doi:10.1038/s41598-022-07166-8)
Supplement: Supplementary file 1 — Supplementary Information. [file 41598_2022_7166_MOESM1_ESM.pdf]

Supplementary Information

Differential molecular response in mice and human thymocytes exposed to a combined-dose radiation regime.

Pilar López-Nieva[1,2,3,], Iria González-Vasconcellos[1,2,]*, Laura González-Sánchez[1,3,4], María A. Cobos-Fernández[1,2], Sara Ruiz-García[1,2], Raúl Sánchez Pérez[5], Ángel Aroca[5], José Fernández-Piqueras[1,2,3], Javier Santos[1,2,3]*

[1]Genome Dynamics and Function Program. Genome Decoding Unit. Severo Ochoa Molecular Biology Center.

[2]Department of Biology. Madrid Autonomous University. 28049 Madrid, Spain.

[3]Institute of Health Research. Jiménez Díaz Foundation 28040, Madrid, Spain.

[4] Consorcio de Investigación Biomédica de Enfermedades Raras (CIBERER), Madrid, Spain.

[5] Department of Congenital Cardiac Surgery, Hospital Universitario La Paz, Madrid 28046, Spain.

PLN and IGV contributed equally to this work

* Corresponding authors. Phone: +34-911964653 Fax: +34-911964420; Email: (pilar.lopez@cbm.csic.es / iria.gonzalez@cbm.csic.es)

ORCID codes: 0000-0001-6369-2444 to PLN, 0000-0002-4688-5508 to IGV, 0000-0002-4749-2423 to LGS, 0000-0002-8627-076X to SRG, 0000-0003-4520-6785 to JFP and 0000-0002-4168-6251 to JS.

Annexed Figure 1

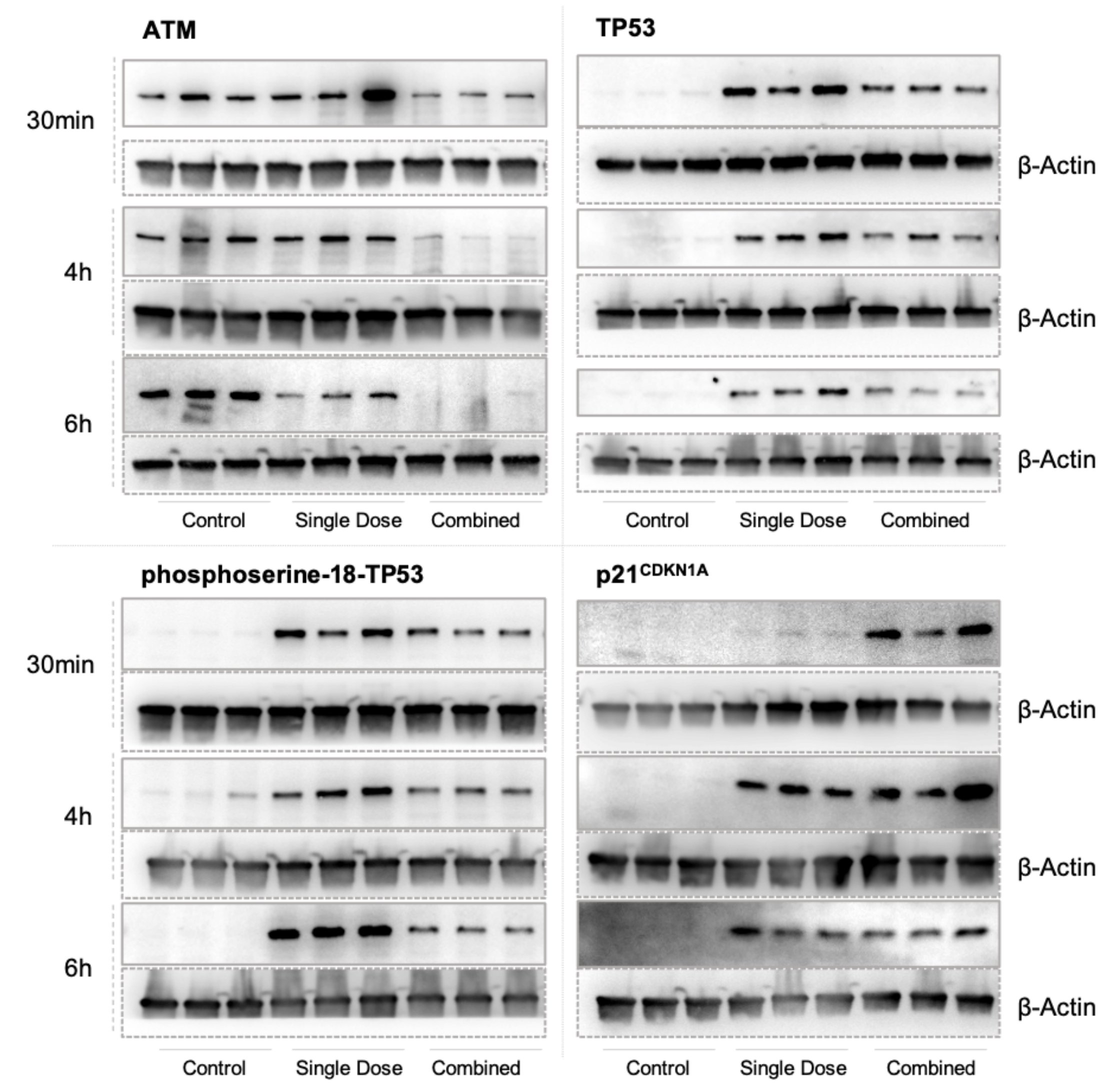

Annexed Figure 2

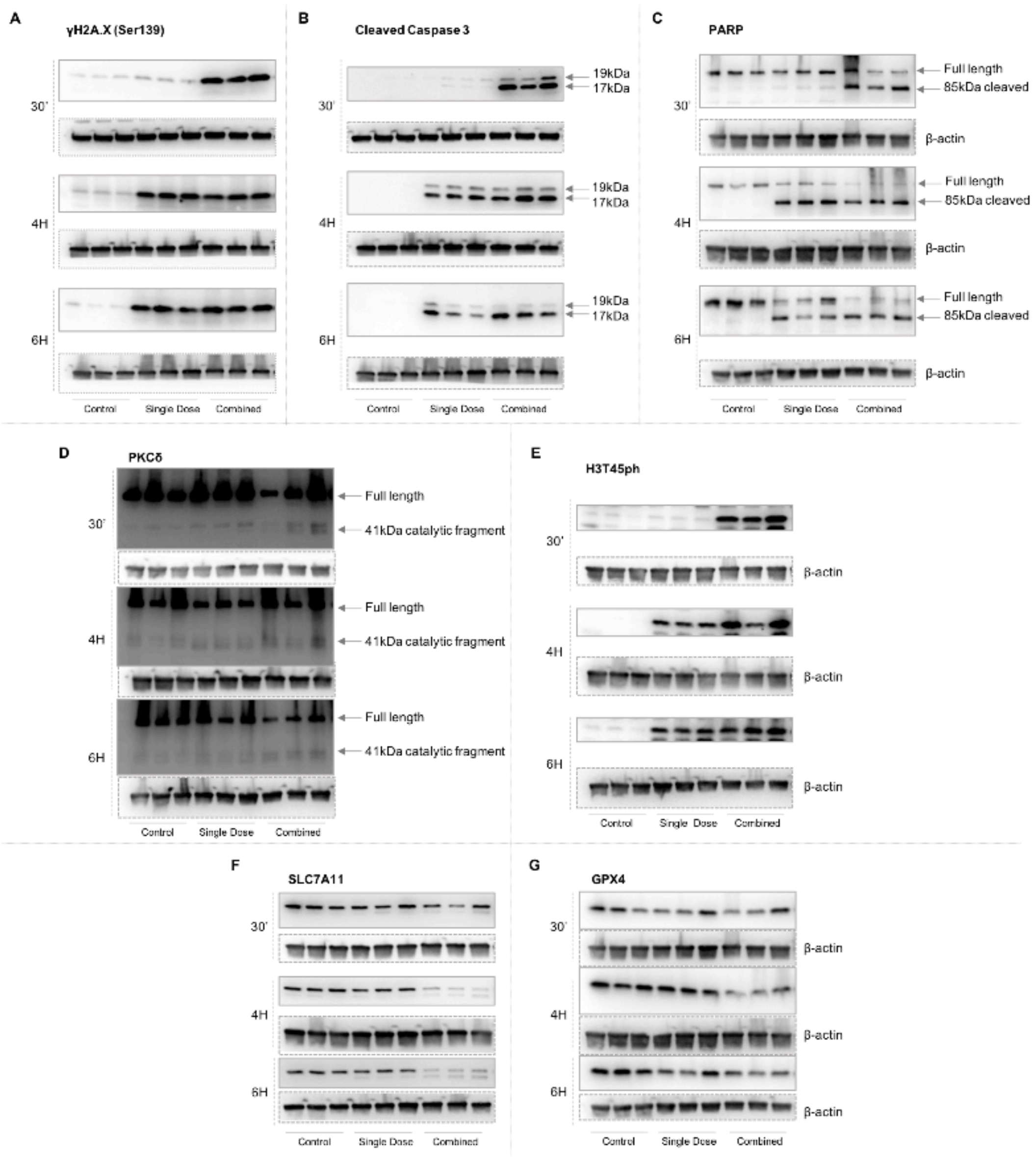

Annexed Figure 3

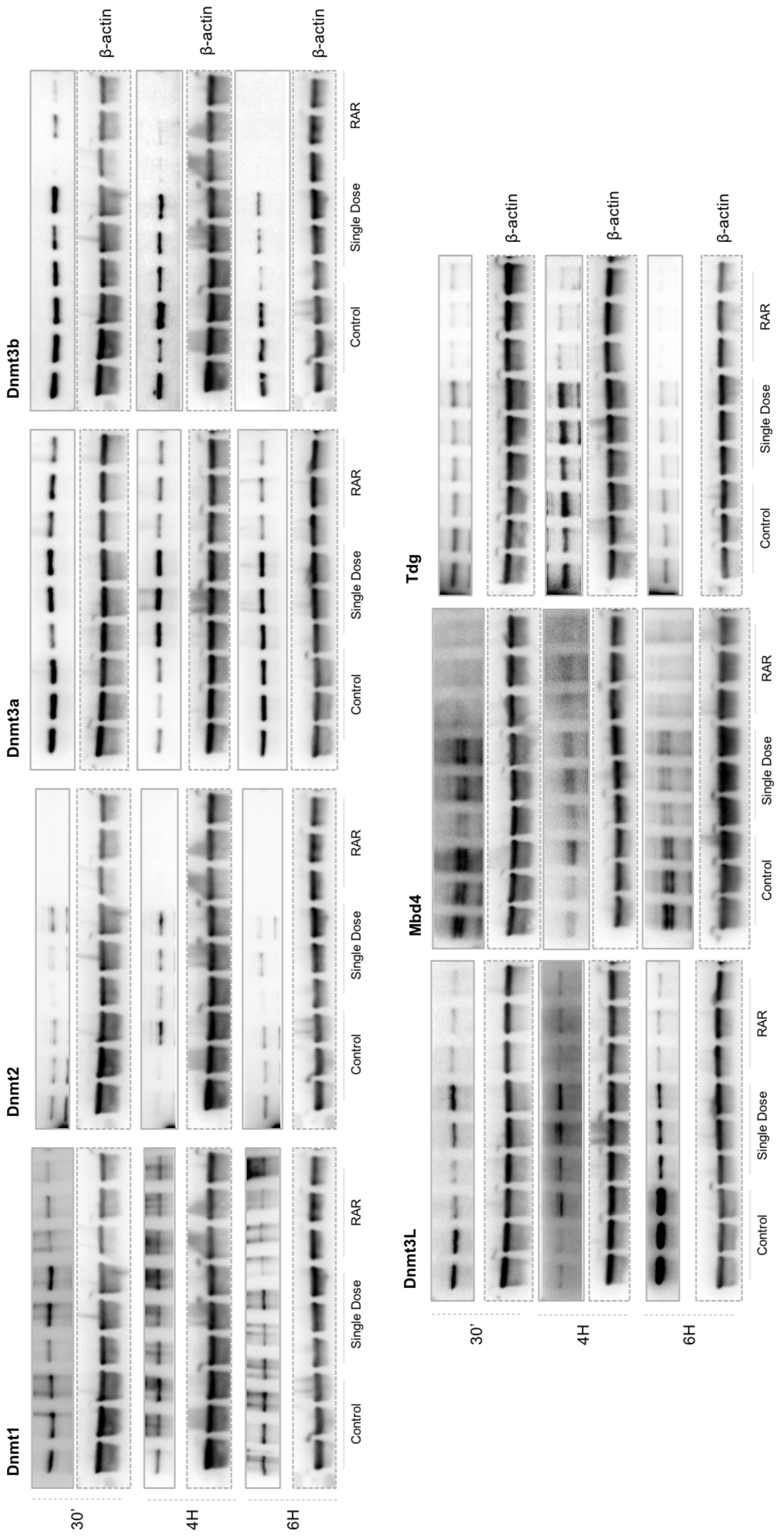

Annexed Figure 4

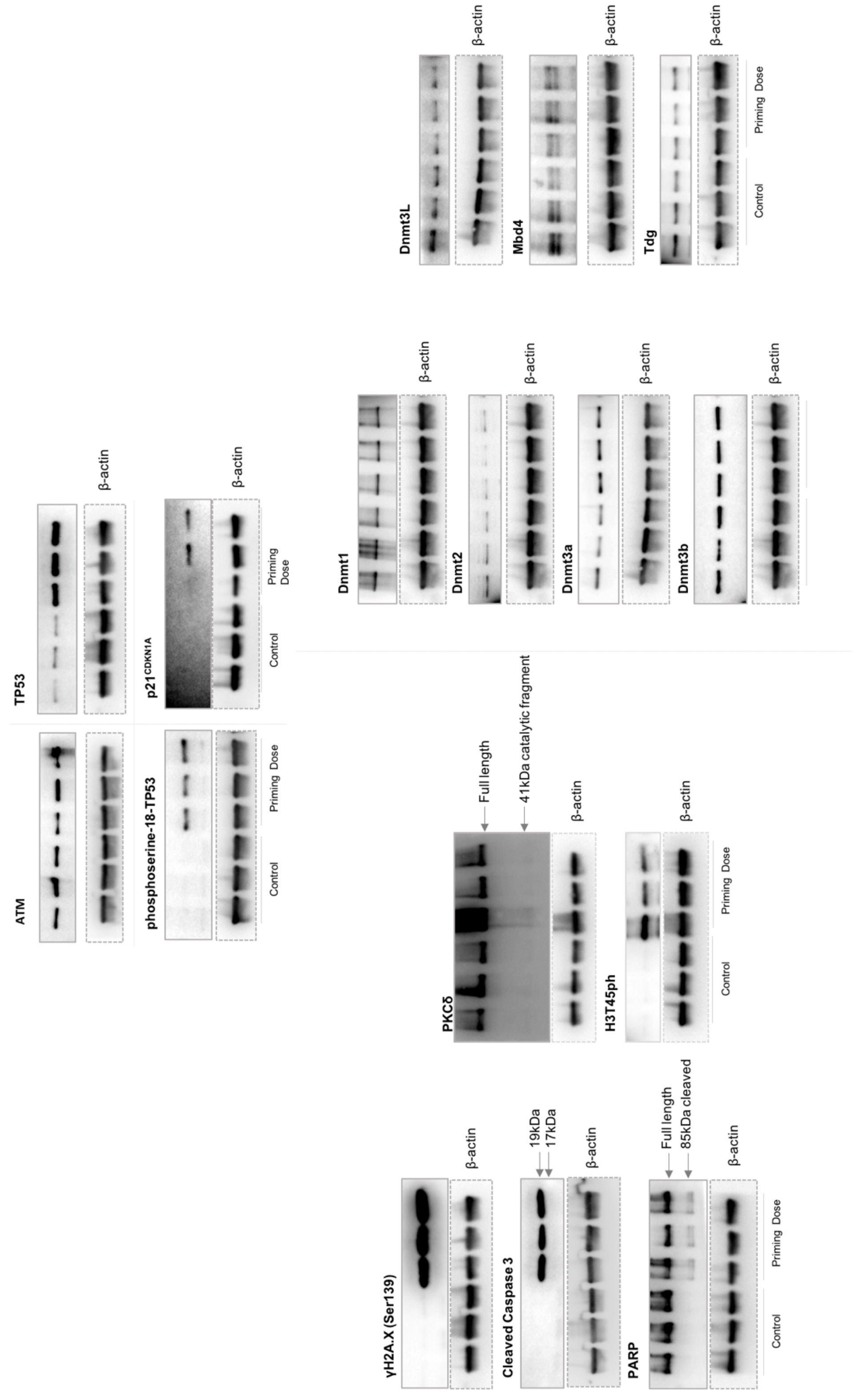

Annexed Figure 5

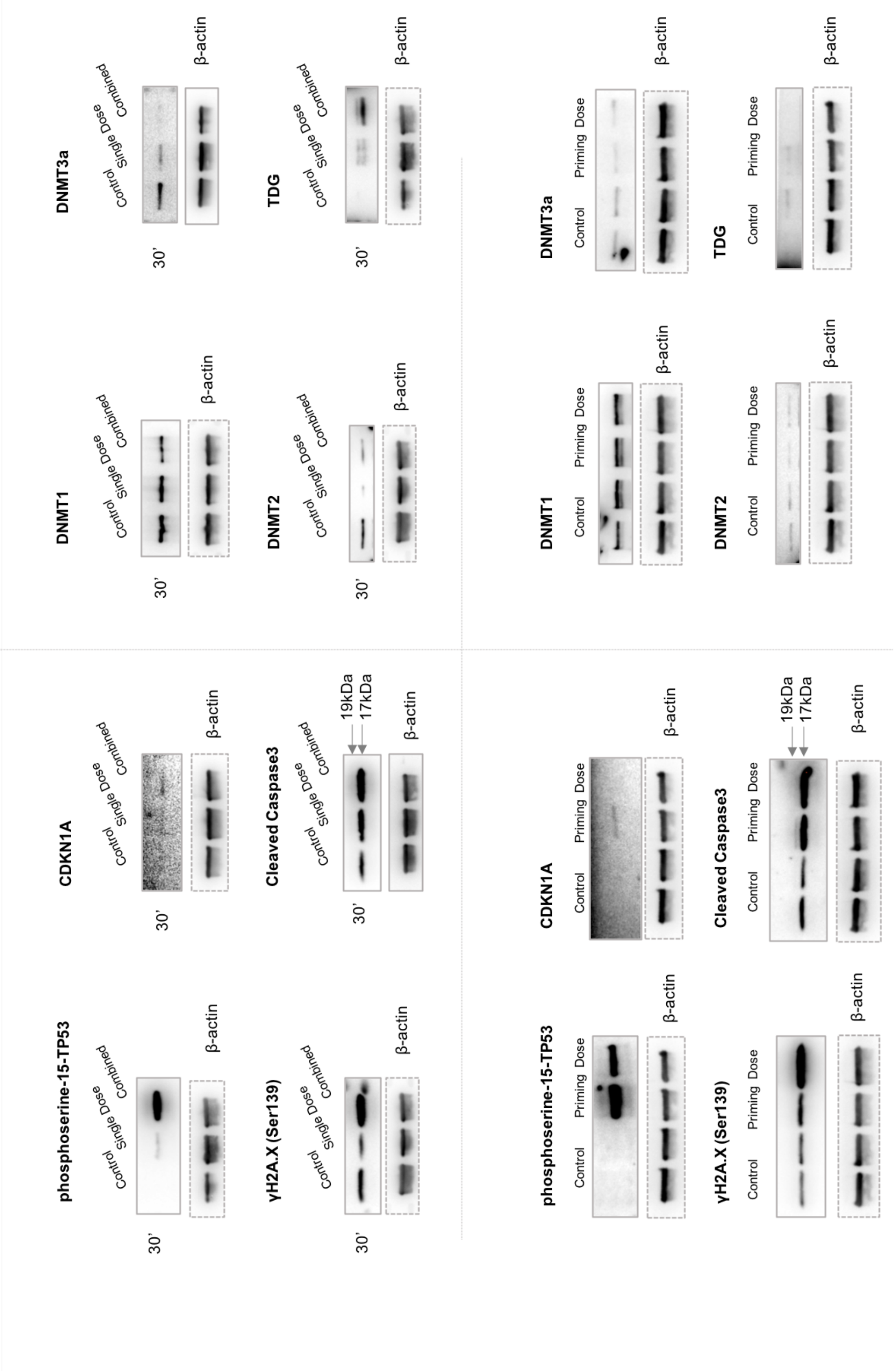

Supplementary Table 1

Supplementary Table 1.- Information on antibodies used in immunoblot analyses.

Supplier	Primary Antibodies	Cat. No	Molecular Weight (kDa)	Dilution	Blocking Solution	Source/Isotype
Abcam	Anti-Dnmt3b	ab122932	96	1/3000	NFDM 5%	RABBIT
	Anti-PKC delta antibody	ab182126	78/40	1/1000	NFDM 5%	RABBIT
	Anti-Dnmt2	ab71015	55	1/1000	NFDM 5%	RABBIT
	Anti-xCT antibody [EPR8290(2)]	ab175186	55	1/1000	NFDM 5%	RABBIT
	Anti-p21 antibody [EPR3993]	ab109199	21	1/1000	NFDM 5%	RABBIT
	Anti-Glutathione Peroxidase 4 antibody [EPNCIR144]	ab125066	17	1/1000	NFDM 5%	RABBIT
	Anti-Histone H3 (phospho T45)	ab26127	15	1/1000	BSA 5%	RABBIT
Cell Signaling	ATM (D2E2)	#2873	350	1/1000	BSA 5%	RABBIT
	DNMT1 (D63A6) XP	#5032	200	1/1000	BSA 5%	RABBIT
	DNMT3A (D23G1)	#3598	130	1/1000	BSA 5%	RABBIT
	PARP	#9542	116/85	1/1000	BSA 5%	RABBIT
	p53 (1C12)	#2524	53	1/1000	NFDM 5%	MOUSE
	Phospho-p53 (Ser15)	#9284	53	1/500	BSA 5%	RABBIT
	DNMT3L (E1Y7Q)	#13451	49	1/1000	BSA 5%	RABBIT
	Cleaved Caspase-3 (Asp175)	#9661	17/19	1/1000	NFDM 5%	RABBIT
	Phospho-Histone H2A.X (Ser139)	#2577	15	1/1000	BSA 5%	RABBIT
Epigentek	TDG Polyclonal Antibody	A-6704-100	55-65	1/1000	BSA 5%	RABBIT
Invitrogen	MBD4 Antibody (PA5-79639)	PA5-79639	60-65	1/1000	BSA 5%	RABBIT
Sigma	β-Actin (AC15)	A3854	42	1/5000	BSA 5%	MOUSE

Supplier	Secondary Antibodies	Cat. No	Dilution
Cell Signaling	Anti-rabbit IgG, HRP-linked Antibody	#7074	1/1000
	Anti-mouse IgG, HRP-linked Antibody	#7076	1/1000

Supplementary Table 2

Supplementary Table 2. OneStep qMethyl-PCR sequences of the DNA primers used in this study.

Gene Symbol		(5'–3') sequences	Size (bp)	Frequencies of Restriction Sites		
				AccII	HpaII	HpyCH4IV
AKT	F	GACCGAGCAGCGTCCTGT	266	11	8	✗
	R	GGCCACTTCCTGCTCCTACT				
ATM	F	TTTCGTGCCACACTAACCAA	242	2	2	✗
	R	GCTCCCCTCAAAACAGTCTC				
CASP3	F	TGGCCGAAAGACCTCTCTAA	232	3	2	✗
	R	CAACACCTCATCTCGCACAC				
CDKN1A	F	CGCTGCGTGACAAGAGAATA	203	4	2	✗
	R	TCGAGCTGCCTCCTTATAGC				
SLC7A11	F	AGGAAGCTGAGCTGGTGTGT	212	1	1	✗
	R	ACTGTTCGGTCGTGACTTCC				
TRP53	F	ATGGCGACTATCCAGCTTTG	244	1	1	✗
	R	AGAGGTCTCGTCACGCTCAT				
PARP1	F	GGGGAAACCGACACGTTAG	179	2	✗	1
	R	GGAGGGAGTCCTTGGGAATA				
PRKCD	F	CCCTTTGCACTTCCGTGT	154	3	4	✗
	R	GGGAGGTAGGCACAAGTCTG				
GPX4	F	GGAAAGCGGAGCCTGATAG	204	3	4	✗
	R	GTCTGTGCGTCCCAAGCA				

Supplementary Table 3

30 min

% cells	Control	Single	Combined
**sub-G1**	0.62 ± 0.02	1.48 ± 0.06	9.09 ± 0.66
**G1**	91.74 ± 0.56	89.47 ± 0.50	79.72 ± 1.52
**S**	5.51 ± 0.29	7.27 ± 0.09	3.45 ± 0.23
**G2/M**	1.77 ± 0.16	1.59 ± 0.26	7.37 ± 0.12

4 h

% cells	Control	Single	Combined
**sub-G1**	0.25 ± 0.02	16.20 ± 0.85	32.93 ± 5.11
**G1**	92.39 ± 0.58	73.03 ± 0.21	58.17 ± 4.91
**S**	5.61 ± 0.37	4.38 ± 0.87	2.09 ± 0.12
**G2/M**	1.70 ± 0.12	6.32 ± 0.61	6.79 ± 0.58

6 h

% cells	Control	Single	Combined
**sub-G1**	0.70 ± 0.05	19.05 ± 1.06	63.90 ± 1.27
**G1**	92.08 ± 0.84	72.99 ± 0.86	32.17 ± 0.34
**S**	4.72 ± 0.64	1.75 ± 0.12	3.44 ± 0.89
**G2/M**	2.28 ± 0.15	8.80 ± 0.66	0.77 ± 0.04

Supplementary Table 4

Supplementary Table 4: One Way ANOVA (Bonferroni´s multiple comparisons test) p-value Adjusted

			ATM	TP53	phosphoserine-18-TP53	p21^{CDKN1A}
Supporting Data Figure 1	30'	Control-Single Dose	ns	***	**	ns
		Control-Combined	ns	**	**	*
		Single Dose-Combined	ns	ns	ns	ns
	4H	Control-Single Dose	ns	**	*	**
		Control-Combined	ns	**	*	**
		Single Dose-Combined	ns	ns	ns	ns
	6H	Control-Single Dose	*	***	***	***
		Control-Combined	***	*	*	**
		Single Dose-Combined	*	**	**	ns

			γH2A.X (Ser139)	Cleaved Caspase 3 (p19)	Cleaved Caspase 3 (p17)	PARP (Full Lenght)	PARP (Cleaved)	PKCδ (Full Lenght)	PKCδ (Catalitic Fragment)	H3T45ph	SLC7A11	GPX4
Supporting Data Figure 2	30min	Control-Single Dose	ns	ns	ns	ns	ns	ns	ns	ns	ns	ns
		Control-Combined	****	ns	**	ns	*	ns	ns	***	ns	ns
		Single Dose-Combined	**	ns	**	ns	*	ns	ns	***	ns	ns
	4H	Control-Single Dose	***	**	****	**	*	ns	ns	**	ns	ns
		Control-Combined	**	**	****	**	**	ns	ns	**	**	*
		Single Dose-Combined	ns	ns	ns	ns	ns	ns	ns	ns	ns	ns
	6H	Control-Single Dose	**	ns	ns	*	*	ns	ns	*	ns	ns
		Control-Combined	***	ns	*	**	**	ns	ns	**	**	ns
		Single Dose-Combined	ns	ns	ns	ns	ns	ns	**	ns	**	ns

			Dnmt1	Dnmt2	Dnmt3a	Dnmt3b	Dnmt3L	Mbd4	Tdg
Supporting Data Figure 3	30min	Control-Single Dose	ns	ns	ns	ns	ns	ns	ns
		Control-Combined	ns	ns	ns	*	ns	**	ns
		Single Dose-Combined	ns	ns	ns	*	ns	ns	ns
	4H	Control-Single Dose	ns	ns	**	ns	ns	ns	ns
		Control-Combined	ns	ns	ns	*	ns	ns	ns
		Single Dose-Combined	ns	ns	*	**	ns	ns	ns
	6H	Control-Single Dose	ns	ns	ns	**	ns	*	**
		Control-Combined	ns	*	***	***	***	**	**
		Single Dose-Combined	ns	ns	**	ns	**	ns	ns

Supporting Data Figure 4

		ATM	TP53	phosphoserine-18-TP53	p21^{CDKN1A}
6H	Control-Priming	ns	****	**	*

		γH2A.X (Ser139)	Cleaved Caspase 3 (p17)	PARP (Full Lenght)	PARP (Cleaved)	PKCδ (Full Lenght)	PKCδ (Catalitic Fragment)	H3T45ph
6H	Control-Priming	***	****	ns	****	ns	ns	***

		Dnmt1	Dnmt2	Dnmt3a	Dnmt3b	Dnmt3L	Mbd4	Tdg
6H	Control-Priming	ns	ns	ns	ns	ns	ns	ns

Supporting Data Figure 5

		phosphoserine-15-TP53	CDKN1A
6H	Control-Single Dose	ns	****
	Control-Combined	***	****
	Single Dose-Combined	***	**

		γH2A.X (Ser139)	Cleaved Caspase 3 (p17)
6H	Control-Single Dose	ns	*
	Control-Combined	****	****
	Single Dose-Combined	****	***

		DNMT1	DNMT2	DNMT3a	TDG
6H	Control-Single Dose	****	****	****	***
	Control-Combined	****	****	****	****
	Single Dose-Combined	****	***	*	***

		phosphoserine-15-TP53	CDKN1A
6H	Control-Priming	****	ns

		γH2A.X (Ser139)	Cleaved Caspase 3 (p17)
6H	Control-Priming	ns	*

		DNMT1	DNMT2	DNMT3a	TDG
6H	Control-Priming	ns	ns	*	ns

Symbol meaning: ns P > 0.05, * P ≤ 0.05, ** P ≤ 0.01, *** P ≤ 0.001, **** P ≤ 0.0001 (For the last two choices only).

Supplementary Table 5

6 h		
% cells	Control	Priming
sub-G1	0.70 ± 0.05	10.15 ± 0.07
G1	92.08 ± 0.84	81.95 ± 4.12
S	4.72 ± 0.64	1.67 ± 0.09
G2/M	2.28 ± 0.15	8.58 ± 1.62

Supplementary Table 6

% cells	Control	Single	Combined
sub-G1	15.60 ± 0.14	13.00 ± 0.14	30.50 ± 0.71
G1	74.10 ± 0.14	78.75 ± 0.07	59.15 ± 0.07
S	4.90 ± 0.00	3.80 ± 0.14	5.30 ± 0.42
G2/M	5.25 ± 0.07	4.20 ± 0.14	4.55 ± 0.35

% cells	Control	Priming
sub-G1	15.60 ± 0.14	19.10 ± 0.57
G1	74.10 ± 0.14	69.05 ± 0.49
S	4.90 ± 0.00	5.50 ± 0.14
G2/M	5.25 ± 0.07	6.00 ± 0.28

Supplementary Figure 1

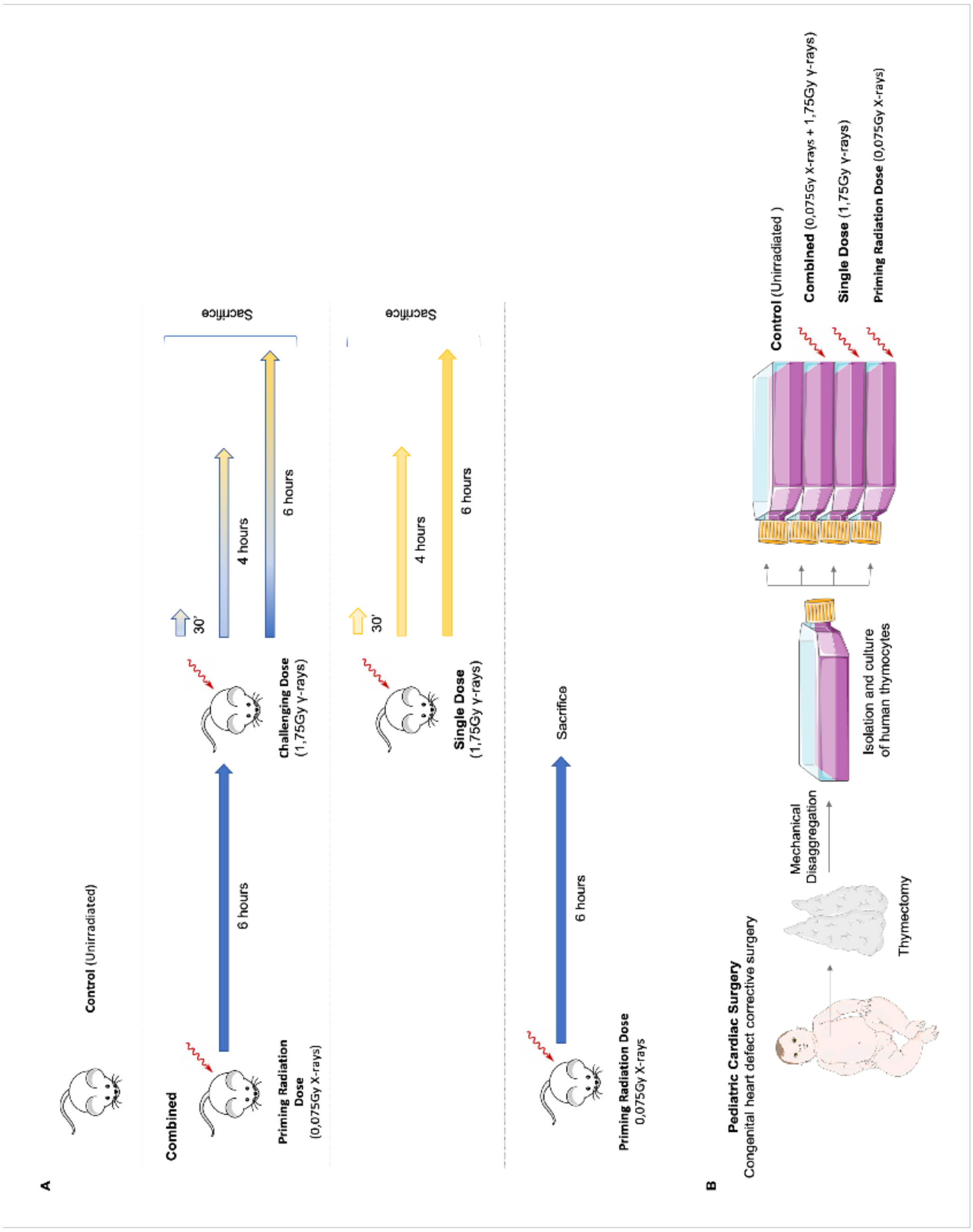

Supplementary Figure 2

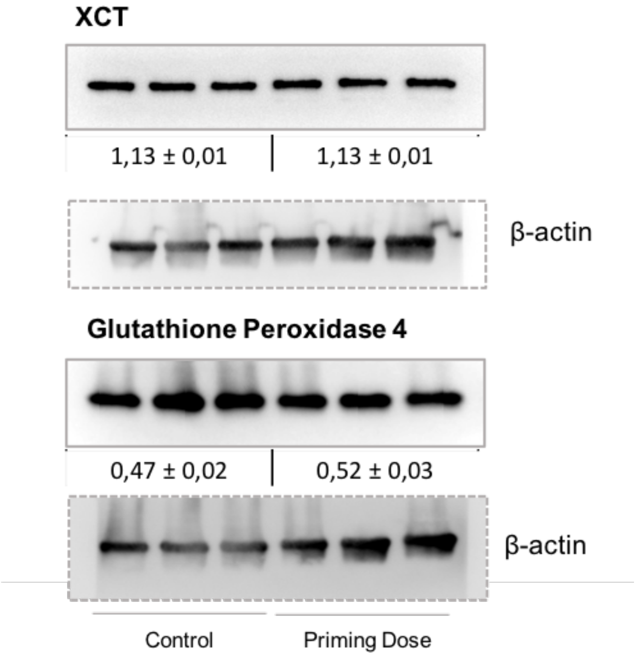

Supplementary Figure 3

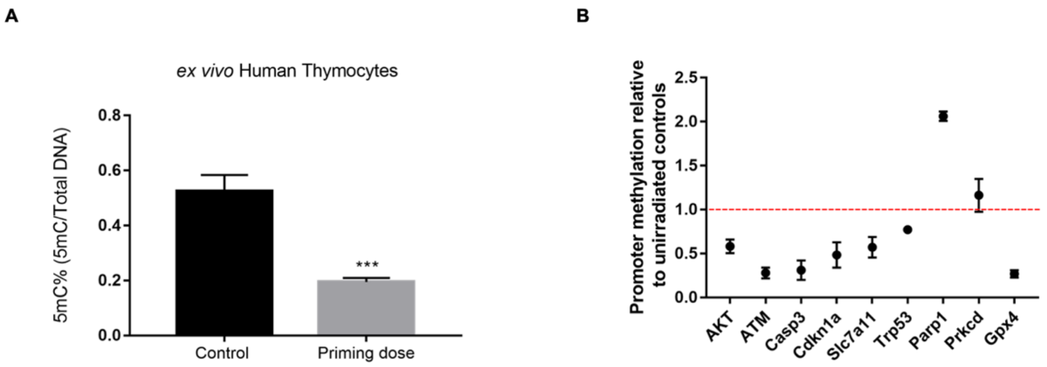

Supplementary Figure 4

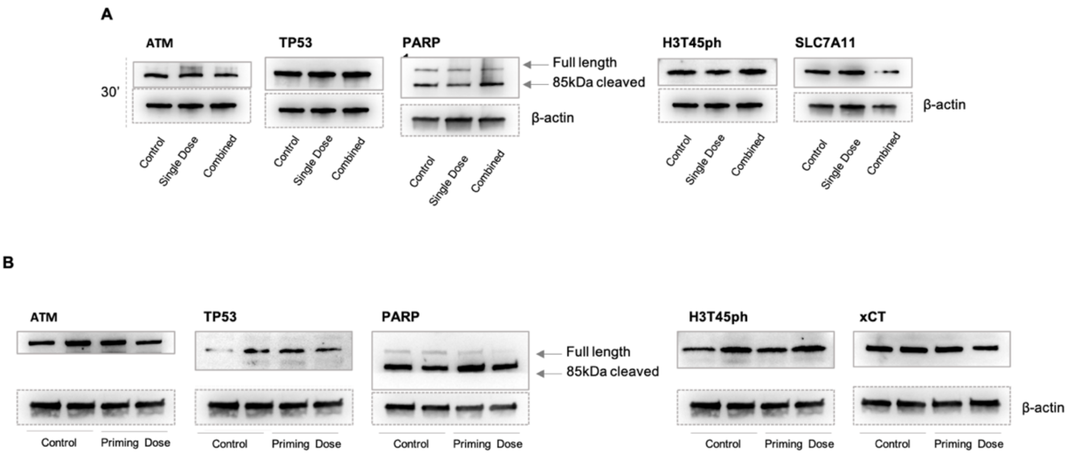

Supplementary table 1: Information on the antibodies used in this study.

Supplementary table 2: Methylation primers used for the promoter methylation study with the OneStep qMethyl™ Kit.

Supplementary table 3: Cell cycle study mean and standard deviation for each time, dose (combined or single dose) when compared to their control counterparts and for each cell cycle phase. The experiment was repeated 3 times for each cell type and condition.

Supplementary table 4: Significations of ordinary one-way analysis of variance (ANOVA) Bonferroni's multiple comparisons to examine pairwise and subgroup differences (* $p \leq 0.05$; ** $p \leq 0.01$; *** $p \leq 0.001$ and **** $p \leq 0.0001$). Test was performed using GraphPad Prism version 9.3.1 for Mac OS X, (GraphPad Software, San Diego, California USA, www.graphpad.com).

Supplementary table 5: Cell cycle study mean and standard deviation for the cohort irradiated with the priming dose and collected 6 hours after the irradiation when compared to their control counterparts and for each cell cycle phase in mouse thymocytes. The experiment was repeated 3 times for each cell type and condition.

Supplementary table 6: Cell cycle study mean and standard deviation for the cohort irradiated with the priming dose and collected 6 hours after the irradiation when compared to their control counterparts and for each cell cycle phase in human thymocytes. The experiment was repeated 3 times for each cell type and condition.

Supplementary Figure 1: Radiation schemes (A) Radiation scheme for the different mice used in this study. (B) Radiation scheme of the human pediatric thymus.

Supplementary Figure 2: Ferroptosis markers study 6 hours after the priming dose in mice. xCT and Glutation peroxidase 4 (GPX4) analysis 6 hours after the priming dose alone. Each membrane shows 3 biological replicates for the control animals and 3 for those irradiated with the priming dose alone and sacrificed 6 hours after exposure. β-Actin was probed as loading control.

Supplementary Figure 3: Methylation study. (A) Global methylation assay on thymocytes exposed to the priming dose assayed 6 hours after exposure. Black bar depicts the control cohort and the grey bar the animals exposed to the priming dose. Results show the mean and standard deviation of 3 biological replicates, *** *p* value ≤

0.001. **(B)** Promoter methylation study of specific genes. Dashed line shows the level of the unirradiated control and the promoter methylation levels below the line are hypomethylated whilst those above the line are hypermethylated (PARP1 and PKCδ). Results show the mean and standard deviation of three biological replicates. Test were performed using GraphPad Prism version 9.3.1 for Mac OS X, (GraphPad Software, San Diego, California USA, www.graphpad.com)

Supplementary Figure 4: *Ex vivo* radio-adaptative response in human thymocytes. Western blot analysis of several proteins **(A)** Study combined radiation regimen response and **(B)** 6 hours after exposure to the priming dose (0.0075Gy) of ATM-TP53-phosphoserine-15-TP53 axis: ataxia telangiectasia mutated protein (ATM), tumor protein p53 (TP53); Apoptosis response marker Poly (ADP-ribose) polymerase-1 (PARP1), Phosphorylation of H3T45 (H3T45ph) and Ferroptosis response markers: xCT). (β-Actin was probed as a loading control for each membrane).

Adequacy of the comparison of results between mice and humans.

The overall structure of the immune system in mice and humans is quite similar. Of the various lymphoid tissues, the thymus is histologically perhaps the most consistent across species and descriptions for one species generally substitute for that of others. Ageing is characterised by cellular senescence, leading to imbalanced tissue maintenance, cell death and compromised organ function[1]. Ageing of the immune system first manifests as a dramatic involution of the thymus., in older animals functional thymic tissue is largely replaced by adipose tissue (atrophy/involution) and epithelial structures (cords, tubules, cysts) become more prominent.

Thymic size is already compromised in humans by the second year of life, decreases further during puberty, and continuously declines thereafter[2–4].

In mice, thymus morphological changes beging to take place by 4 weeks of age in female C57BL/6 mice, including cortical thinning and the coalescence of medullary islands. These gross tissue changes coincided with changes in thymocyte and TEC cellularity[5–7] . Total thymic and TEC cellularity halved between 4 and 16 weeks of age. Recent studies demonstrate that dynamic changes in mice thymuses indicate that the principal immune functions of the thymus are progressively compromised with involution[8].

To date, most quantitative studies focused on the history of thymus development are based on the determination of cell populations by flow cytometry, so to determine whether segregation CD4/CD8 ratios were comparable between humans and mice at the ages used in this work we performed a flow cytometric study of these populations. With the results obtained and taking into account the intra-species differences, we can assure that the population profiles of the 4-week-old mice (before the onset of organ

involution) and the human cells from pediatric thymuses used in this study have very similar population profiles as shown in the following image.

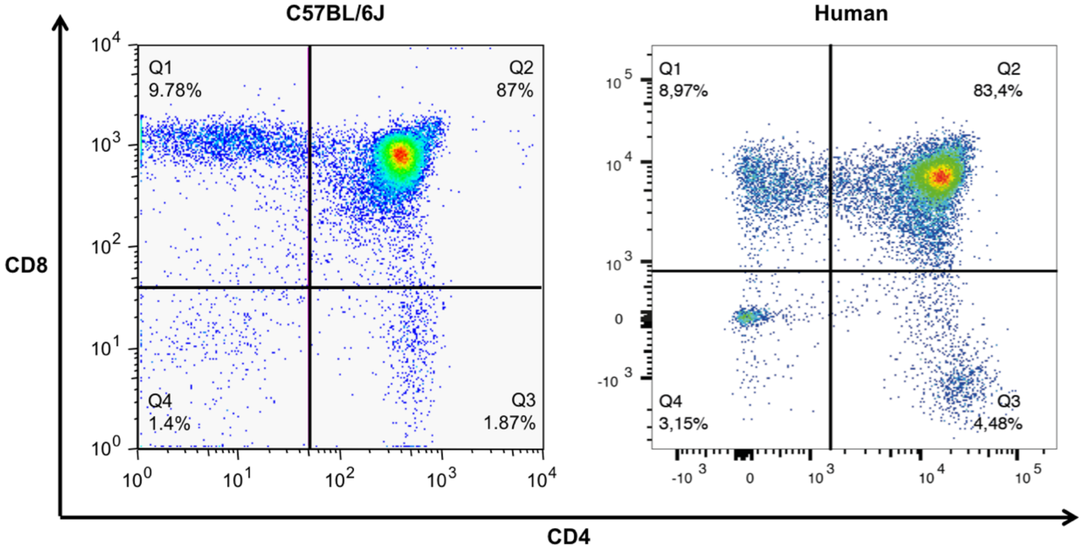

Flow cytometry analysis expression of CD4 and CD8 thymocytes of C57BL/6J mice and Human samples. SP8: CD8+CD4− (Q1); DP: CD4+CD8+ (Q2); SP4: CD4+CD8− (Q3). DN: CD4-CD8− (Q4). Quantitative data above the plots represent mean absolute values ± SD of three independent experiments. Test were performed using GraphPad Prism version 9.3.1 for Mac OS X, (GraphPad Software, San Diego, California USA, www.graphpad.com)

1. López-Otín, C., Blasco, M. A., Partridge, L., Serrano, M. & Kroemer, G. The hallmarks of aging. *Cell* **153**, 1194–1217 (2013).
2. Kumar, B. V., Connors, T. J. & Farber, D. L. Human T Cell Development, Localization, and Function throughout Life. *Immunity* **48**, 202–213 (2018).
3. Linton, P. J. & Dorshkind, K. Age-related changes in lymphocyte development and function. *Nat Immunol* **5**, 133–139 (2004).
4. Palmer, D. B. The effect of age on thymic function. *Front Immunol* **4**, 316 (2013).

5. Gray, D. H. D. *et al.* Developmental kinetics, turnover, and stimulatory capacity of thymic epithelial cells. *Blood* **108**, 3777–3785 (2006).
6. Manley, N. R., Richie, E. R., Blackburn, C. C., Condie, B. G. & Sage, J. Structure and function of the thymic microenvironment. *Front Biosci (Landmark Ed)* **16**, 2461–2477 (2011).
7. Ki, S. *et al.* Global transcriptional profiling reveals distinct functions of thymic stromal subsets and age-related changes during thymic involution. *Cell Rep* **9**, 402–415 (2014).
8. Ageing compromises mouse thymus function and remodels epithelial cell differentiation - PubMed. https://pubmed.ncbi.nlm.nih.gov/32840480/.

These figures show the pattern of the marker (Precision Plus Protein™ Dual Color, Bio-Rad (Hercules, CA) and the bands that were used for orientation when cutting the membrane. In order to clarify how the membranes were processed, it is shown, for each of the figures, how the membranes were cut

Figures 1-3

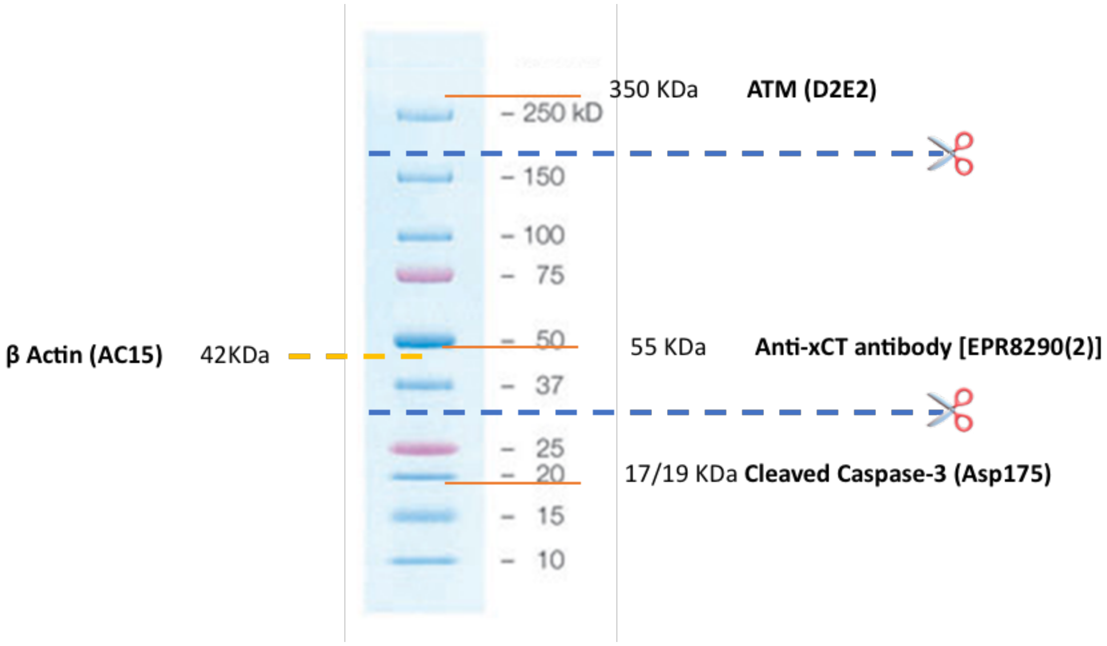

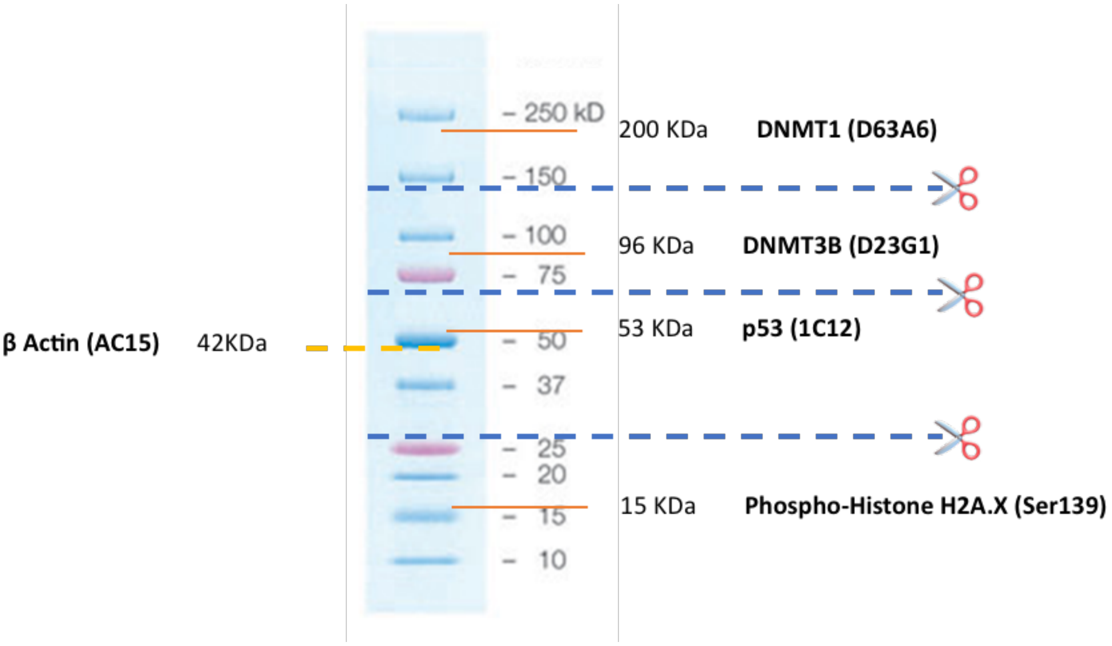

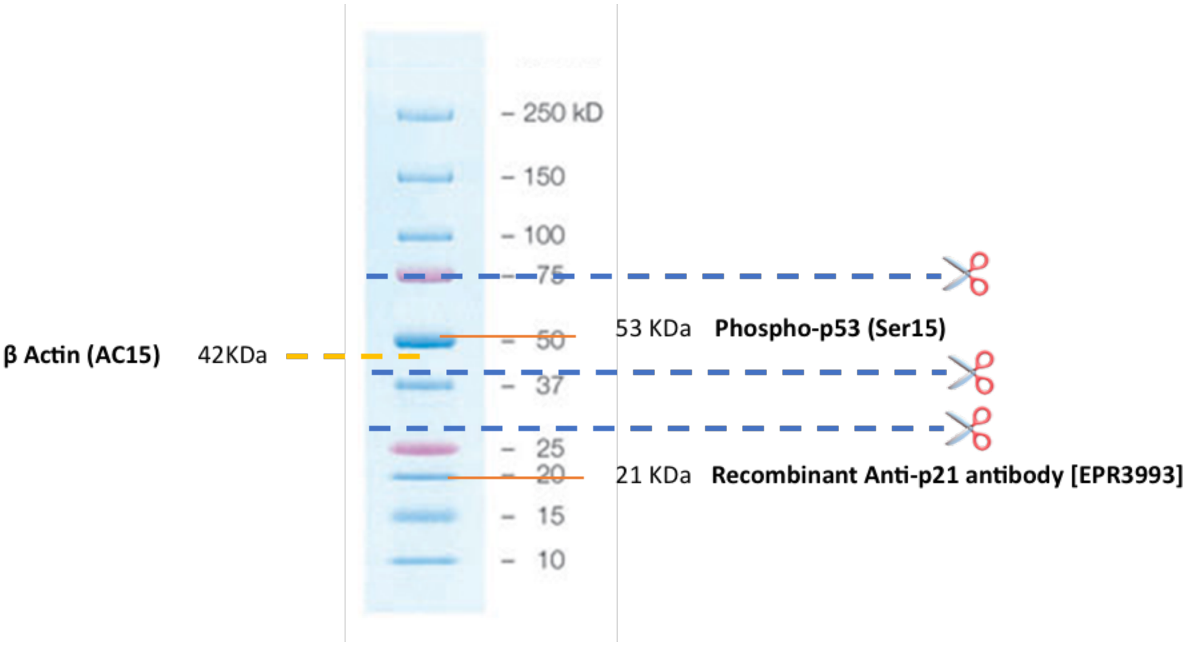
250 kD
150
100
75
50
37
25
20
15
10
53 KDa Phospho-p53 (Ser15)
β Actin (AC15) 42KDa
21 KDa Recombinant Anti-p21 antibody [EPR3993]

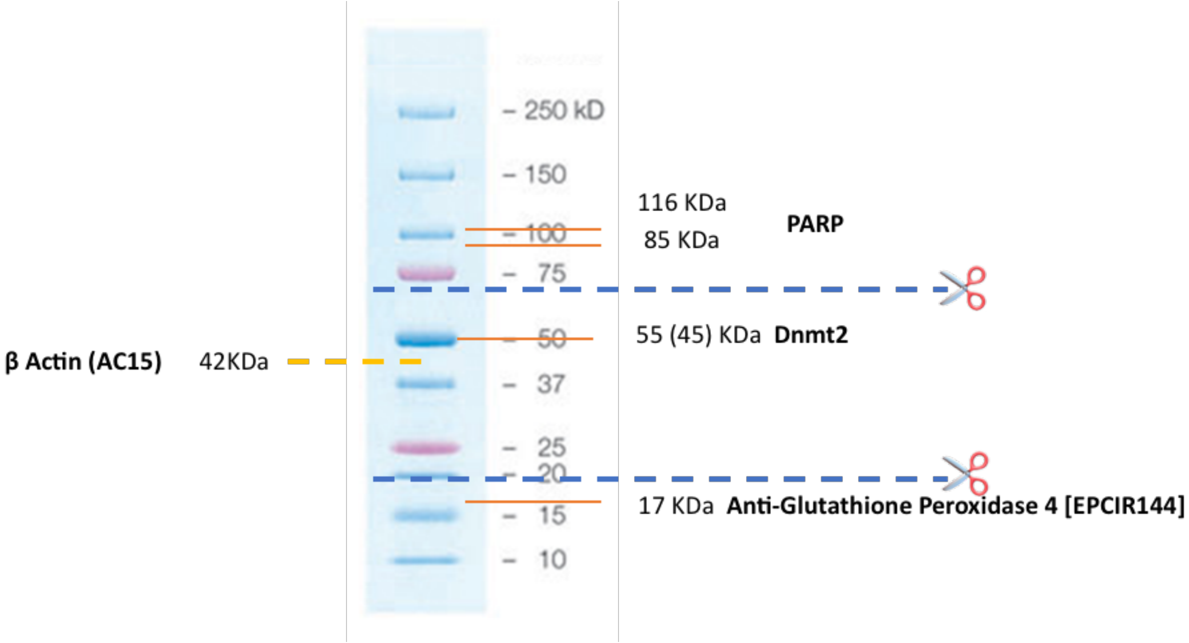
250 kD
150
100
75
50
37
25
20
15
10
116 KDa
85 KDa
PARP
55 (45) KDa Dnmt2
β Actin (AC15) 42KDa
17 KDa Anti-Glutathione Peroxidase 4 [EPCIR144]

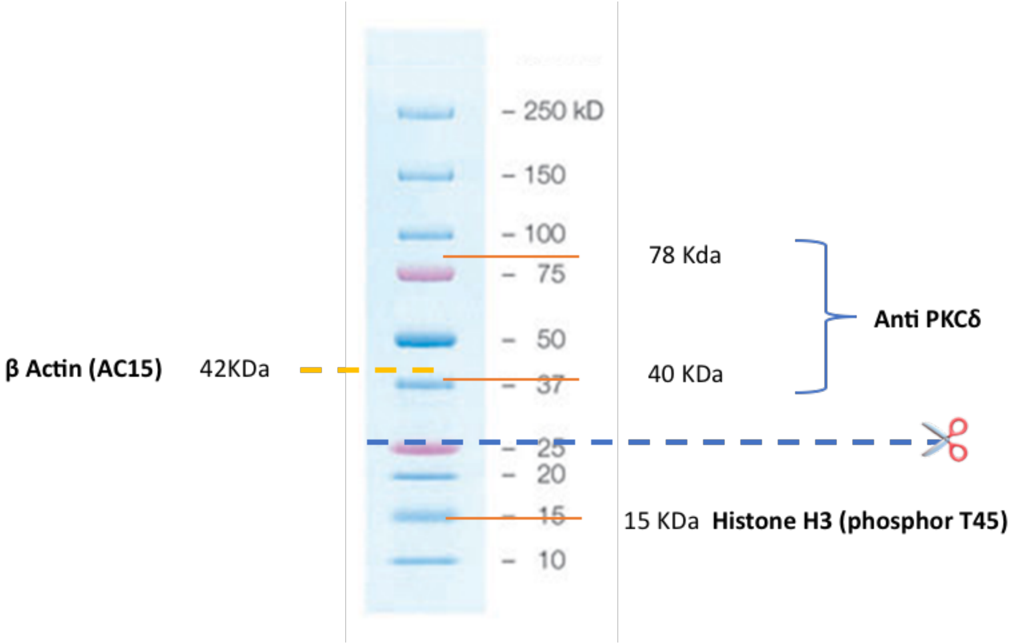

250 kD
150
100
75
50
37
25
20
15
10
78 Kda
40 KDa
Anti PKCδ
β Actin (AC15)
42KDa
15 KDa
Histone H3 (phosphor T45)

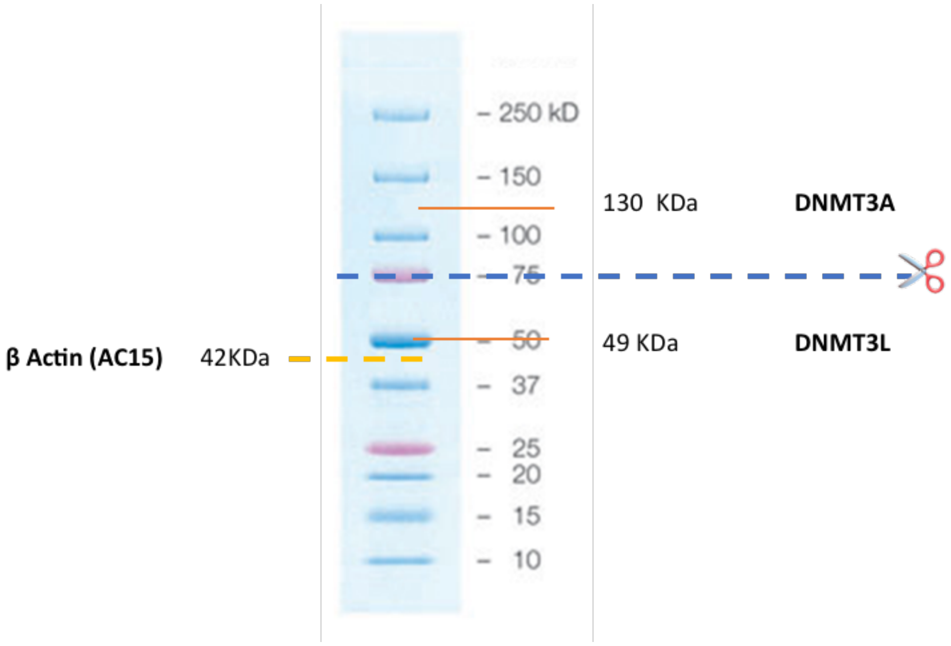

250 kD
150
100
75
50
37
25
20
15
10
130 KDa
DNMT3A
49 KDa
DNMT3L
β Actin (AC15)
42KDa

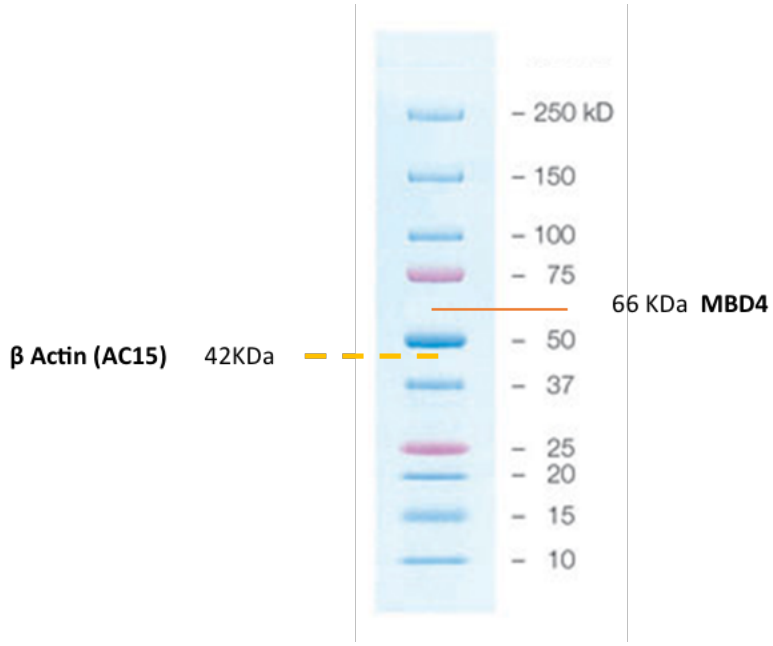

– 250 kD
– 150
– 100
– 75
– 50
– 37
– 25
– 20
– 15
– 10
66 KDa **MBD4**
β Actin (AC15) 42KDa

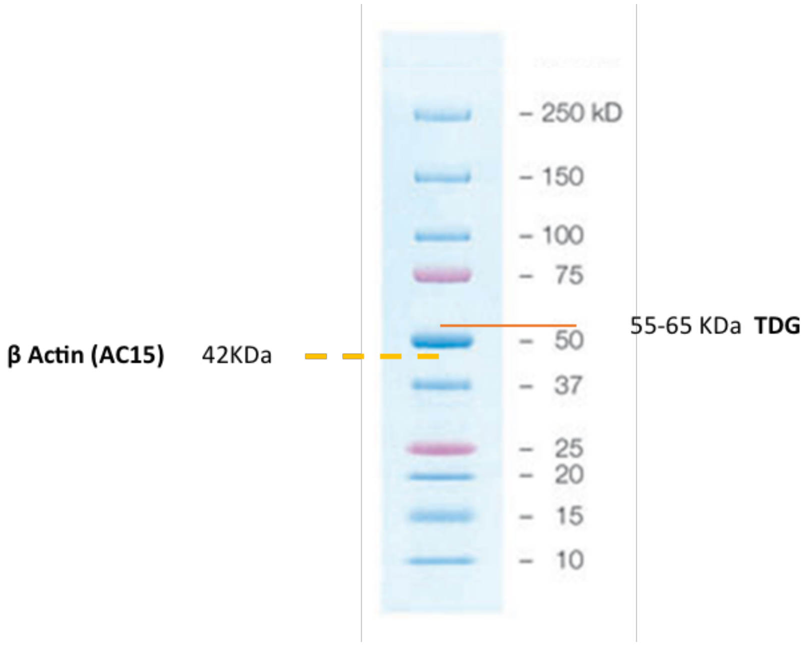

– 250 kD
– 150
– 100
– 75
– 50
– 37
– 25
– 20
– 15
– 10
55-65 KDa **TDG**
β Actin (AC15) 42KDa

Figure 4 and Supplementary Figure 2

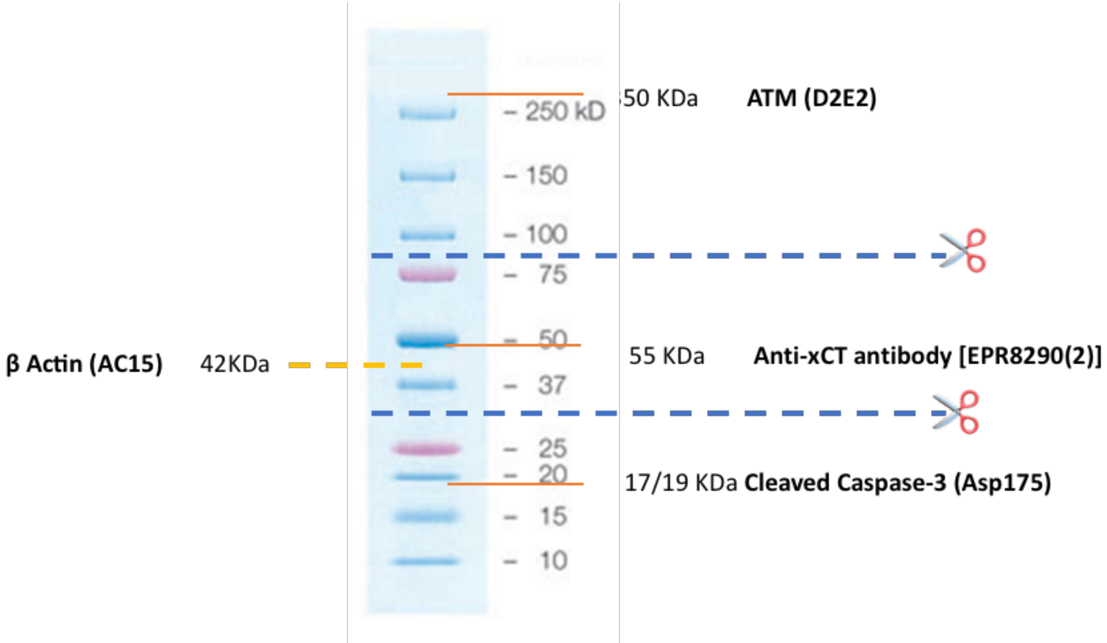

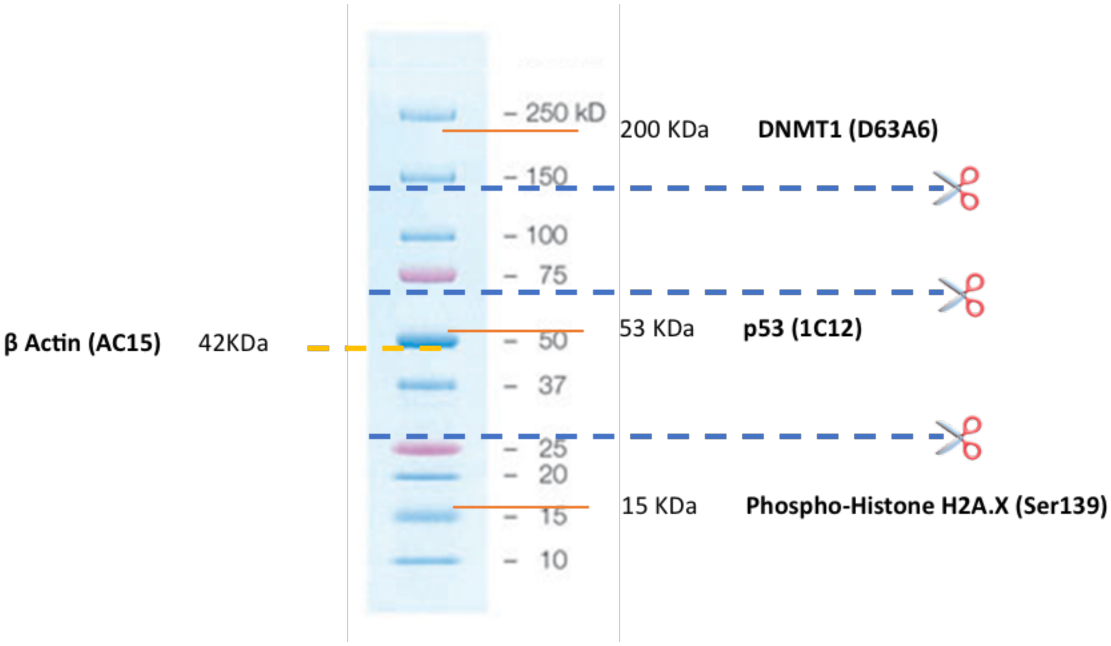

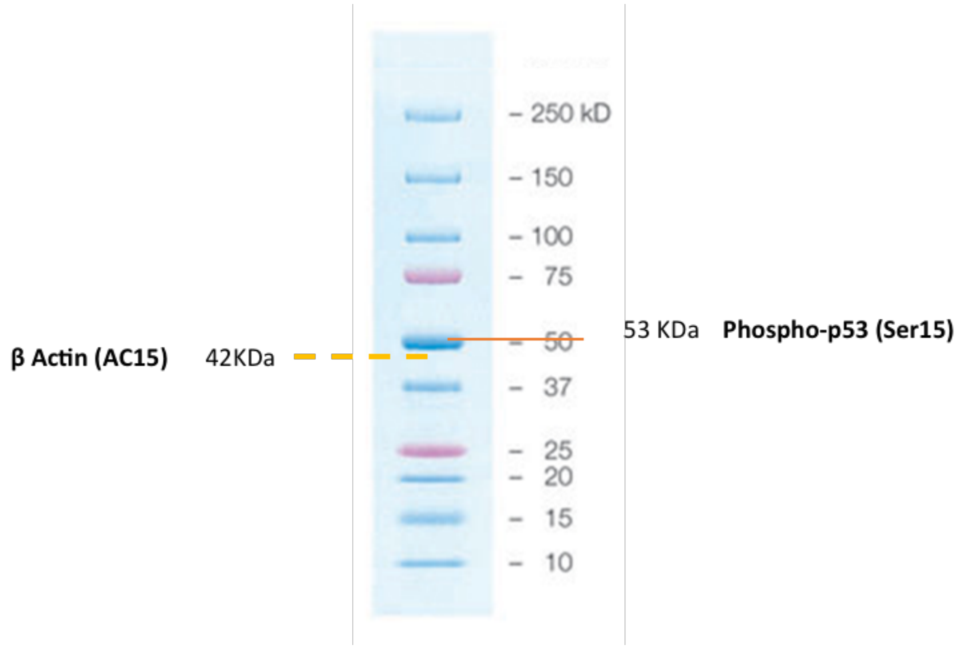
– 250 kD
– 150
– 100
– 75
β Actin (AC15) 42KDa
53 KDa Phospho-p53 (Ser15)
– 50
– 37
– 25
– 20
– 15
– 10

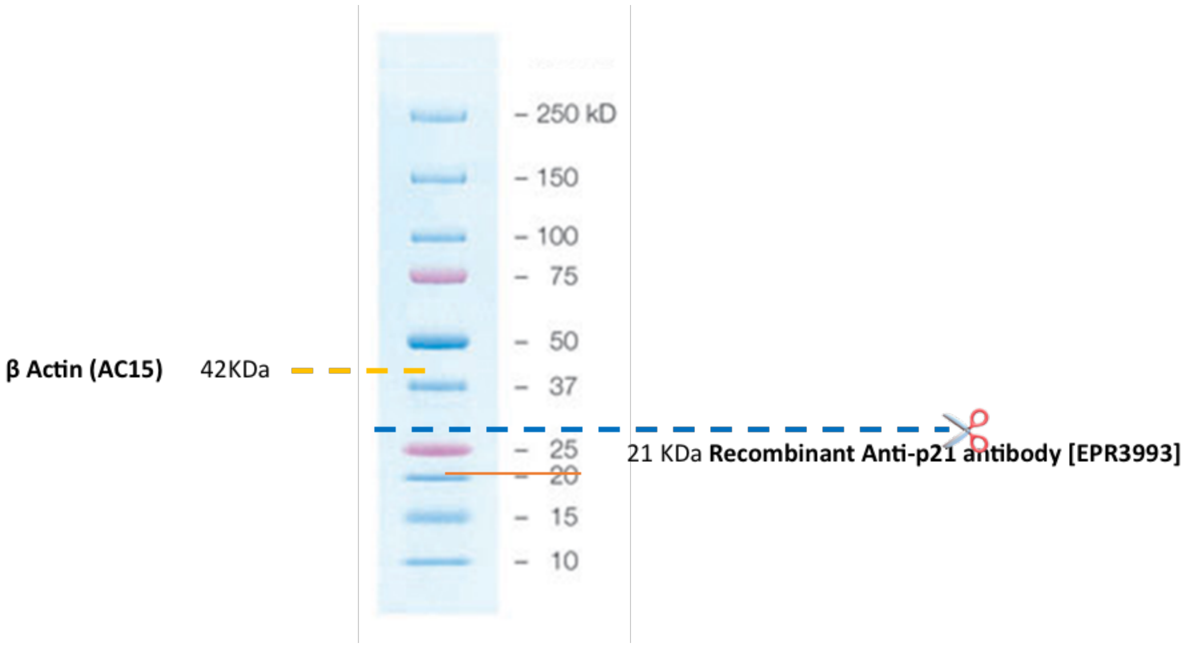
– 250 kD
– 150
– 100
– 75
– 50
β Actin (AC15) 42KDa
– 37
– 25
21 KDa Recombinant Anti-p21 antibody [EPR3993]
– 20
– 15
– 10

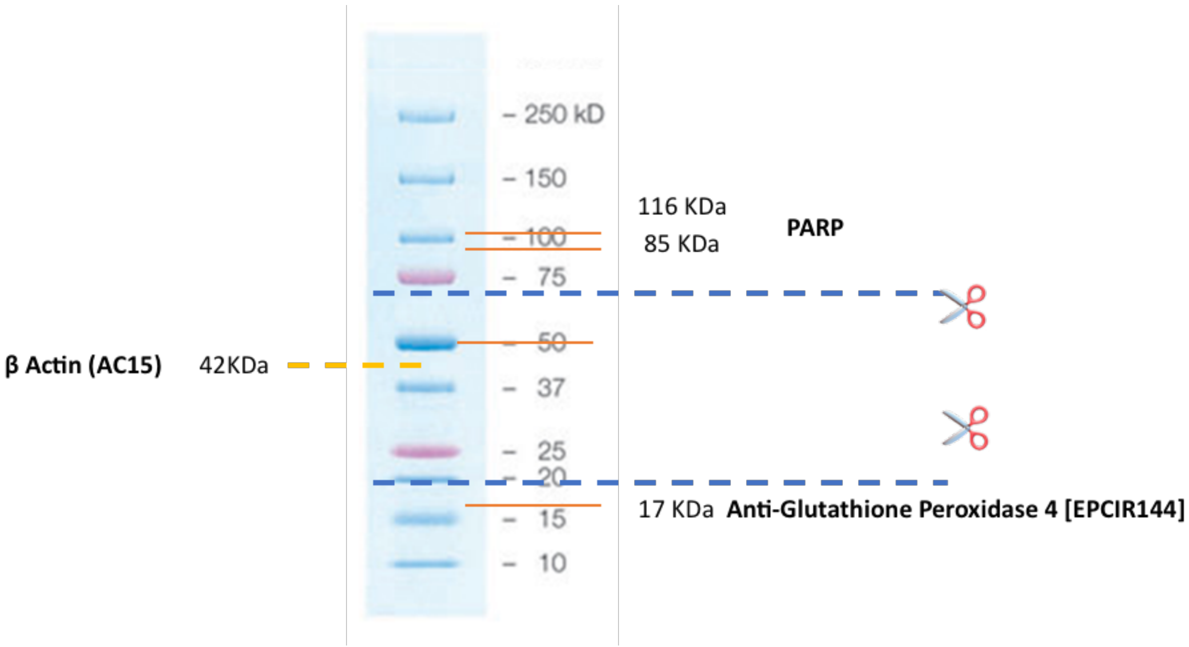
250 kD
150
100
75
50
37
25
20
15
10
116 KDa
85 KDa
PARP
β Actin (AC15)
42KDa
17 KDa
Anti-Glutathione Peroxidase 4 [EPCIR144]

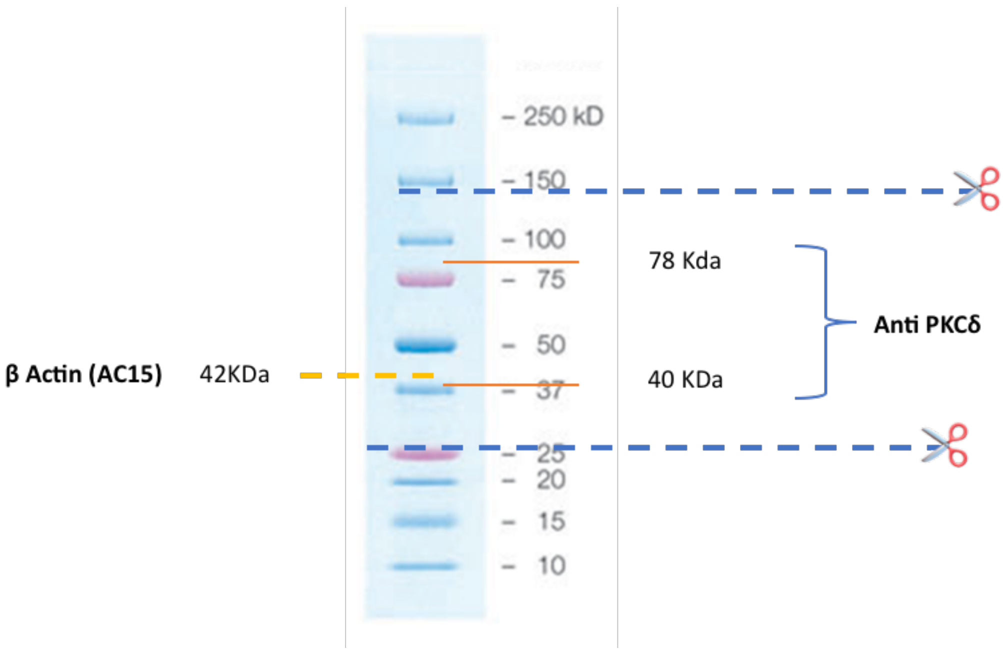
250 kD
150
100
75
50
37
25
20
15
10
78 Kda
40 KDa
Anti PKCδ
β Actin (AC15)
42KDa

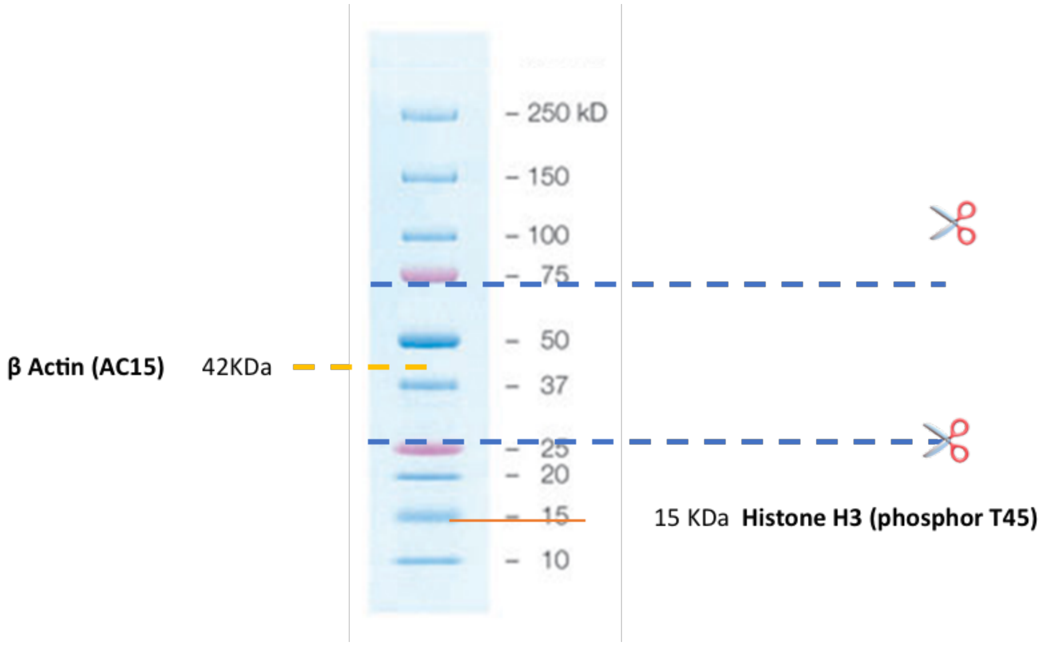
250 kD
150
100
75
50
37
25
20
15
10
β Actin (AC15) 42KDa
15 KDa Histone H3 (phosphor T45)

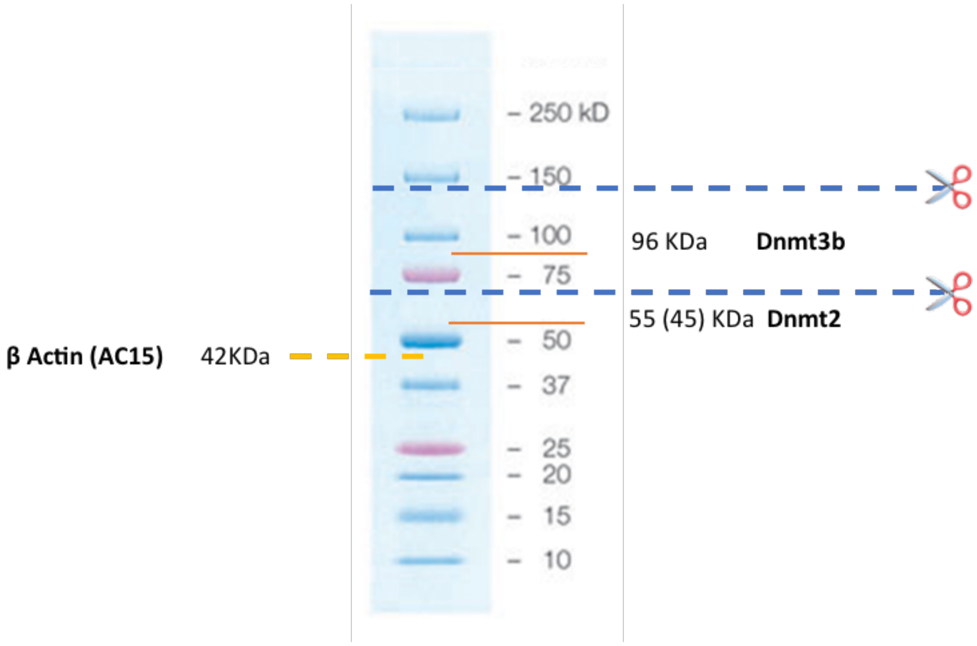
250 kD
150
100
75
50
37
25
20
15
10
96 KDa Dnmt3b
55 (45) KDa Dnmt2
β Actin (AC15) 42KDa

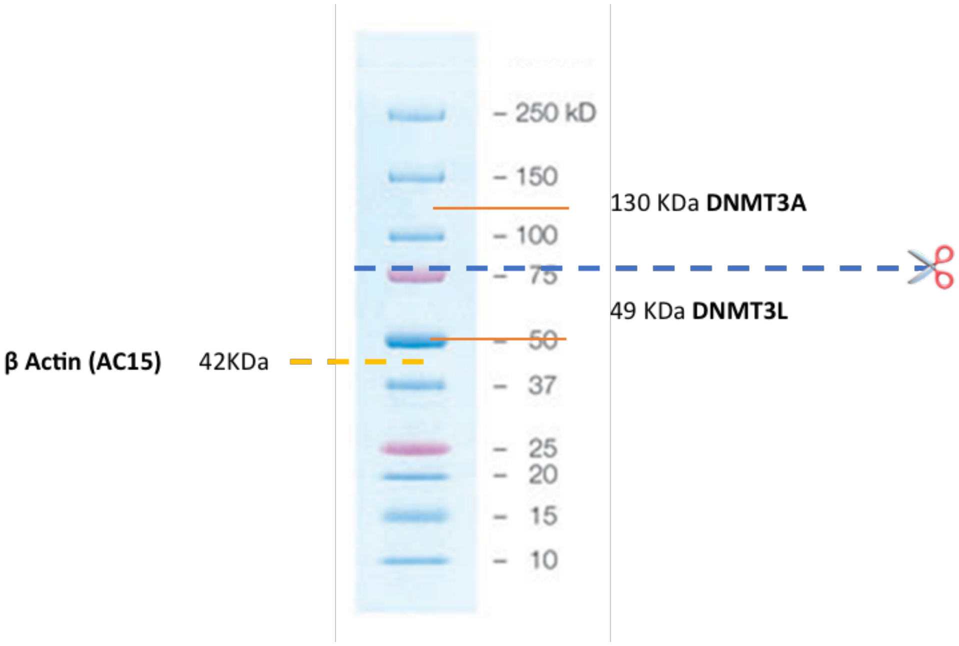
250 kD
150
100
75
50
37
25
20
15
10
130 KDa **DNMT3A**
49 KDa **DNMT3L**
β Actin (AC15) 42KDa

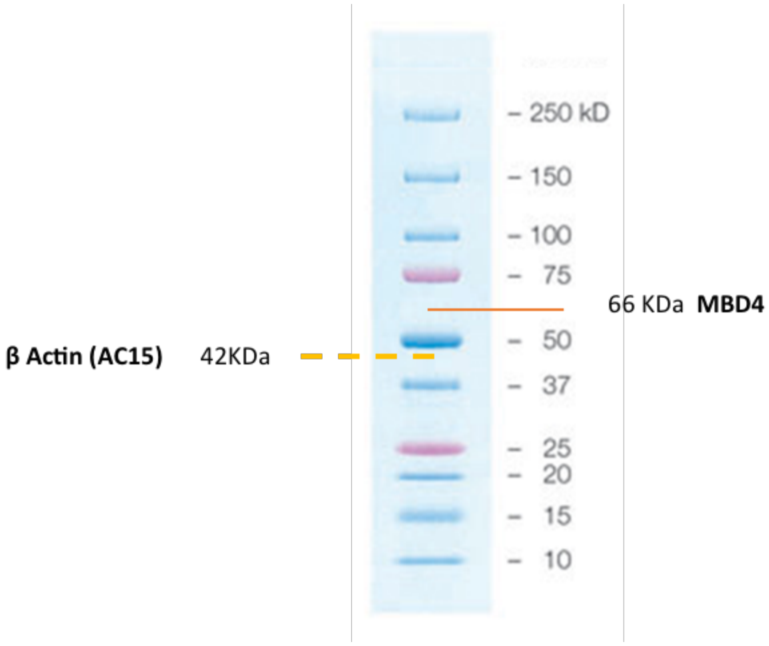
250 kD
150
100
75
50
37
25
20
15
10
66 KDa **MBD4**
β Actin (AC15) 42KDa

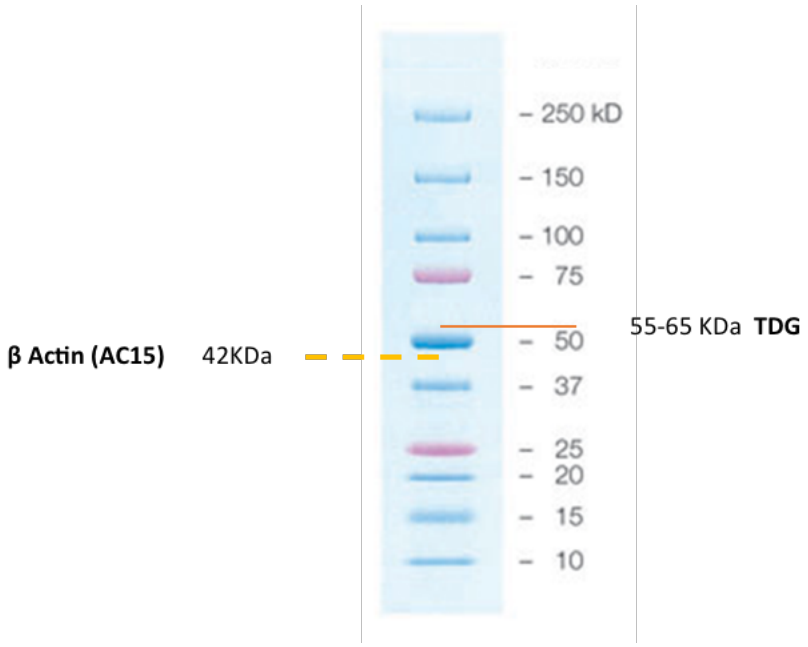

β Actin (AC15)
42KDa
55-65 KDa TDG
250 kD
150
100
75
50
37
25
20
15
10

Figure 5 and Supplementary figure 4

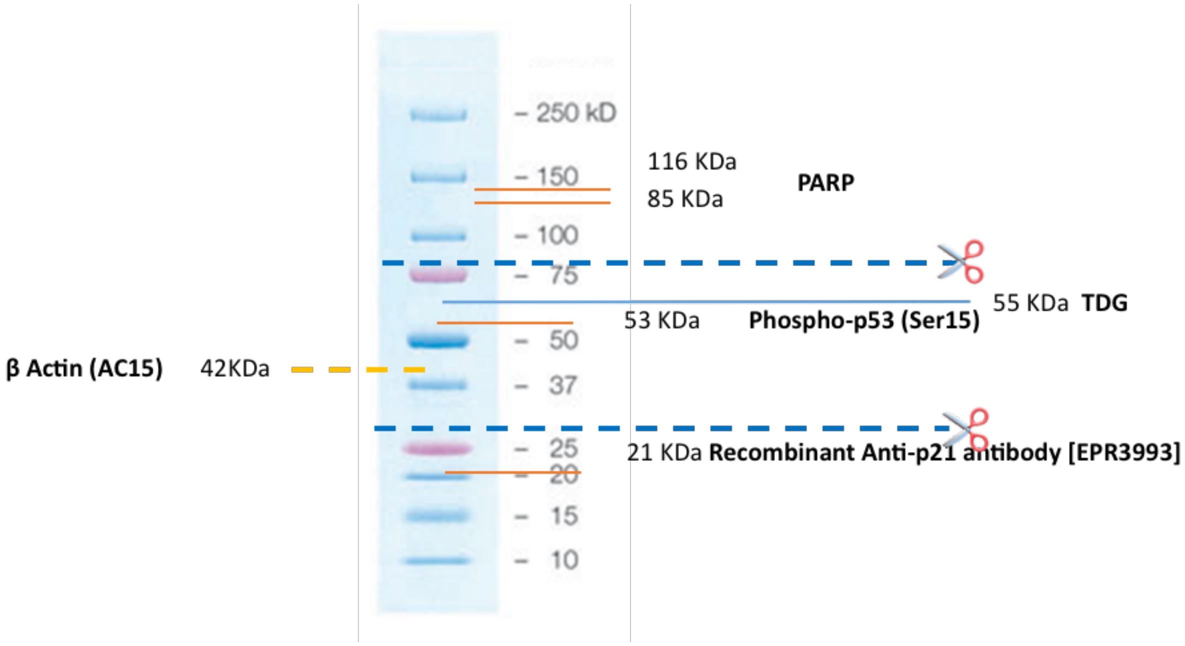

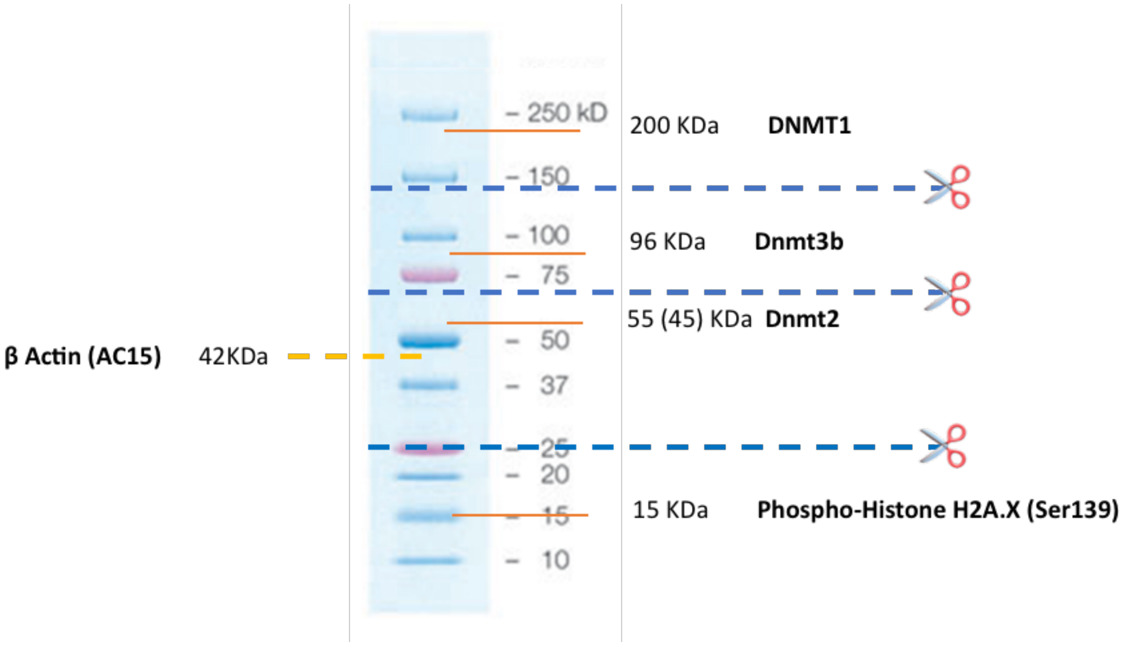

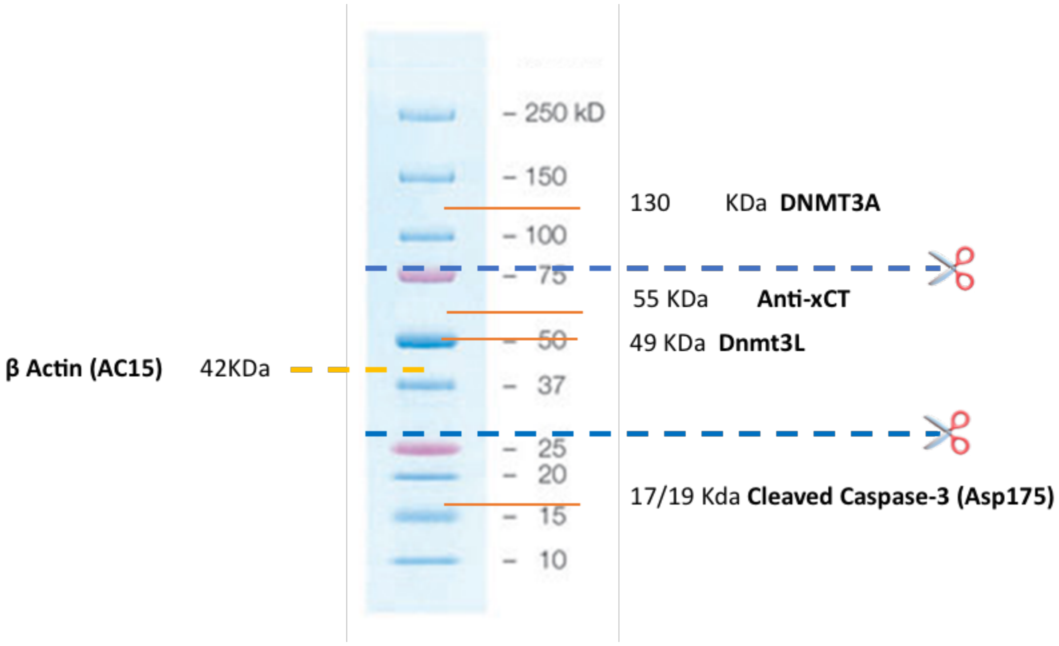
250 kD
150
100
75
50
37
25
20
15
10
130 KDa DNMT3A
55 KDa Anti-xCT
49 KDa Dnmt3L
β Actin (AC15) 42KDa
17/19 Kda Cleaved Caspase-3 (Asp175)

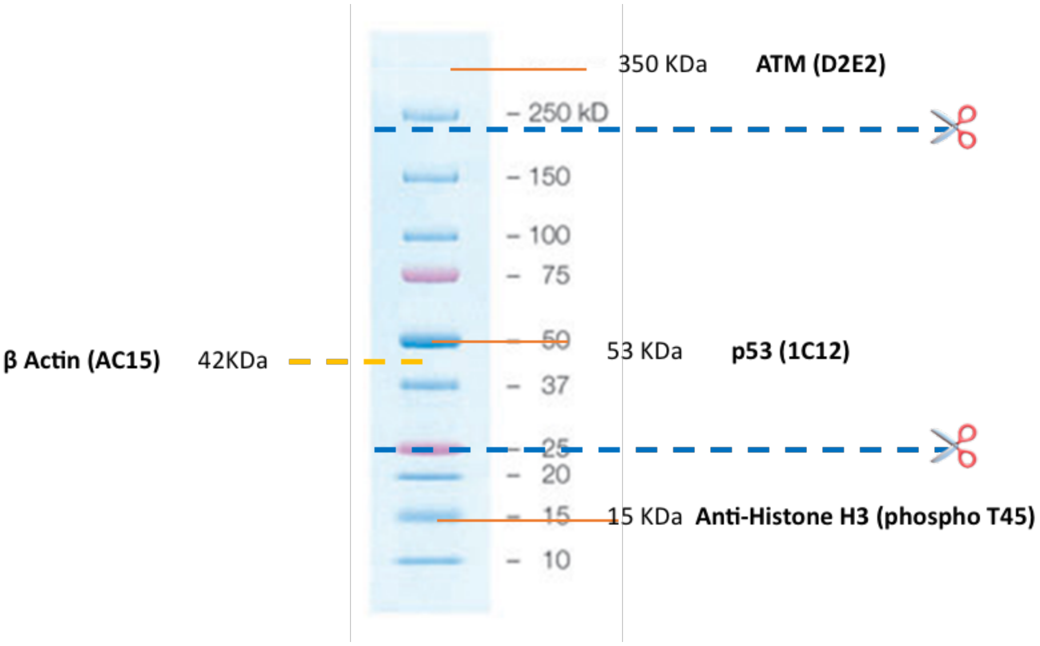
350 KDa ATM (D2E2)
250 kD
150
100
75
50
37
25
20
15
10
53 KDa p53 (1C12)
β Actin (AC15) 42KDa
15 KDa Anti-Histone H3 (phospho T45)

Full length, unedited, original Western Blot images we indicate with red boxes (with dashes) the regions of the original blots used in the main figures.

Complementary western blot images to Figure 1.- In vivo proliferation study of mouse thymocytes after a single or combined radiation regime.

ATM

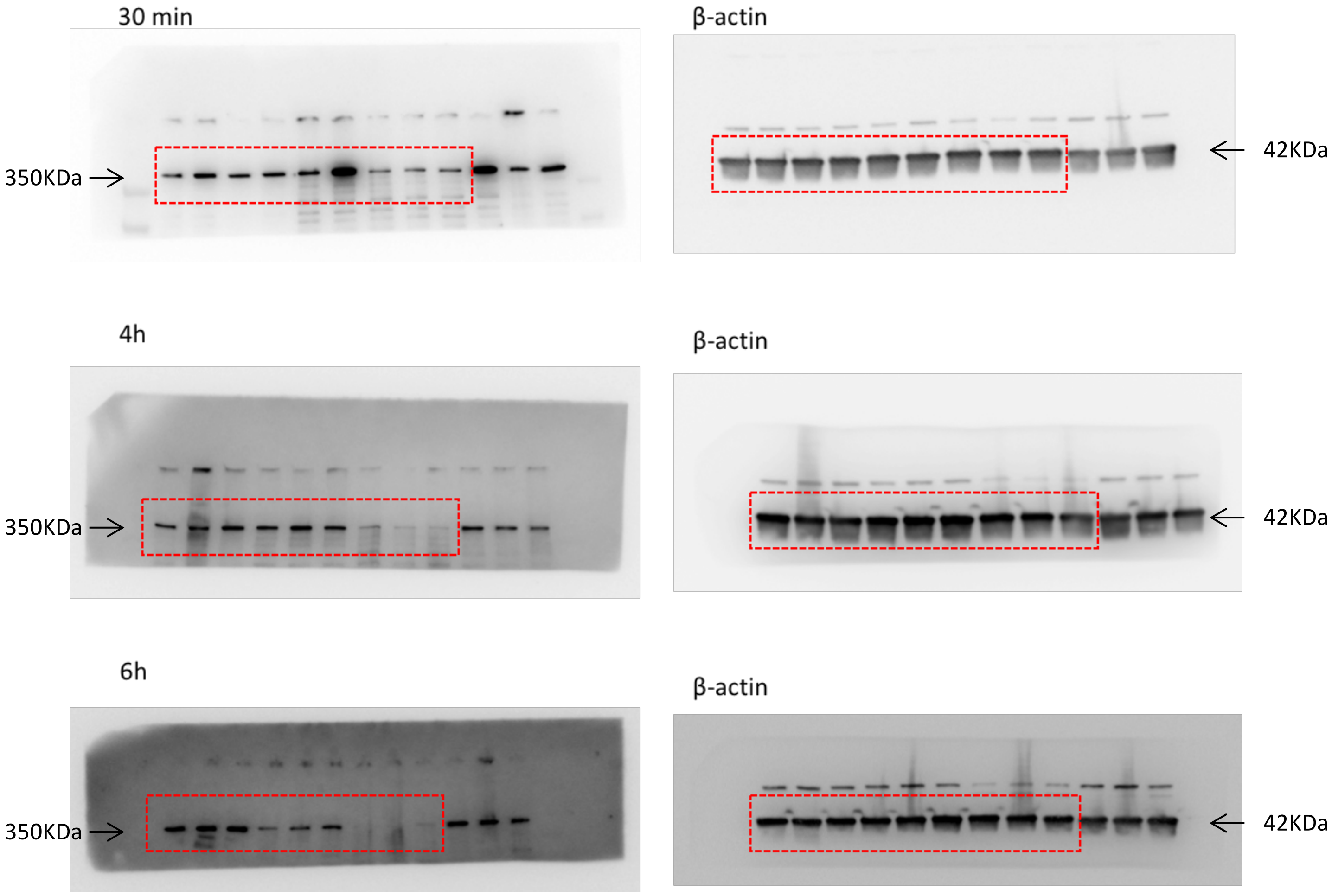

TP53 (tumor protein p53)

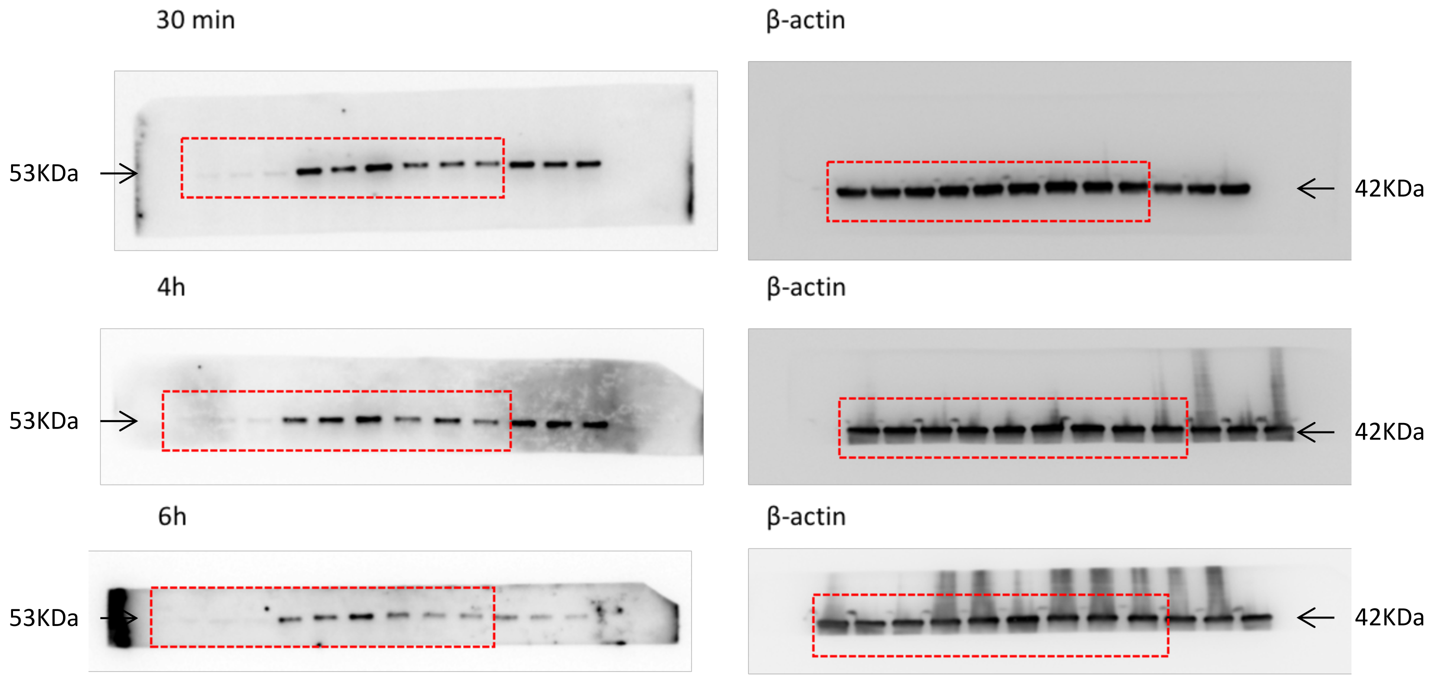

phosphoserine-18-TP53

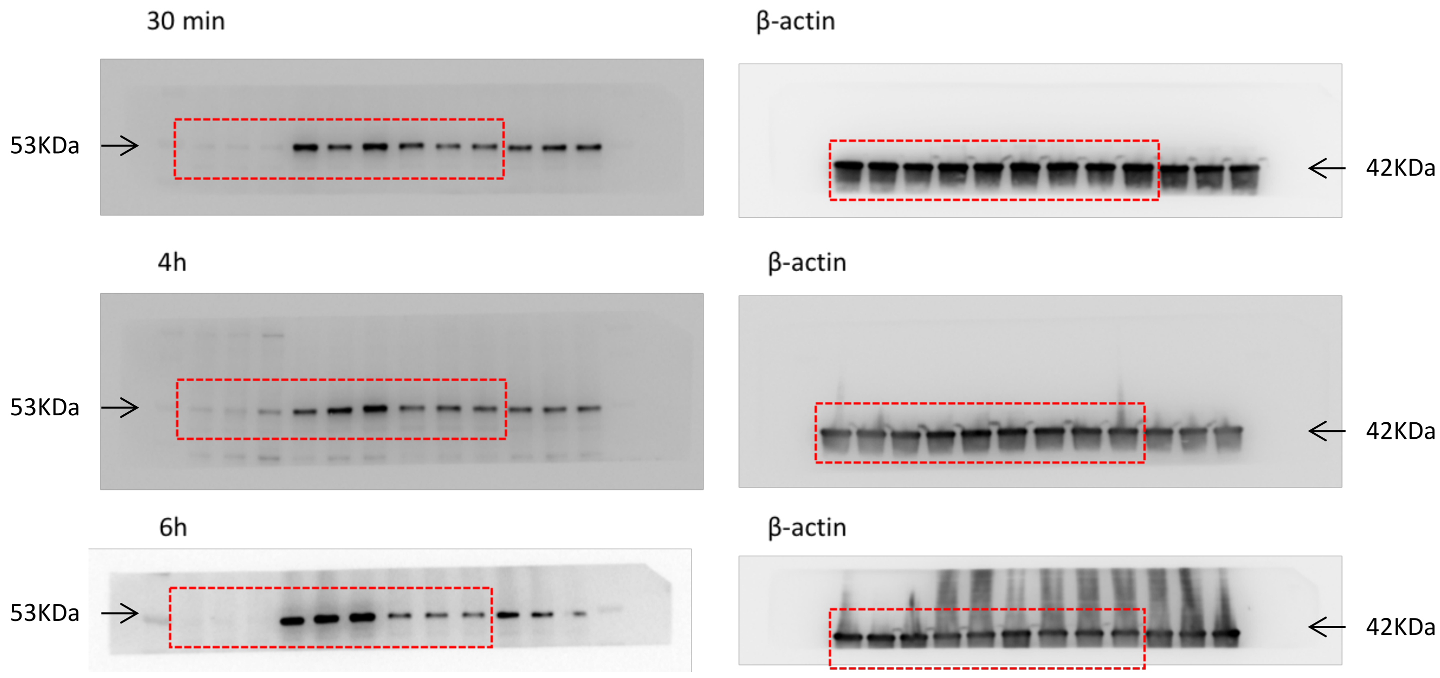

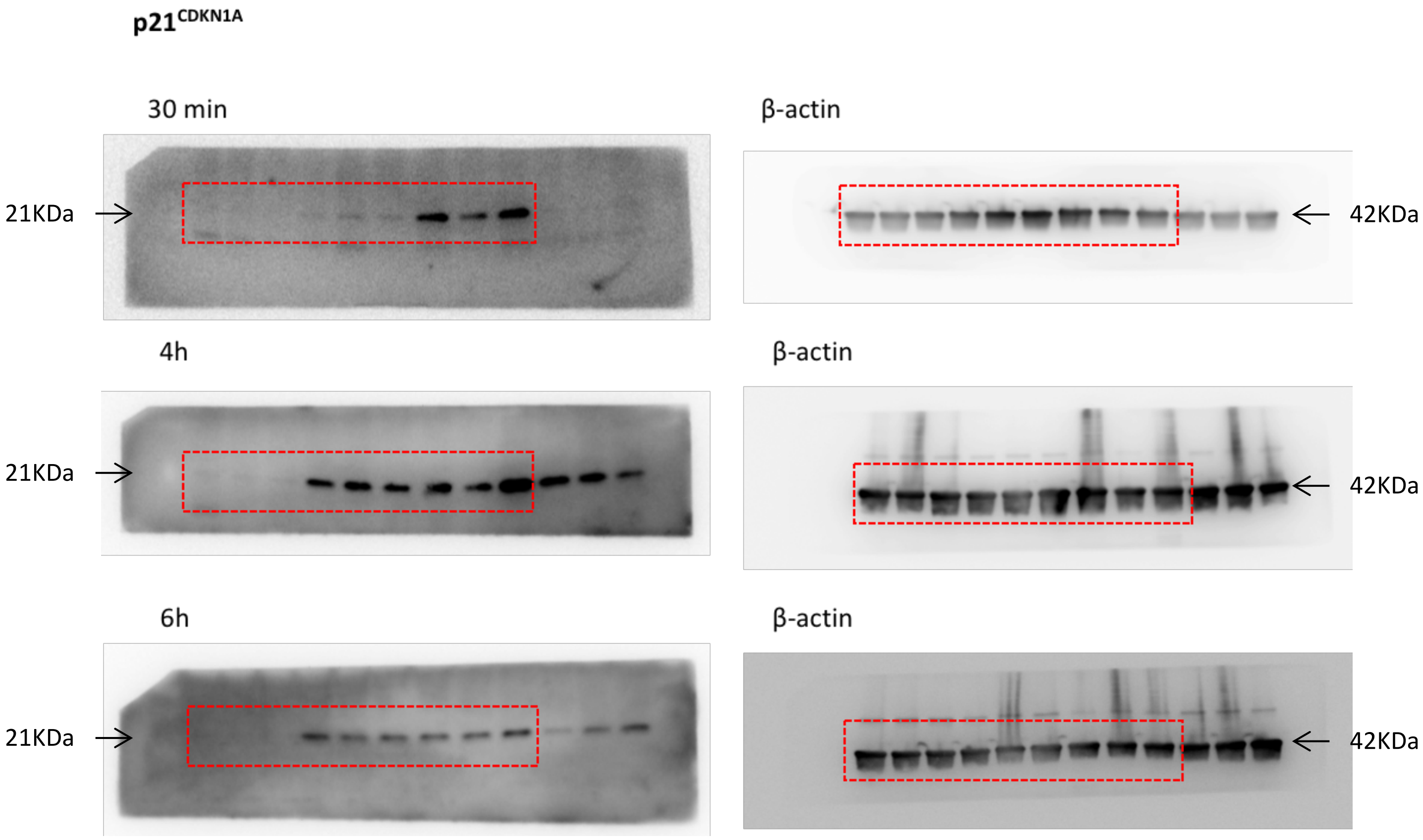

p21CDKN1A
30 min
β-actin
21KDa
42KDa
4h
β-actin
21KDa
42KDa
6h
β-actin
21KDa
42KDa

Complementary western blot images to Figure 2.- Study of the DNA damage response and cell death pathways in thymocytes in vivo after a single or combined radiation scheme.

γH2A.X-Ser139

30 min

15KDa

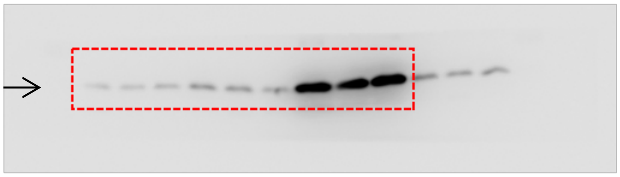

β-actin

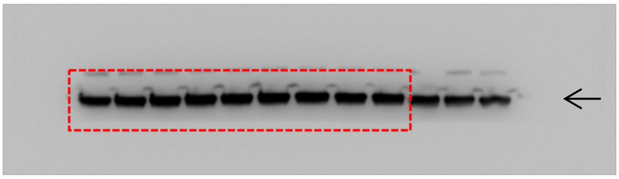

42KDa

4h

15KDa

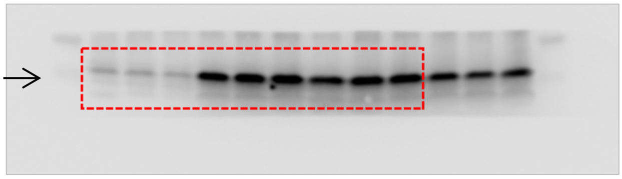

β-actin

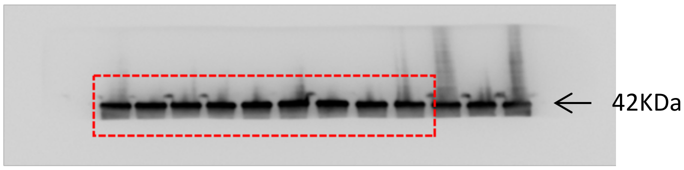

42KDa

6h

15KDa

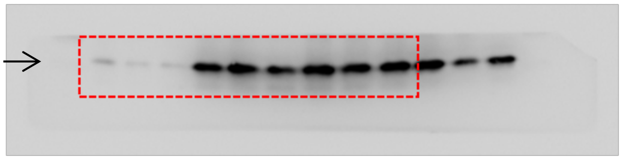

β-actin

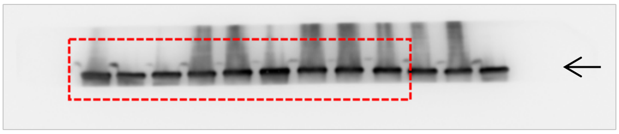

42KDa

Caspase 3 activated subunits

30 min

19KDa
17KDa

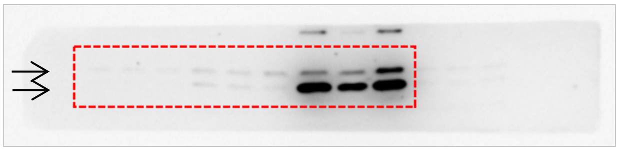

β-actin

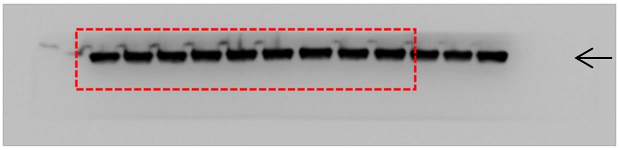

42KDa

4h

19KDa
17KDa

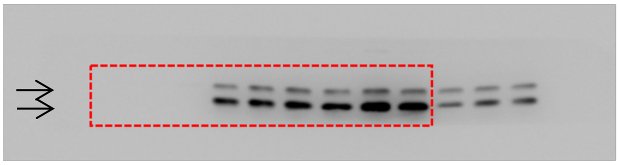

β-actin

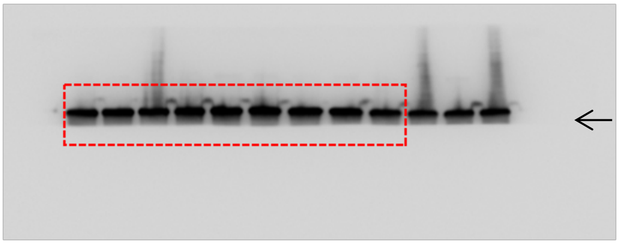

42KDa

6h

19KDa
17KDa

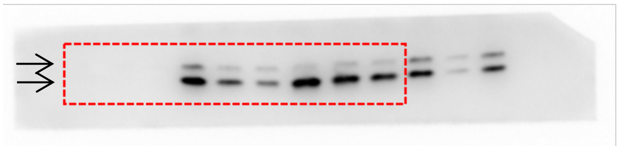

β-actin

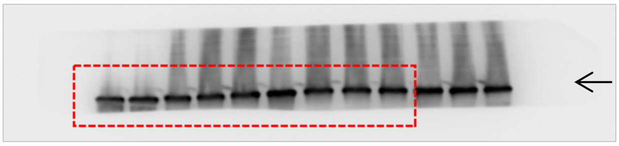

42KDa

Poly(ADP-ribose) polymerase-1 (PARP)

30 min

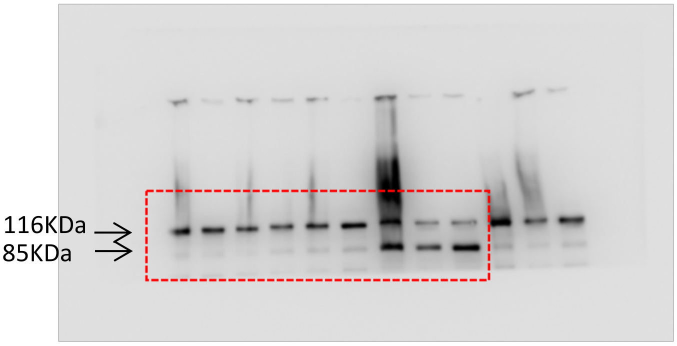

β-actin

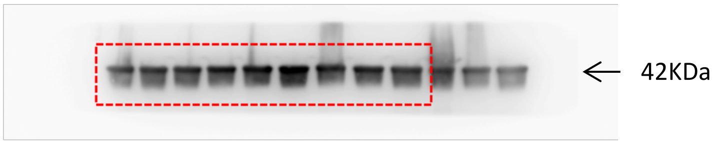

4h

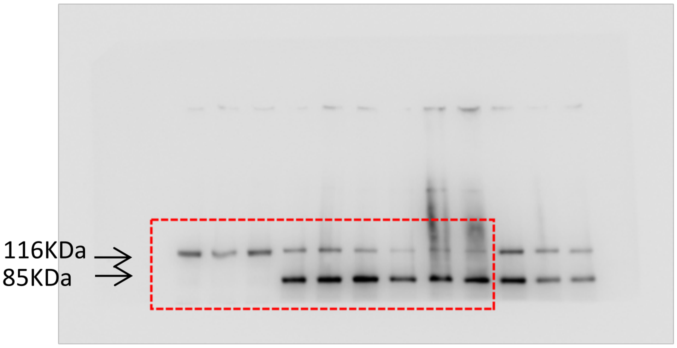

β-actin

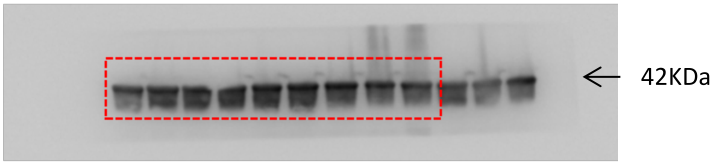

6h

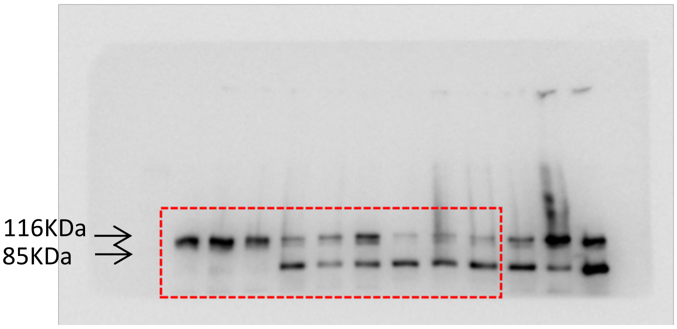

β-actin

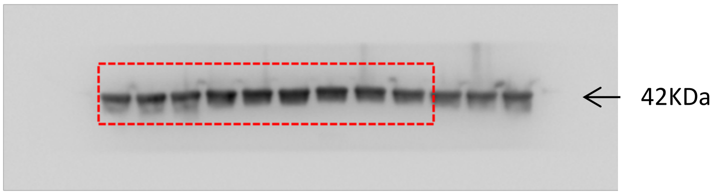

PKCδ full length (78 kDa) and its catalytic fragment (41 kDa).

30 min

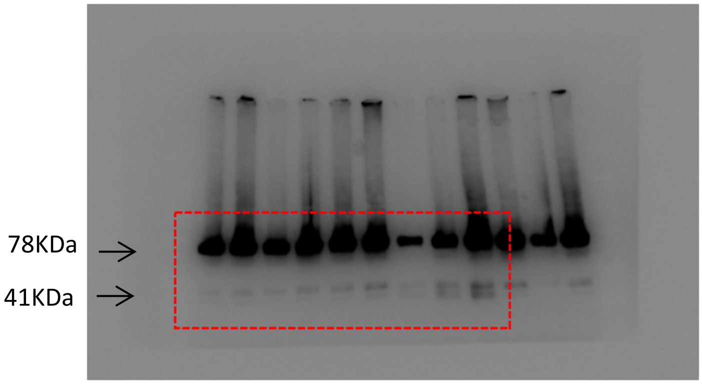

β-actin

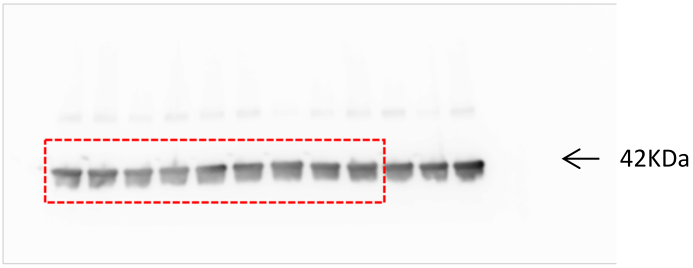

4h

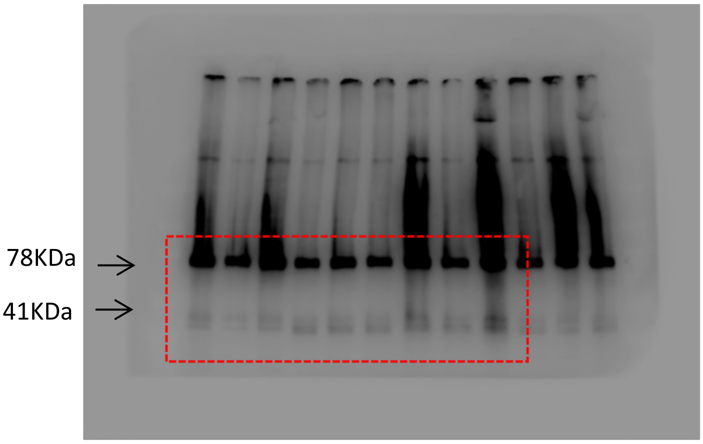

β-actin

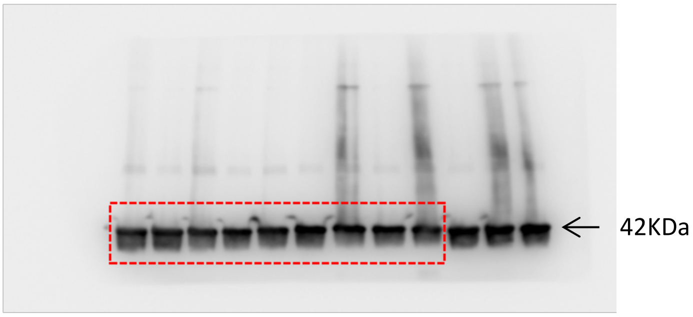

6h

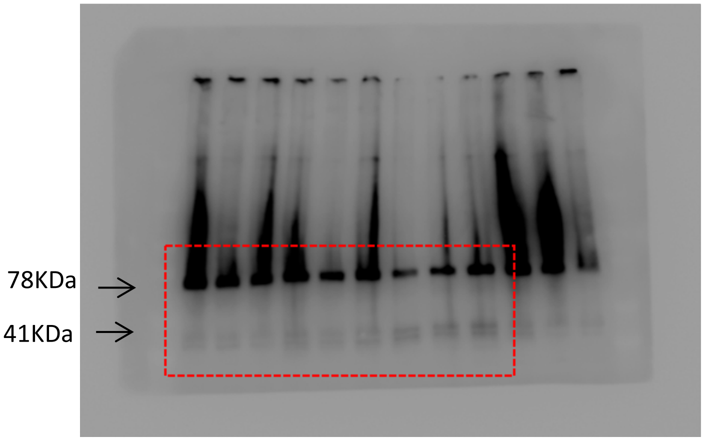

β-actin

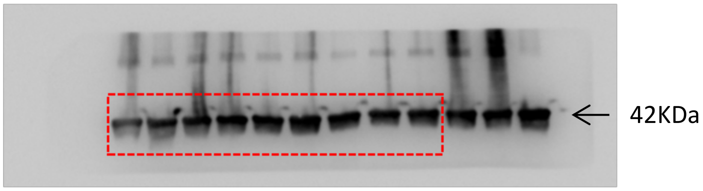

Phosphorylation of H3T45 (H3T45ph)

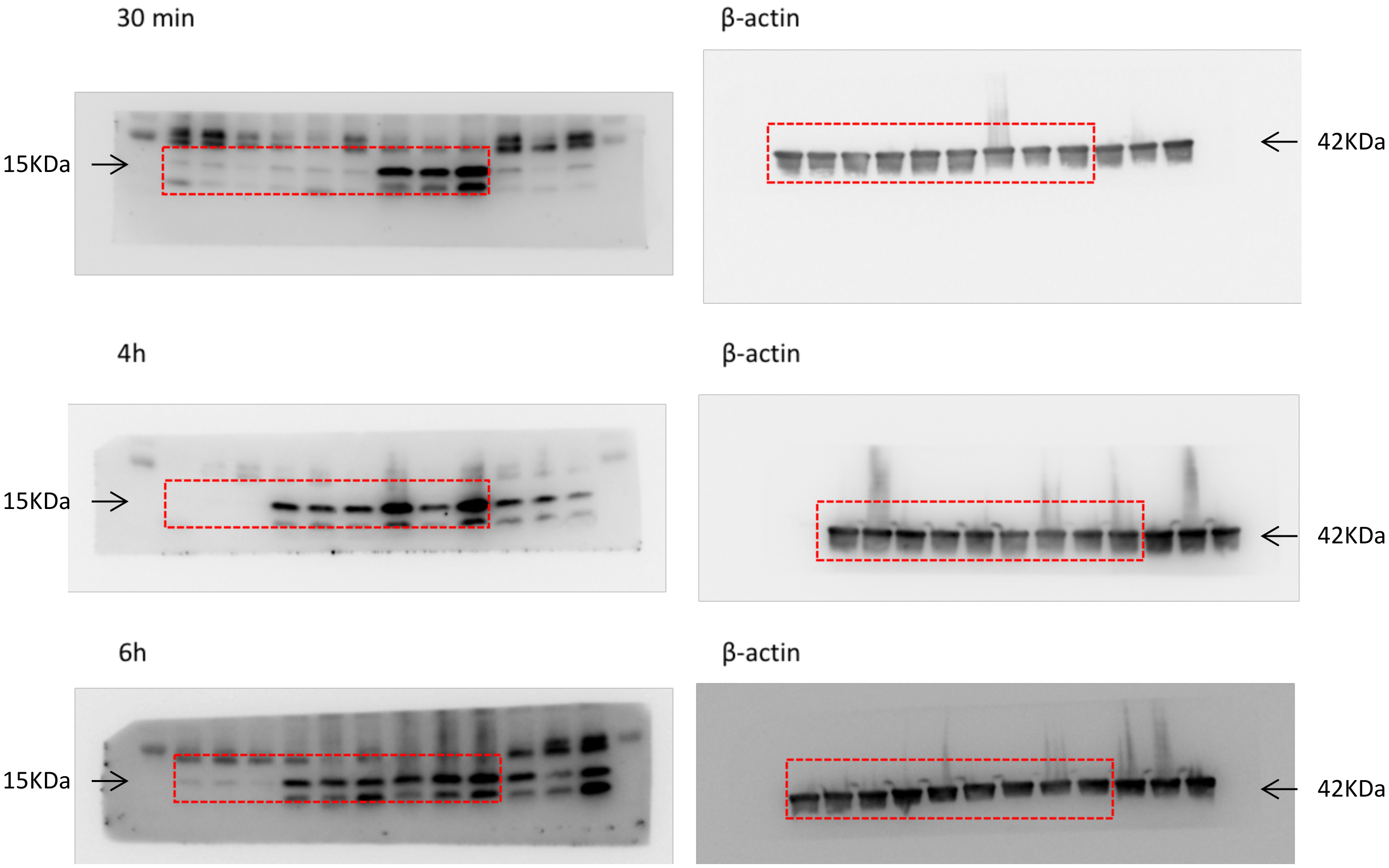

SLC7A11

30 min

35KDa →

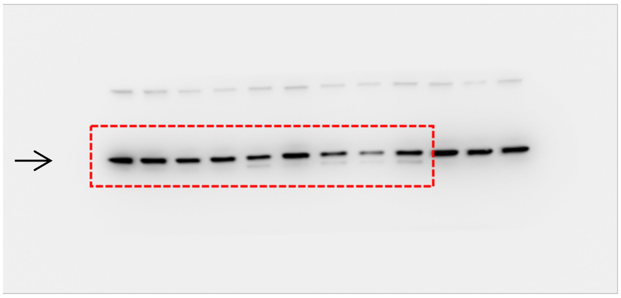

β-actin

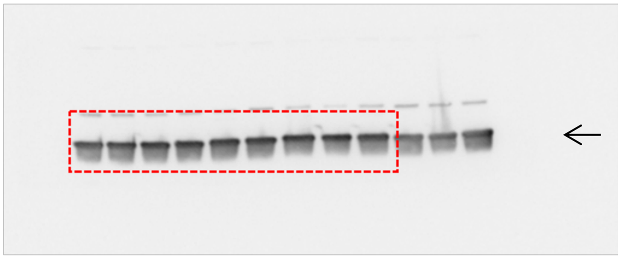

← 42KDa

4h

35KDa →

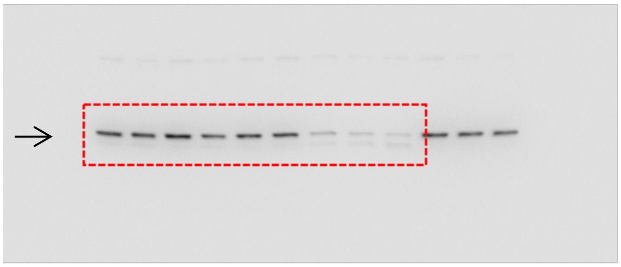

β-actin

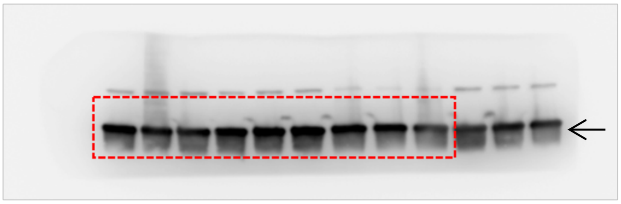

← 42KDa

6h

35KDa →

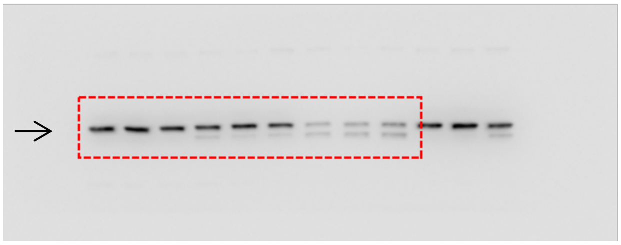

β-actin

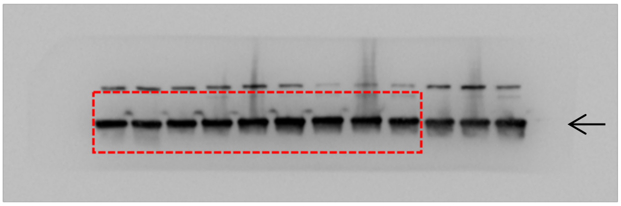

← 42KDa

GPX4

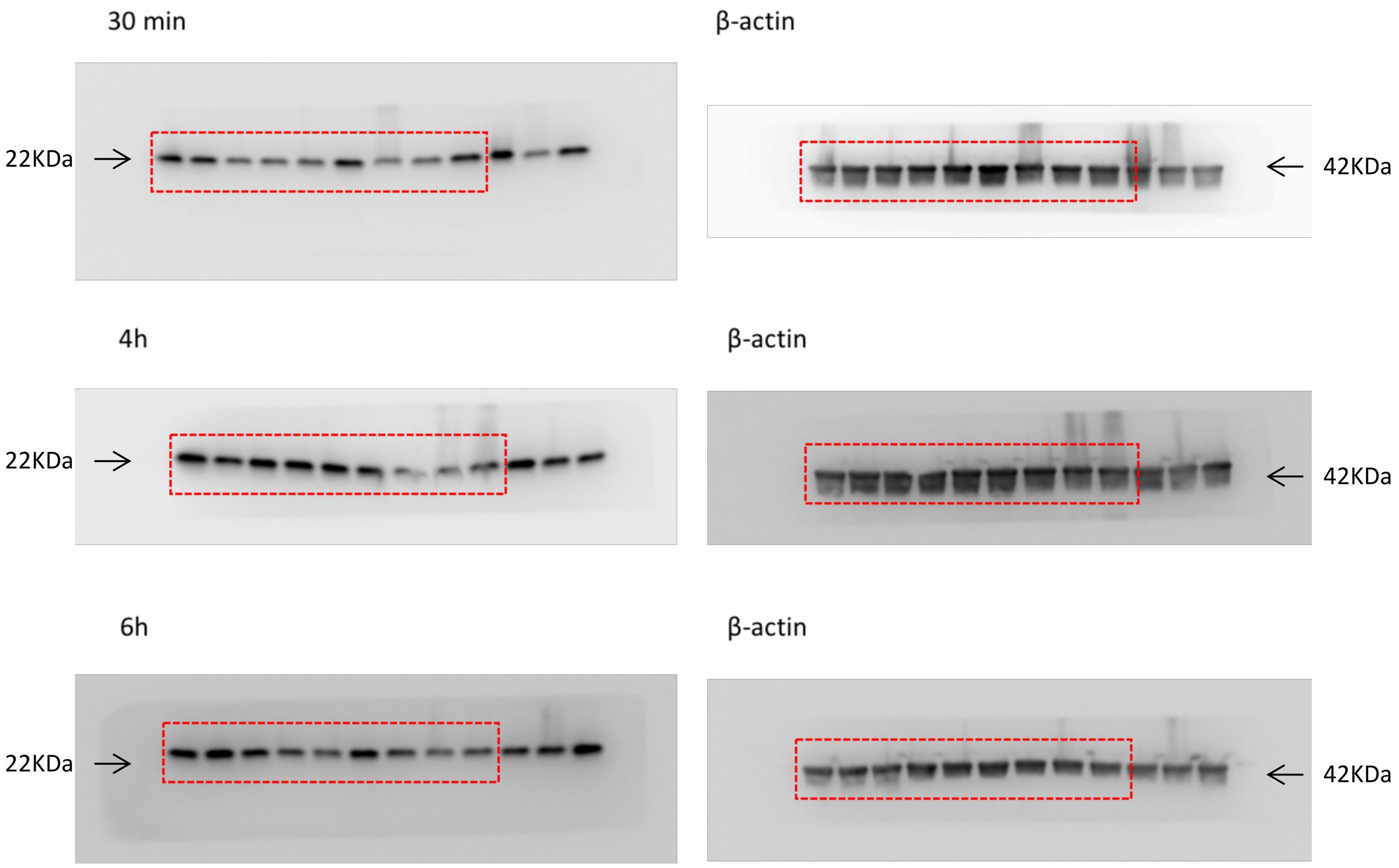
30 min
β-actin
22KDa
42KDa
4h
β-actin
22KDa
42KDa
6h
β-actin
22KDa
42KDa

Complementary western blot images to Figure 3.- Study of the response of chromatin remodelers to a combined radiation regime versus control or single irradiated mouse thymocytes in vivo.

DNMT1

30 min

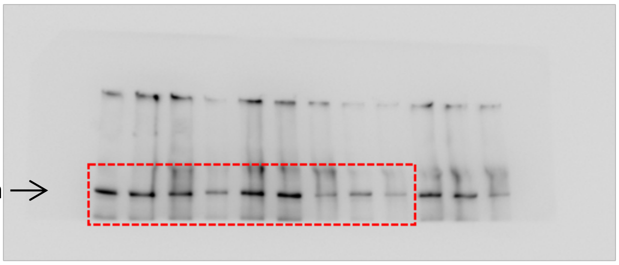

200KDa →

β-actin

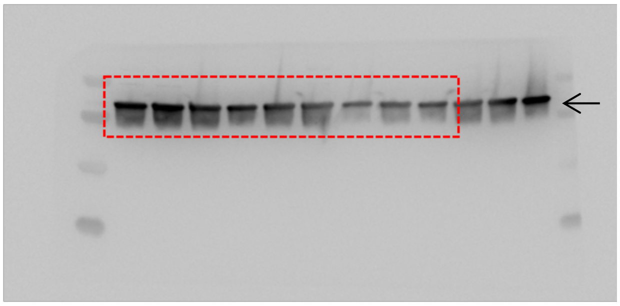

← 42KDa

4h

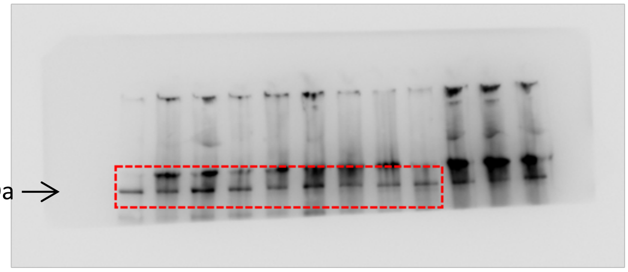

200KDa →

β-actin

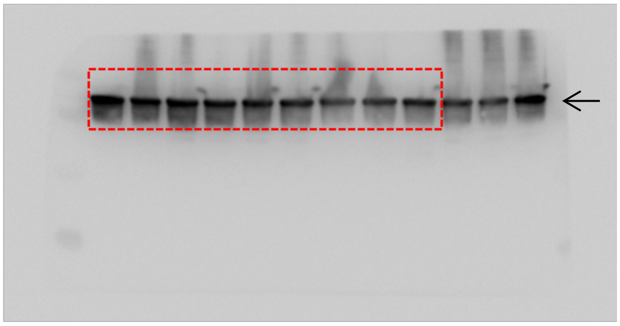

← 42KDa

6h

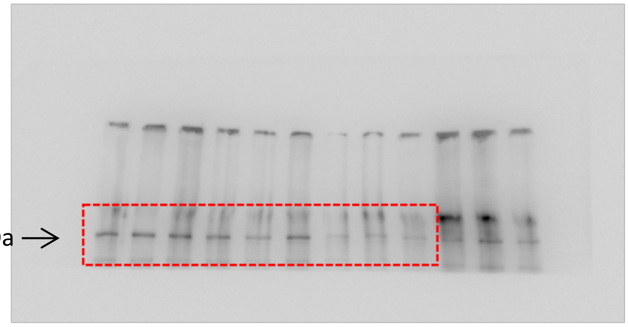

200KDa →

β-actin

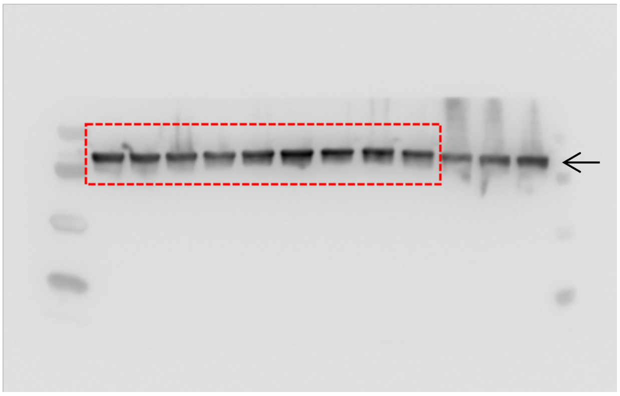

← 42KDa

DNMT2

30 min

β-actin

55KDa →

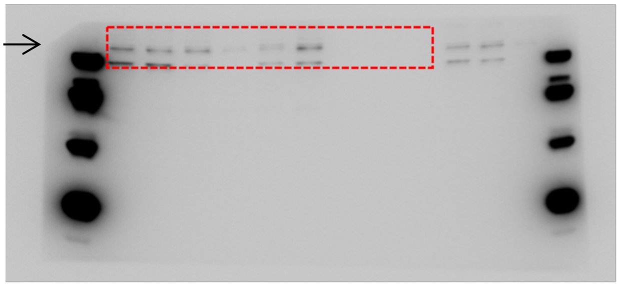

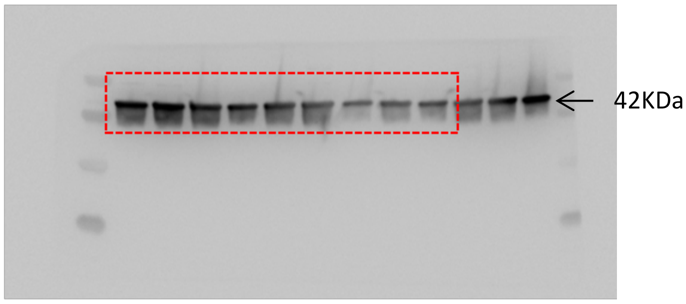

← 42KDa

4h

β-actin

55KDa →

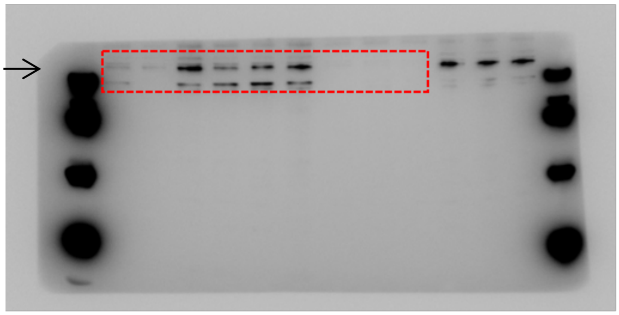

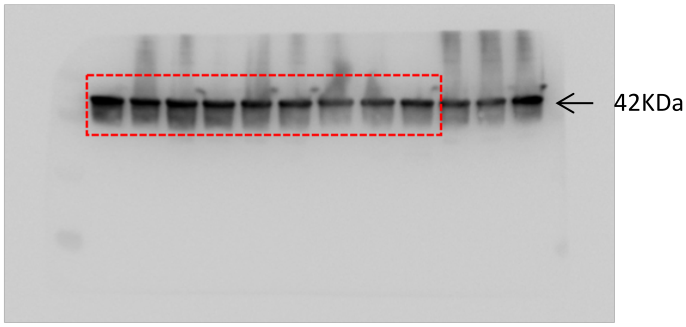

← 42KDa

6h

β-actin

55KDa →

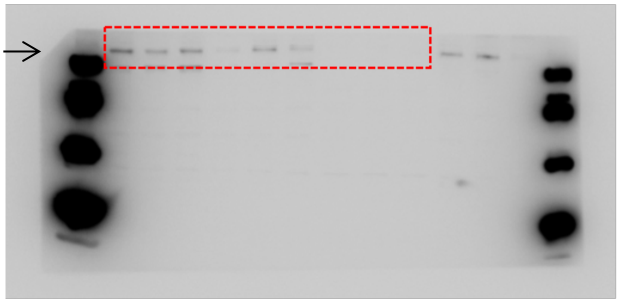

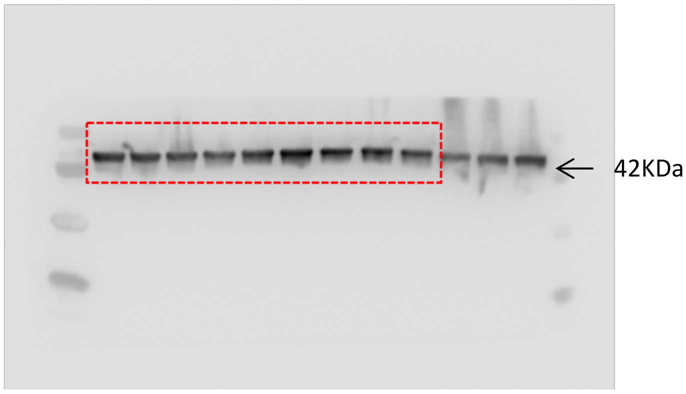

← 42KDa

DNMT3A

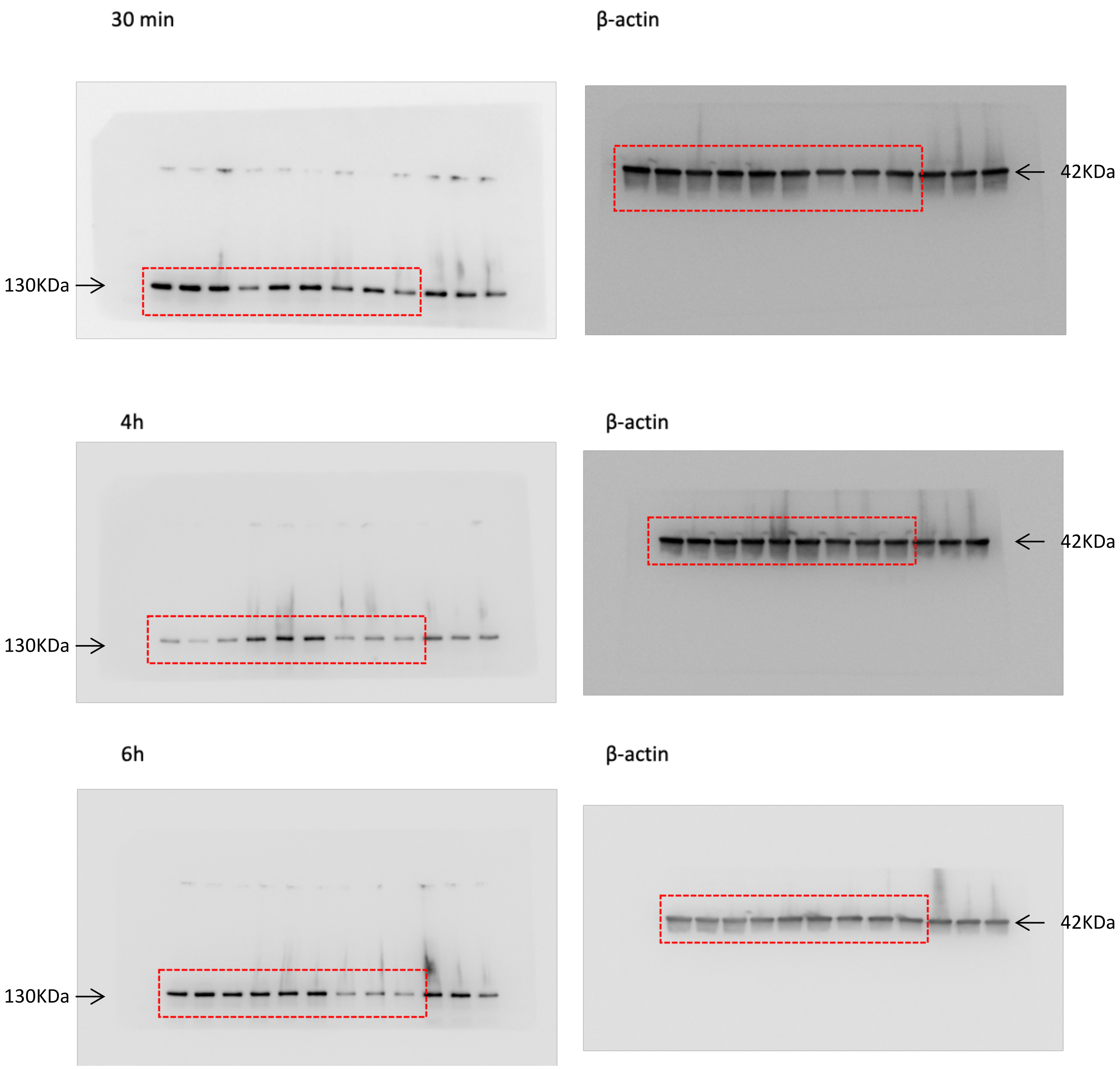

30 min
β-actin
130KDa
42KDa
4h
β-actin
130KDa
42KDa
6h
β-actin
130KDa
42KDa

DNMT3B

30 min

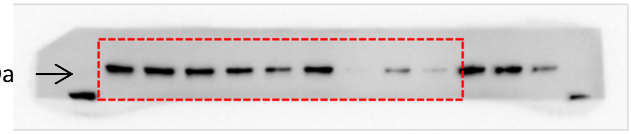

96KDa

β-actin

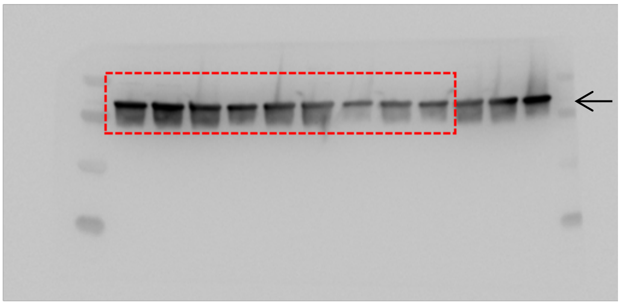

42KDa

4h

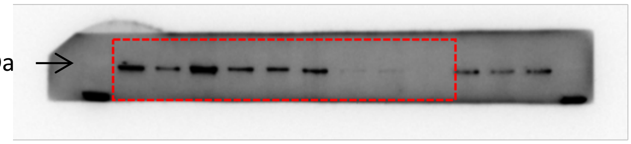

96KDa

β-actin

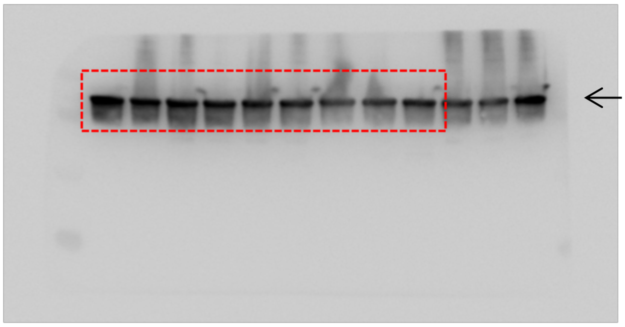

42KDa

6h

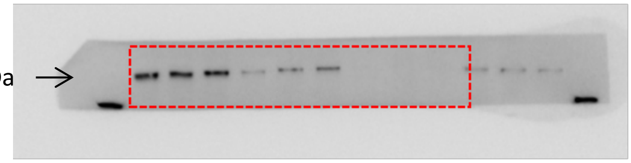

96KDa

β-actin

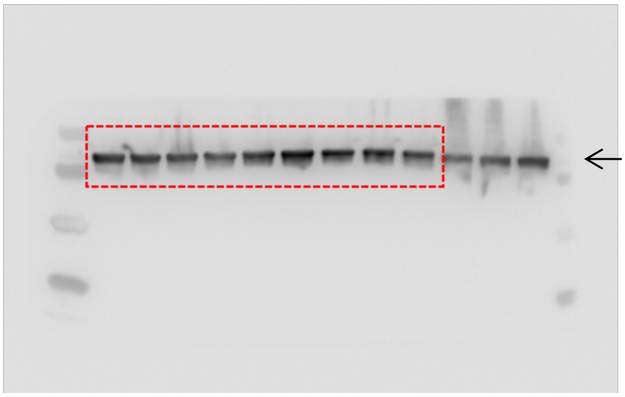

42KDa

DNMT3L

30 min

49KDa

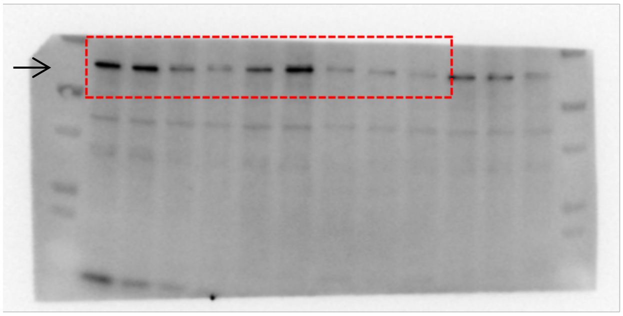

β-actin

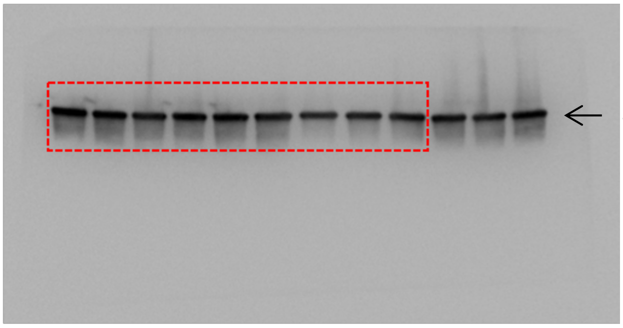

42KDa

4h

49KDa

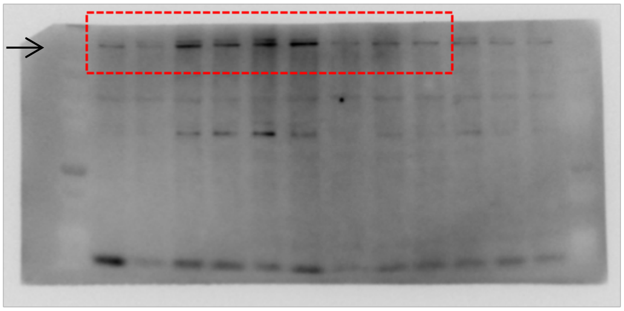

β-actin

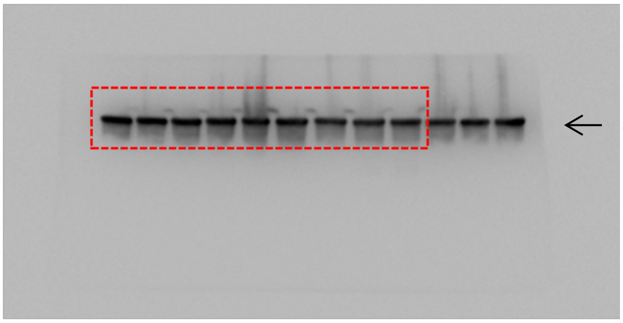

42KDa

6h

49KDa

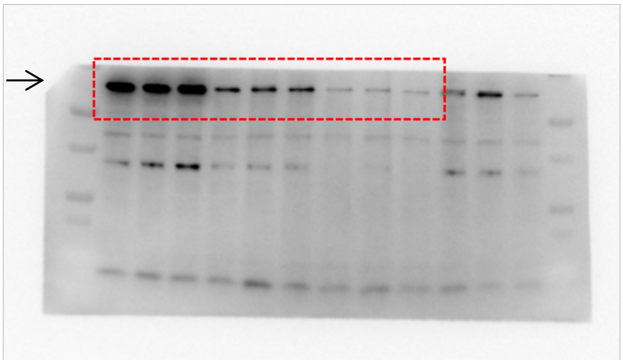

β-actin

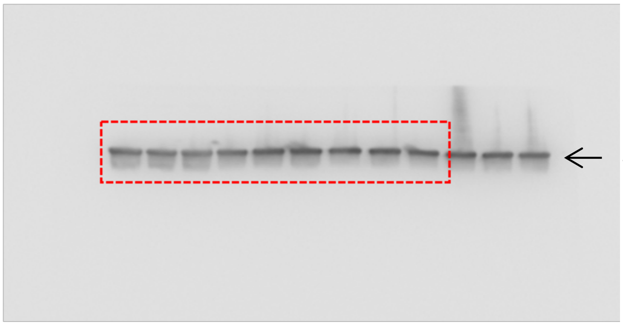

42KDa

MBD4

30 min

60/65KDa →

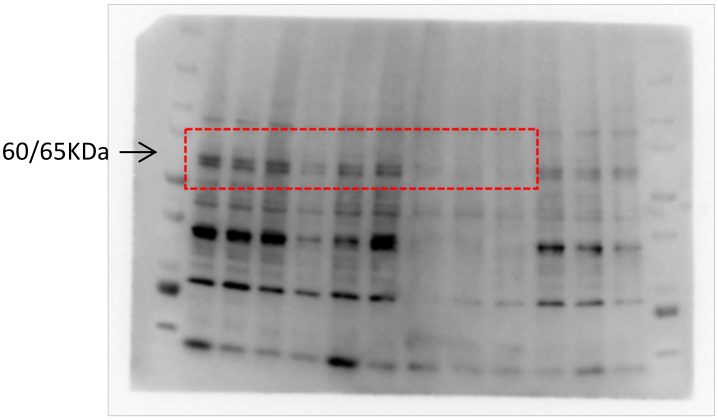

β-actin

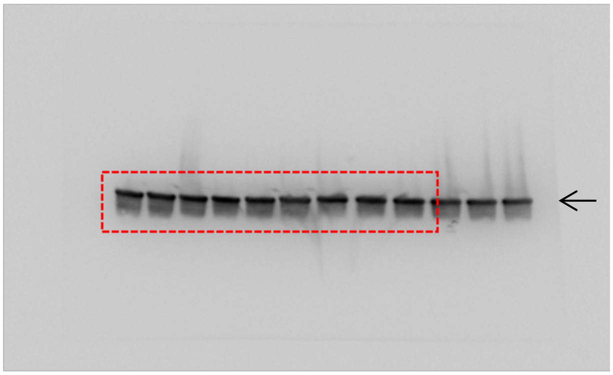

← 42KDa

4h

60/65KDa →

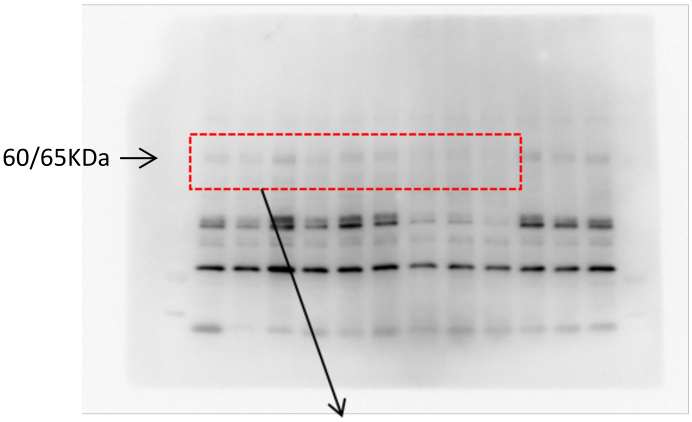

β-actin

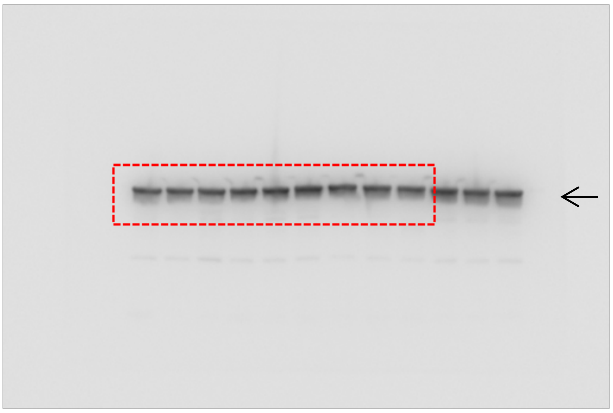

← 42KDa

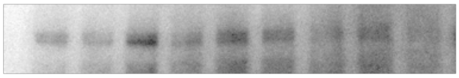

autoexposure after cropped

6h

60/65KDa →

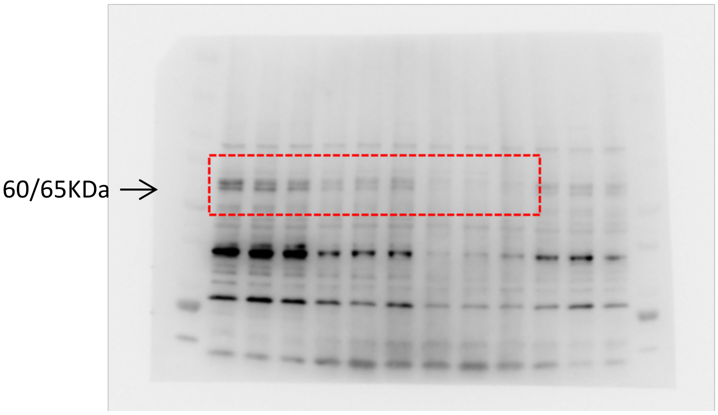

β-actin

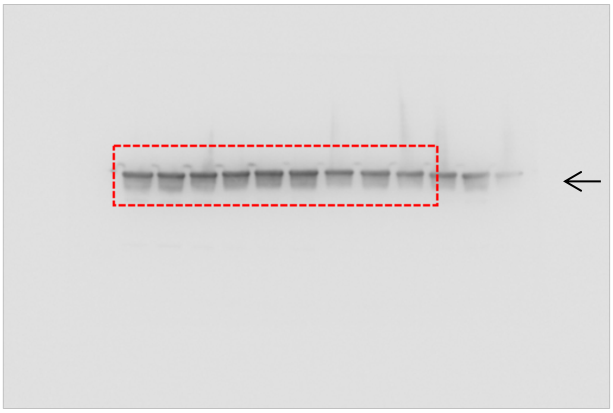

← 42KDa

TDG

30 min

55/65KDa

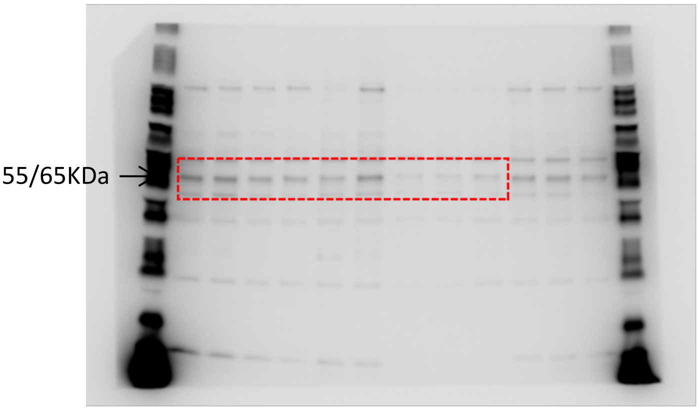

β-actin

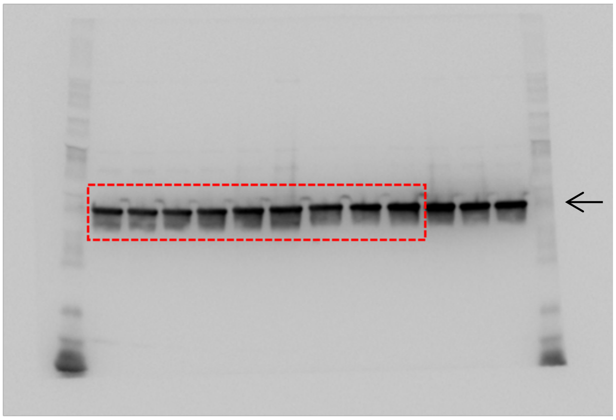

42KDa

4h

55/65KDa

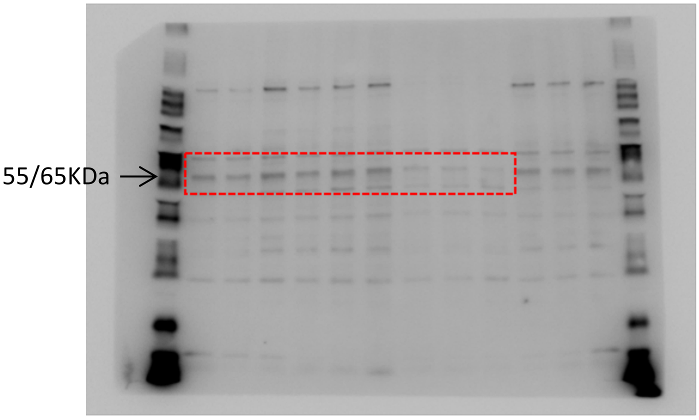

β-actin

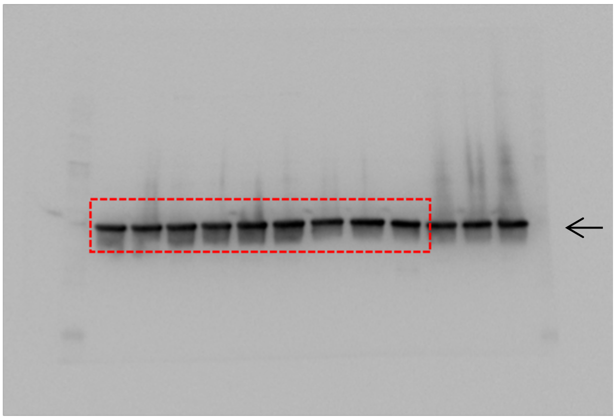

42KDa

6h

55/65KDa

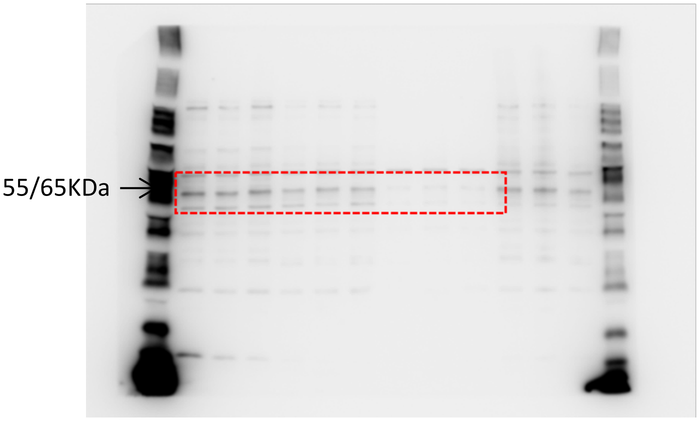

β-actin

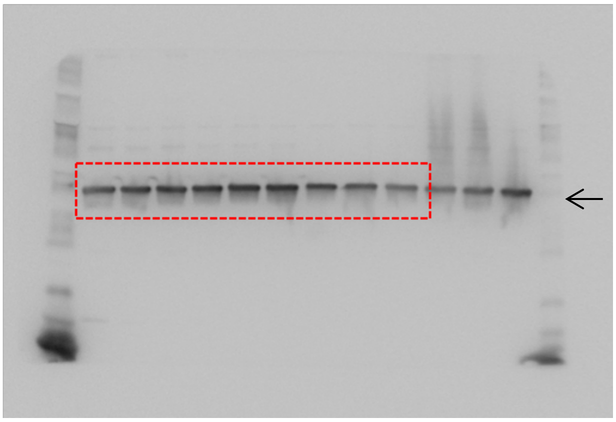

42KDa

Complementary western blot images to Figure 4.- Study of the remnant radiation response 6 hours after exposure to the priming dose (0.0075 Gy) in mice in vivo.

ATM

350KDa →

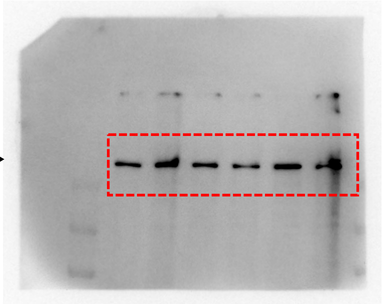

β-actin

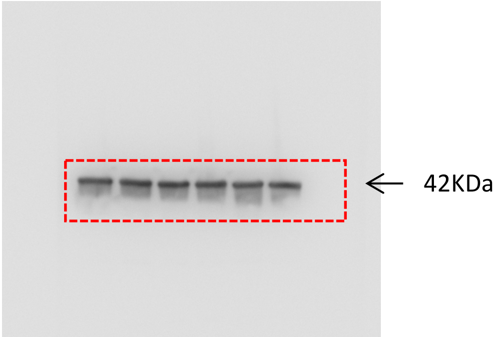

← 42KDa

TP53

53KDa →

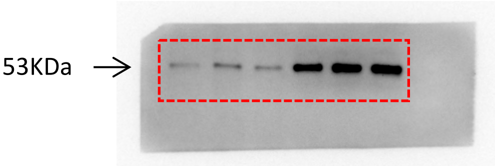

β-actin

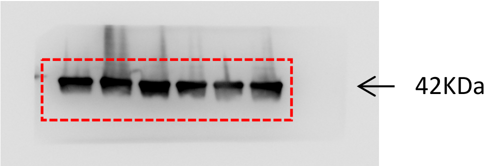

← 42KDa

phosphoserine-18-TP53

53KDa →

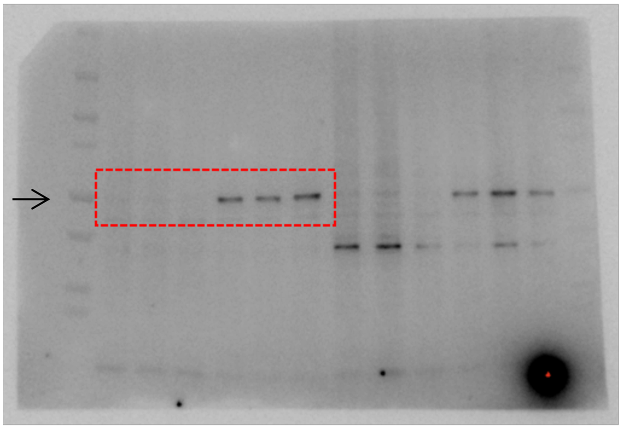

β-actin

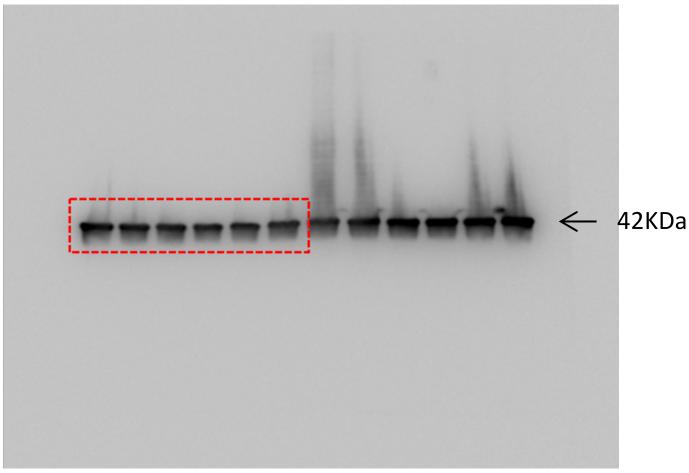

← 42KDa

p21[CDKN1A]

21KDa →

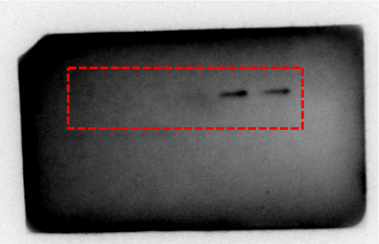

β-actin

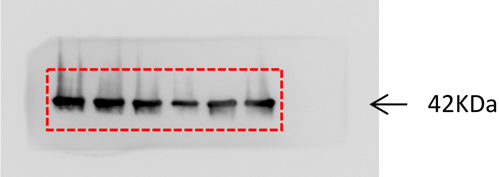

← 42KDa

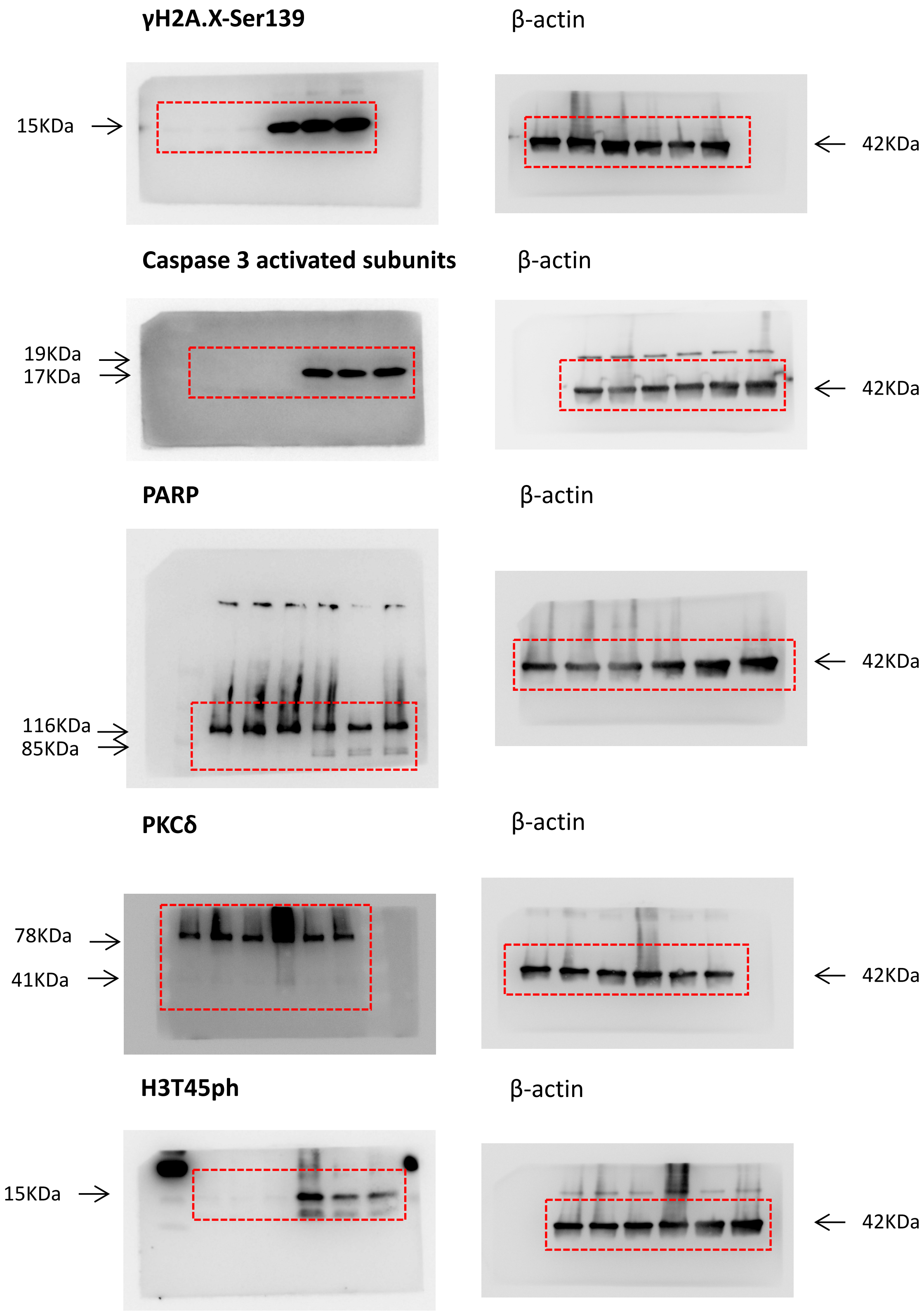
γH2A.X-Ser139
β-actin
15KDa
42KDa
Caspase 3 activated subunits
β-actin
19KDa
17KDa
42KDa
PARP
β-actin
116KDa
85KDa
42KDa
PKCδ
β-actin
78KDa
41KDa
42KDa
H3T45ph
β-actin
15KDa
42KDa

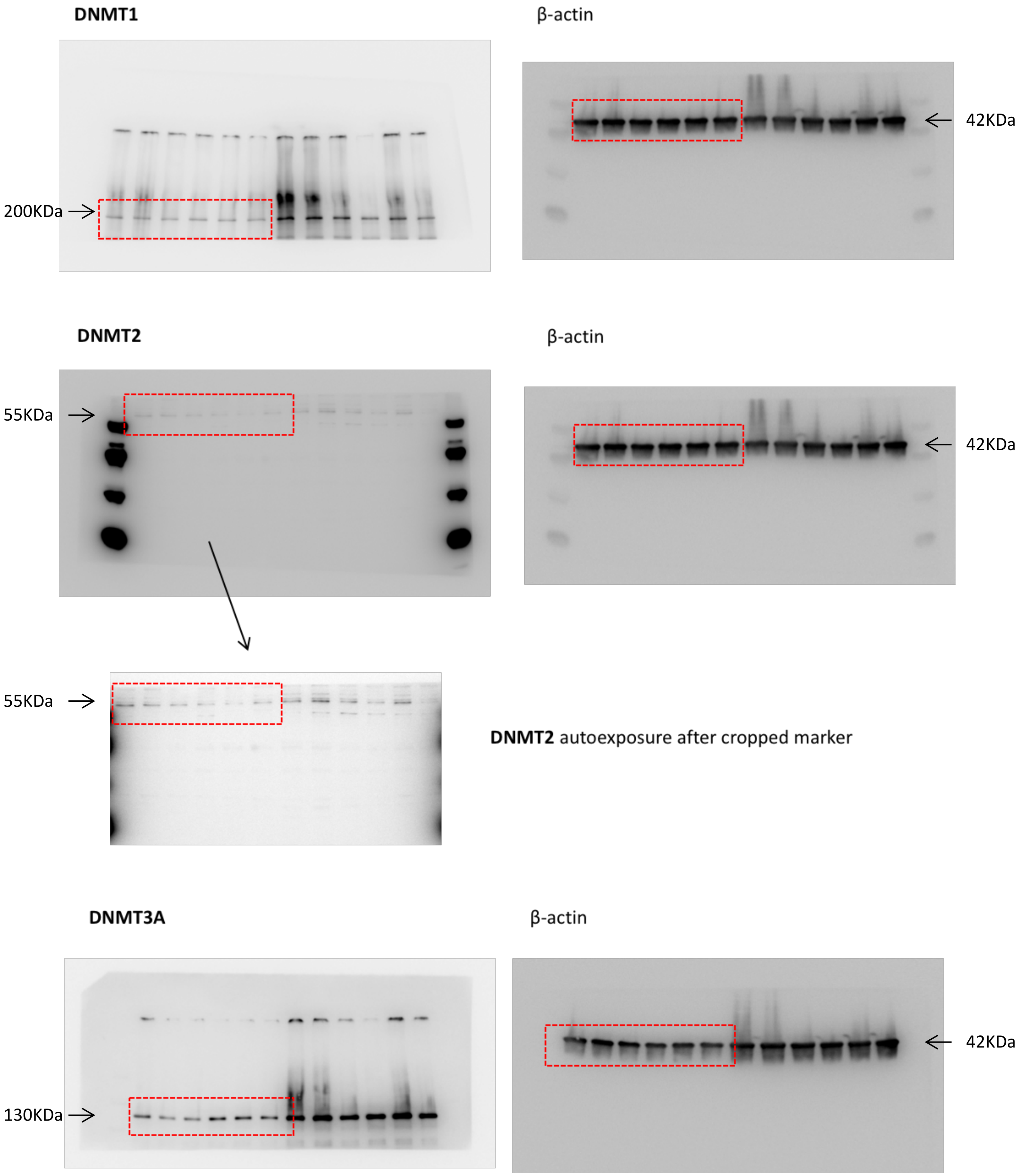
DNMT1
β-actin
200KDa
42KDa
DNMT2
β-actin
55KDa
42KDa
55KDa
DNMT2 autoexposure after cropped marker
DNMT3A
β-actin
130KDa
42KDa

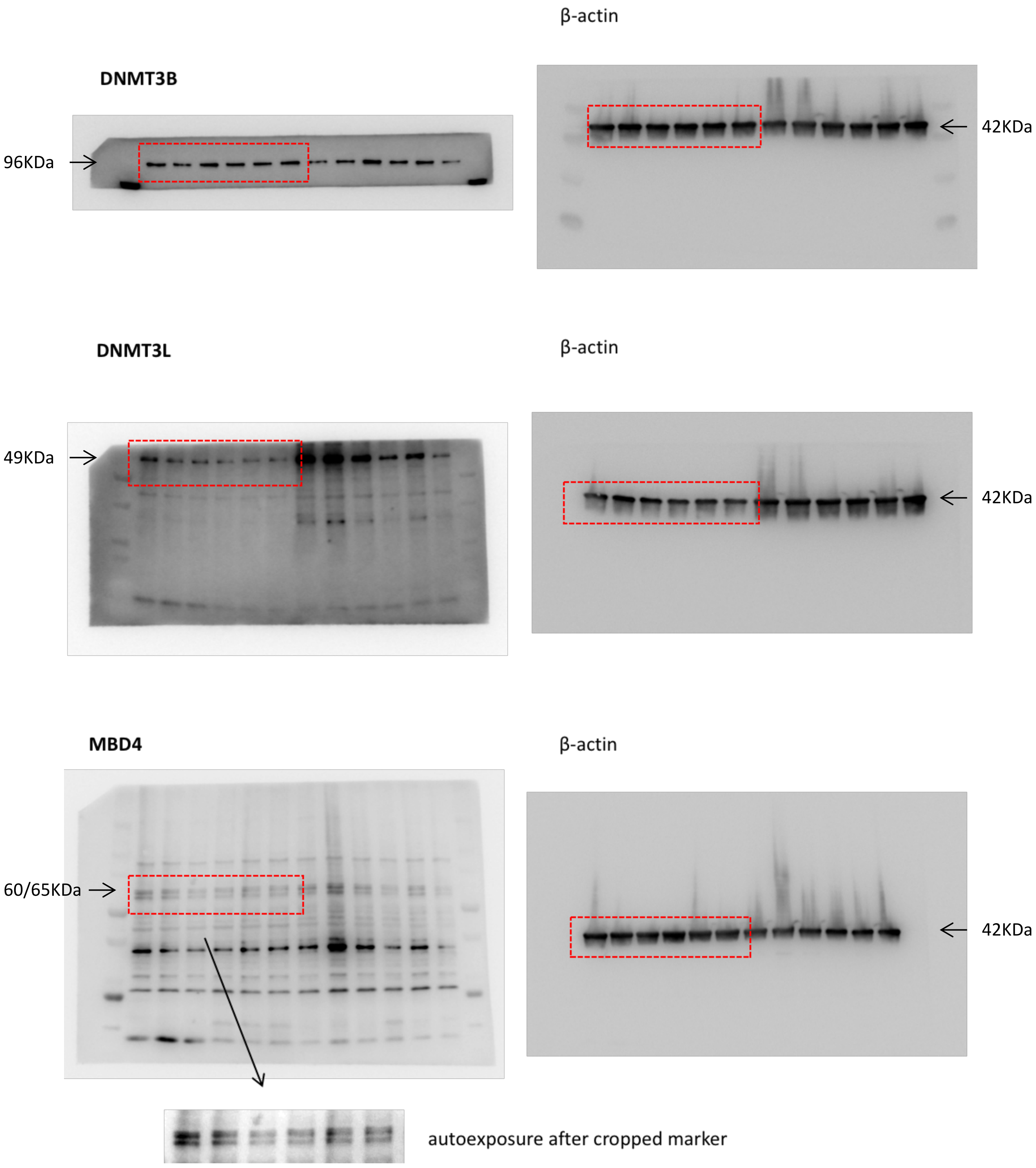

DNMT3B
β-actin
96KDa
42KDa
DNMT3L
β-actin
49KDa
42KDa
MBD4
β-actin
60/65KDa
42KDa
autoexposure after cropped marker

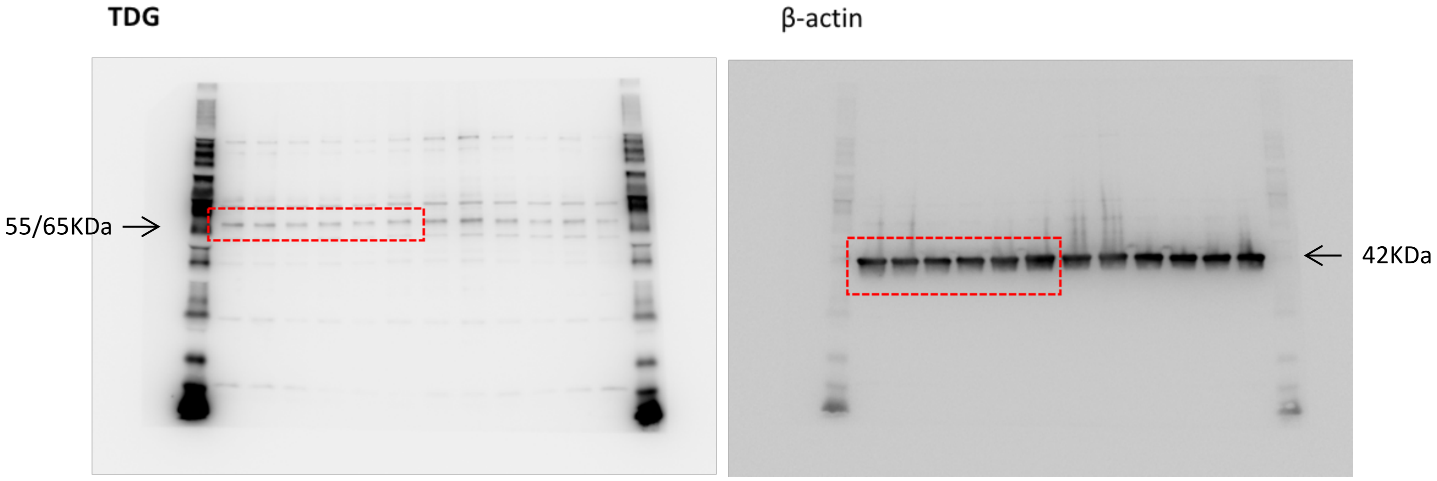

Supplementary Figure 2: Ferroptosis markers study 6 hours after the priming dose in mice.

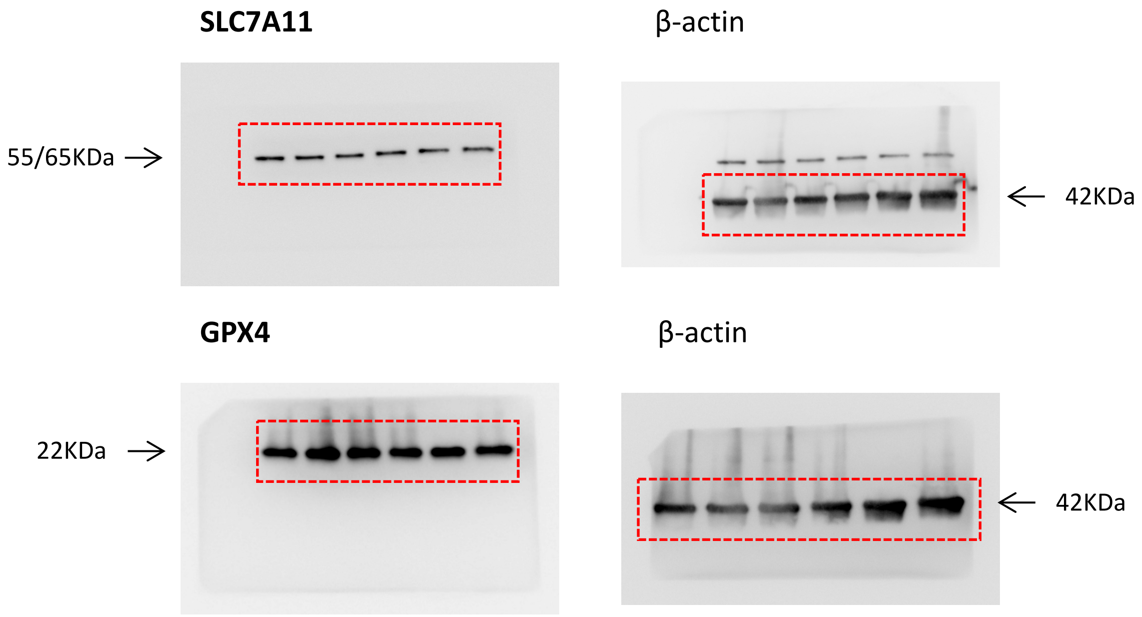

Complementary western blot images to Figure 5.- *Ex vivo response to a combined regime in human thymocytes.*

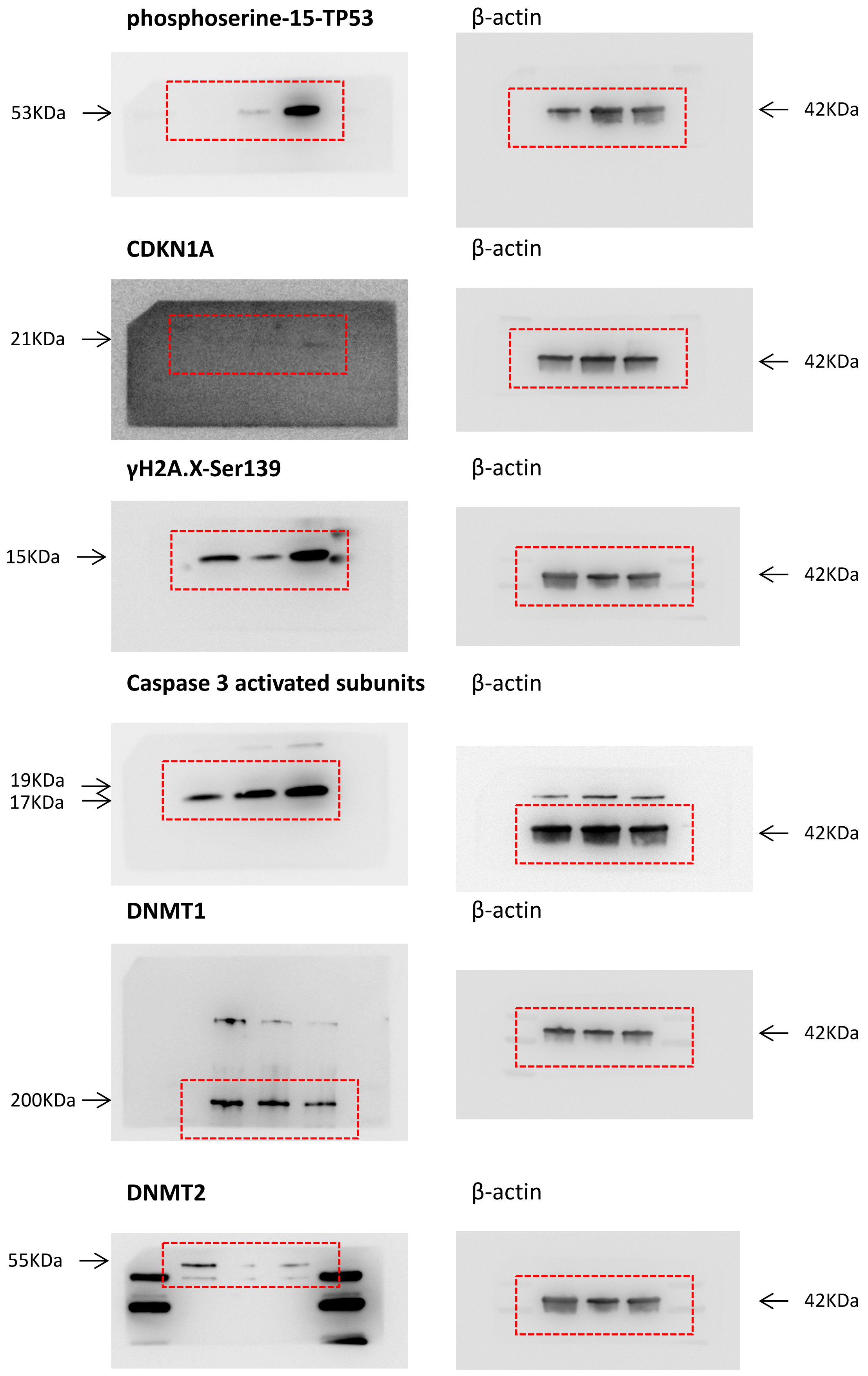

DNMT 3A

130KDa →

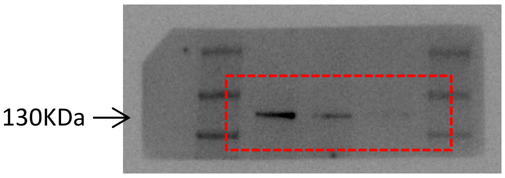

β-actin

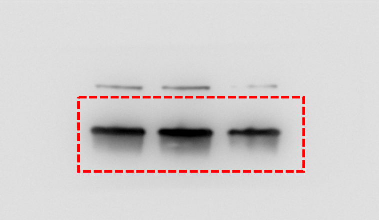

← 42KDa

TDG

55/65KDa →

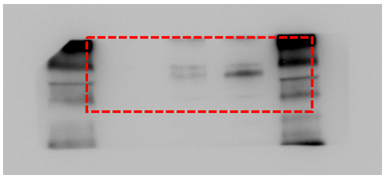

β-actin

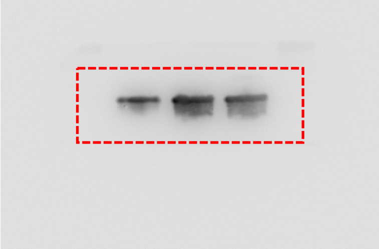

← 42KDa

Replicates (n2) ***Ex vivo response to a combined regime in human thymocytes.***

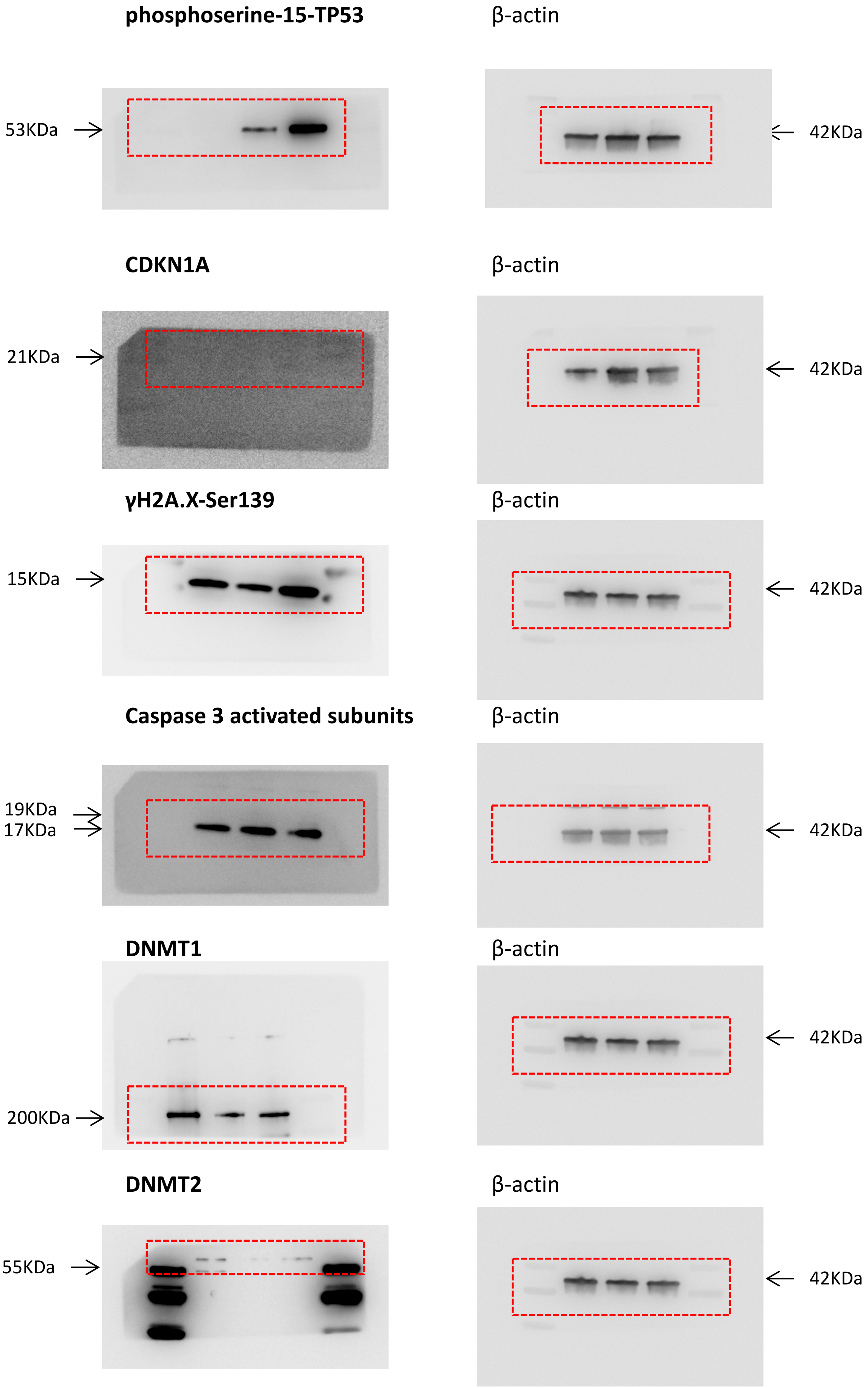

DNMT 3A

130KDa →

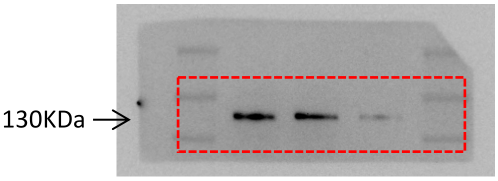

β-actin

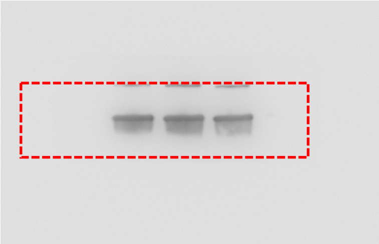

← 42KDa

TDG

55/65KDa →

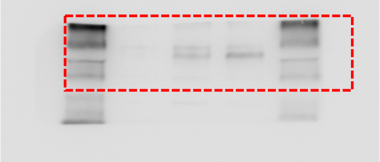

β-actin

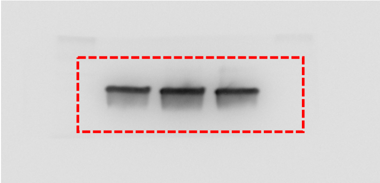

← 42KDa

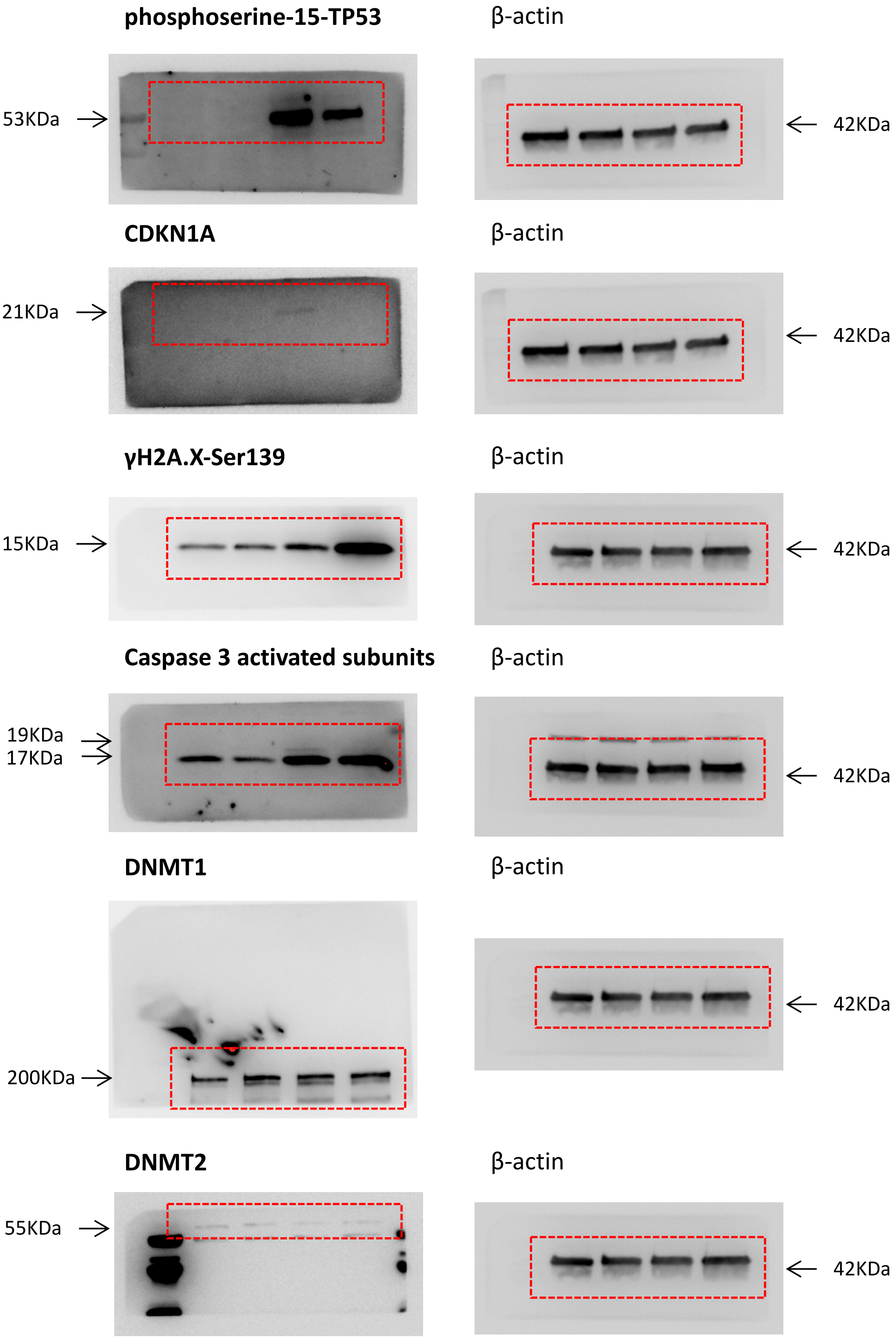
phosphoserine-15-TP53
β-actin
53KDa
42KDa
CDKN1A
β-actin
21KDa
42KDa
γH2A.X-Ser139
β-actin
15KDa
42KDa
Caspase 3 activated subunits
β-actin
19KDa
17KDa
42KDa
DNMT1
β-actin
200KDa
42KDa
DNMT2
β-actin
55KDa
42KDa

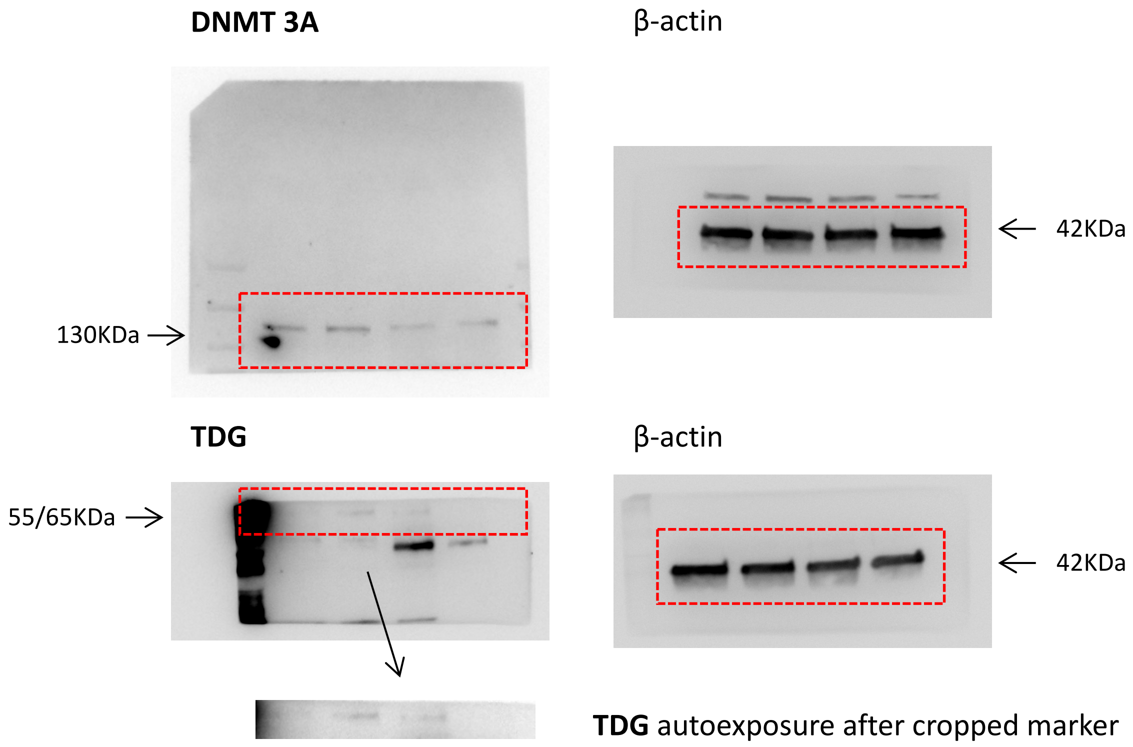

DNMT 3A
β-actin
130KDa
42KDa
TDG
β-actin
55/65KDa
42KDa
TDG autoexposure after cropped marker

Supplementary Figure 4: ***Ex vivo radio-adaptative response in human thymocytes.***

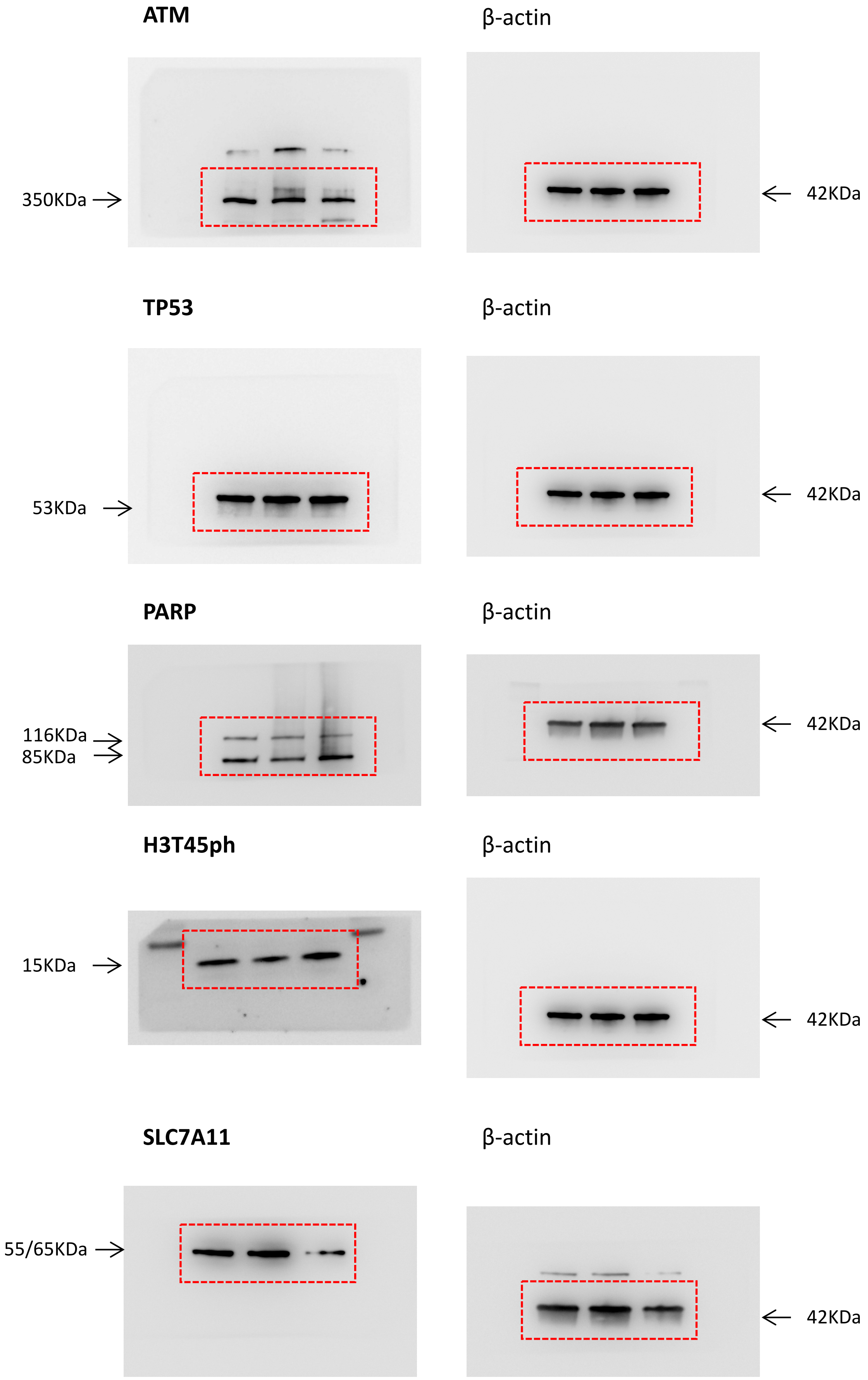

Replicates n2 ***Ex vivo radio-adaptative response in human thymocytes***

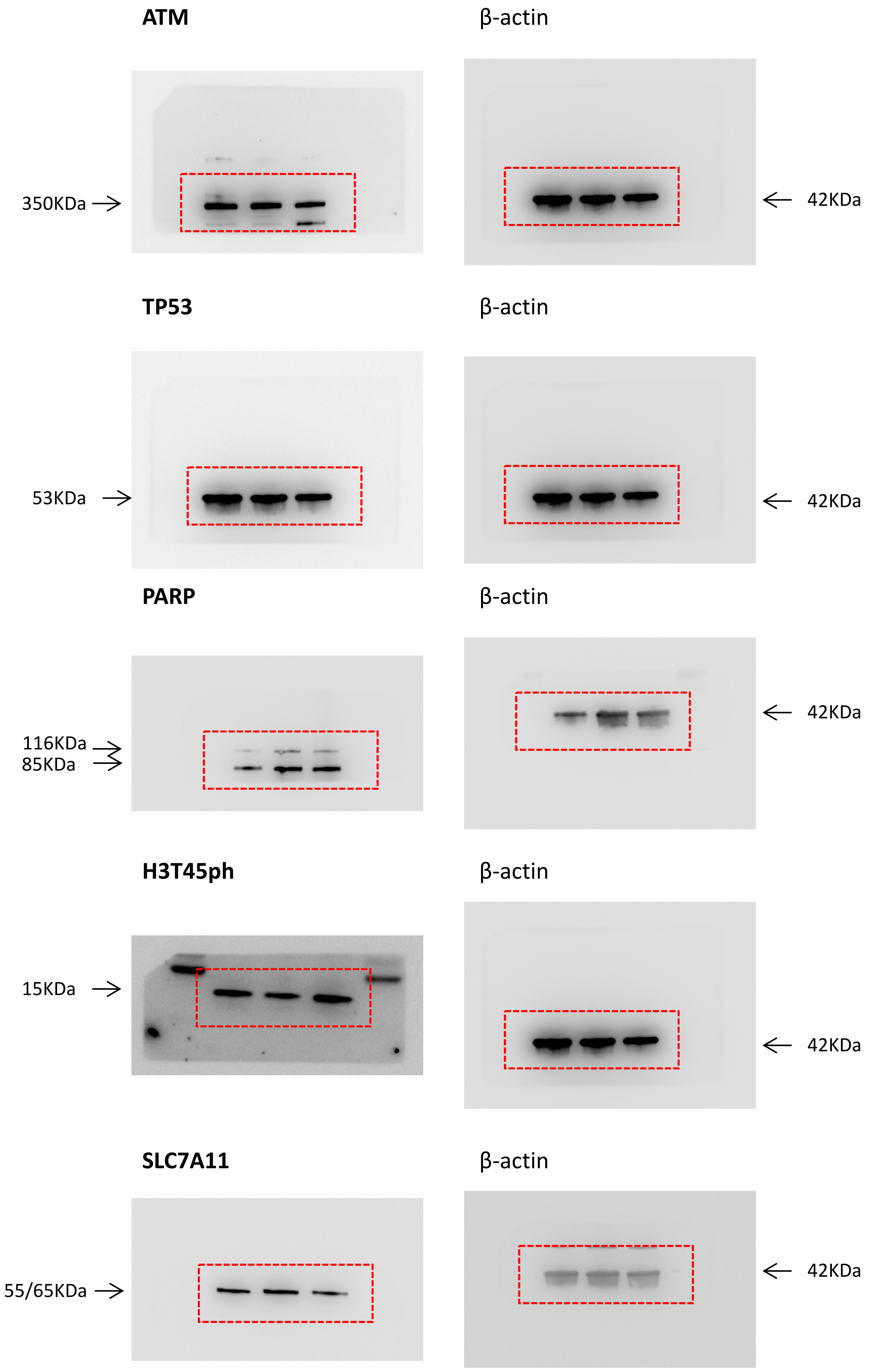

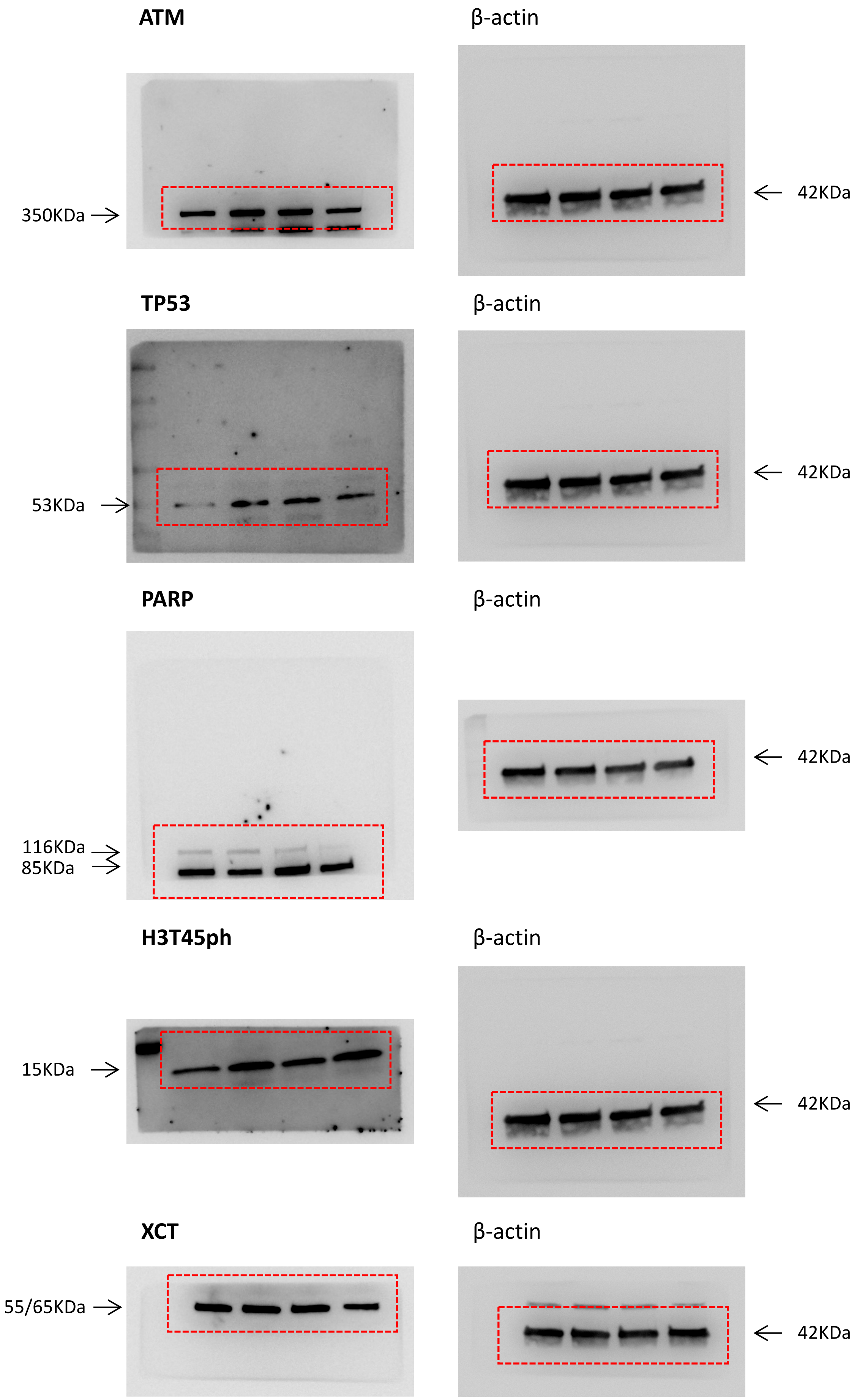
ATM
β-actin
350KDa
42KDa
TP53
β-actin
53KDa
42KDa
PARP
β-actin
116KDa
85KDa
42KDa
H3T45ph
β-actin
15KDa
42KDa
XCT
β-actin
55/65KDa
42KDa
